# Analysis of main effect QTL for thousand grain weight in European winter wheat (*Triticum aestivum* L.) by genome-wide association mapping

**DOI:** 10.3389/fpls.2015.00644

**Published:** 2015-09-01

**Authors:** Christine D. Zanke, Jie Ling, Jörg Plieske, Sonja Kollers, Erhard Ebmeyer, Viktor Korzun, Odile Argillier, Gunther Stiewe, Maike Hinze, Felix Neumann, Andrea Eichhorn, Andreas Polley, Cornelia Jaenecke, Martin W. Ganal, Marion S. Röder

**Affiliations:** ^1^Leibniz Institute of Plant Genetics and Crop Plant Research (IPK)Gatersleben, Germany; ^2^TraitGenetics GmbHGatersleben, Germany; ^3^KWS LOCHOW GmbHBergen, Germany; ^4^Syngenta France S.A.S.Orgerus, France; ^5^Syngenta Seeds GmbHBad Salzuflen, Germany

**Keywords:** association mapping, *Triticum aestivum* L., thousand grain weight, grain size, linkage disequilibrium, SSR, 90K iSELECT chip, candidate genes

## Abstract

Grain weight, an essential yield component, is under strong genetic control and at the same time markedly influenced by the environment. Genetic analysis of the thousand grain weight (TGW) by genome-wide association study (GWAS) was performed with a panel of 358 European winter wheat (*Triticum aestivum* L.) varieties and 14 spring wheat varieties using phenotypic data of field tests in eight environments. Wide phenotypic variations were indicated for the TGW with BLUEs (best linear unbiased estimations) values ranging from 35.9 to 58.2 g with a mean value of 45.4 g and a heritability of H^2^ = 0.89. A total of 12 candidate genes for plant height, photoperiodism and grain weight were genotyped on all varieties. Only three candidates, the photoperiodism gene *Ppd-D1*, dwarfing gene *Rht-B1*and the *TaGW-6A* gene were significant explaining up to 14.4, 2.3, and 3.4% of phenotypic variation, respectively. For a comprehensive genome-wide analysis of TGW-QTL genotyping data from 732 microsatellite markers and a set of 7769 mapped SNP-markers genotyped with the 90k iSELECT array were analyzed. In total, 342 significant (-log_10_ (*P*-value) ≥ 3.0) marker trait associations (MTAs) were detected for SSR-markers and 1195 MTAs (−log_10_(*P*-value) ≥ 3.0) for SNP-markers in all single environments plus the BLUEs. After Bonferroni correction, 28 MTAs remained significant for SSR-markers (−log_10_ (*P*-value) ≥ 4.82) and 58 MTAs for SNP-markers (−log_10_ (*P*-value) ≥ 5.89). Apart from chromosomes 4B and 6B for SSR-markers and chromosomes 4D and 5D for SNP-markers, MTAs were detected on all chromosomes. The highest number of significant SNP-markers was found on chromosomes 3B and 1B, while for the SSRs most markers were significant on chromosomes 6D and 3D. Overall, TGW was determined by many markers with small effects. Only three SNP-markers had R^2^ values above 6%.

## Introduction

Grain size is a yield component and part of the domestication syndrome (Peng et al., 2003) in cereals. In rice, already several genes related to grain size and grain shape have been cloned and characterized (Sakamoto and Matsuoka, 2008; Huang et al., 2013).

*GRAIN SIZE 3* (*GS3*) is a major QTL for grain length and weight, functions as a negative regulator of grain size and encodes a transmembrane protein (Fan et al., 2006; Mao et al., 2010). Syntenic genes were discovered in maize (Li et al., 2010a) and wheat (Zhang et al., 2014), where a QTL for marker *TaGS-D1* was described in a recombinant inbred population.

*GRAIN WIDTH 2 (GW2)* is a major QTL for rice grain width and weight and encodes a RING-type E3 ubiquitin ligase (Song et al., 2007). *GW2* negatively regulates cell division by targeting its substrates to proteasomes for regulated proteolysis. Loss of *GW2* function results in an increase in cell number in the spikelet hull and acceleration of the grain-milk filling rate, thus enhancing grain weight (Huang et al., 2013). Two syntenic genes in maize had effects on traits related to kernel shape and weight in an association panel (Li et al., 2010b). In wheat, a series of syntenic genes was described for all three genomes of the group 6 chromosomes (Su et al., 2011; Qin et al., 2014). Significant associations with grain weight were reported for haplotypes of the genes *TaGW2-6A* and *TaGW2-6B* (Qin et al., 2014).

A deletion in the gene *GRAIN WIDTH 5 (GW5)* had an important historical role in rice domestication (Shomura et al., 2008). *GW5* is involved in the determination of grain width and the deletion resulted in an increase in sink size owing to an increase in cell number in the outer glume.

*GRAIN SIZE 5* (*GS5*) encodes a putative serine carboxypeptidase and functions as a positive regulator of grain size (Li et al., 2011).

*GRAIN WIDTH 8 (GW8)*, synonymous to *OsSPL16* is a positive regulator of cell division and mutations in the promoter region were selected in rice breeding programs (Wang et al., 2012a).

Grain filling is affected by the *GIF1* (*GRAIN INCOMPLETE FILLING 1*) that encodes a cell-wall invertase required for carbon-partitioning during early grain-filling (Wang et al., 2008). A domestication signature was detected by comparing nucleotide diversity of the GIF1 loci between cultivated and wild rice.

A sequence-based GWAS (genome-wide association study) and functional genome annotation approach identified *OsGASR7*, a gibberellin-regulated gene that controls grain length in rice (Huang et al., 2012). The syntenic genes *TaGASR7* were discovered in *Triticum urartu* and hexaploid wheat and their natural variation could be linked to effects for grain length and grain weight (Ling et al., 2013; Dong et al., 2014).

Several candidate genes with synteny to known rice genes for grain weight, were identified in wheat and their natural variation was associated to grain size related traits in various wheat panels (Dong et al., 2014; Qin et al., 2014; Zhang et al., 2014).

There exists a wealth of QTL for grain size and shape related traits in wheat (Börner et al., 2002; Dholakia et al., 2003; Groos et al., 2003; Gupta et al., 2006; Huang et al., 2006; Gegas et al., 2010; Valluru et al., 2014; Williams and Sorrells, 2014) mostly in bi-parental mapping populations. Also in advanced backcross populations QTL for TGW were described (Huang et al., 2003, 2004; Narasimhamoorthy et al., 2006) and a TGW-QTL on chromosome 7D stemming from a synthetic wheat was fine-mapped and further characterized (Röder et al., 2008).

However, all of these studies only reflect the genetic content of a limited number of wheat accessions. Therefore, in the recent years genome-wide association mapping (GWAS) has emerged as alternative strategy to linkage mapping in bi-parental populations. Association mapping is based on “meioses of the past,” which occurred during the evolution or development of a line. The major advantages of GWAS are an increased resolution due to an increased number of recombination events compared to bi-parental mapping populations and especially the fact that larger germplasm panels can be surveyed (Hamblin et al., 2011). In specific cases, association mapping even led to the direct molecular identification of causal genes for a trait, such as a gene for spike architecture in barley (Ramsay et al., 2011).

GWAS for agronomical traits including TGW has been applied in several barley panels (Blake et al., 2012; Pasam et al., 2012; Wang et al., 2012b; Lex et al., 2014; Matthies et al., 2014). Recently, GWAS was also applied to analyze grain size related traits in wheat (Breseghello and Sorrells, 2006; Neumann et al., 2011; Wang et al., 2012c; Rasheed et al., 2014).

In the current study, we were interested to unravel the genetic architecture of the trait TGW in European winter wheat varieties. For this purpose, we conducted GWAS for TGW in a panel of 358 recent European winter wheat varieties plus 14 spring wheat varieties, which had been analyzed earlier for resistance to several fungal pathogens (Kollers et al., 2013a,b) and the agronomic traits heading date and plant height (Zanke et al., 2014a,b). The aims of the study were to identify major genetic loci for TGW, to analyze the relationship of TGW to the traits plant height and heading date, and in addition to the GWAS, to analyze the effects of 12 candidate genes on associations to TGW.

## Materials and methods

### Plant material and phenotyping

The plant material, consisting of 358 European winter wheat varieties plus 14 spring wheat varieties as an outgroup, was described in more detail in Kollers et al. (2013a). Field trials were conducted in the season 2008/2009 in Andelu/France (09.AND), Seligenstadt/Germany (09.SEL) and Wohlde/Germany (09.WOH) and in the season 2009/2010 in Andelu/France (10.AND), Janville/France (10.JAN), Saultain/France (10.SAU), Seligenstadt/Germany (10.SEL), and Wohlde/Germany (10.WOH) by applying an alpha design with two replications per site. Climatic factors and the use of growth regulators at these sites were described in Zanke et al. (2014b). Both winter and spring varieties were sown in autumn and the thousand grain weight (TGW) was measured after harvest.

For the locations in France, the TGW was determined by counting 500 grains with the counting machine “Contador,” weighing the sample and calculating the respective TGW. For the German locations, a sample of exactly 10 g was taken, the grain number was determined with the counting machine “Pfeuffer Contador” and the TGW was calculated. The grain moisture content was 14% during analysis.

### Genotyping of candidate genes and molecular markers

All varieties were genotyped with a number of molecular markers for candidate genes for TGW or yield from the literature (Supplemental File 1).

A set of 732 microsatellite markers, resulting in 770 different loci spread across all chromosomes of wheat was used for the marker-trait association analysis. Of these 770 loci, 635 loci were mapped, and 135 loci were unmapped. More details about this data set and the description of linkage disequilibrium (LD) and population structure are provided in Kollers et al. (2013a). For SNP analysis 7761 mapped markers from the 90k Infinium chip (90k iSELECT; Cavanagh et al., 2013; Wang et al., 2014) were used for genome-wide association analysis. Detailed methods applied for the genetic mapping, statistical analysis and association mapping were described elsewhere (Zanke et al., 2014a,b).

### Statistical analysis and association mapping

For all marker alleles employed in association analysis a minor allele frequency of 3% (equaling 11 varieties) was applied.

Each year-location combination was considered as an environment. For each environment adjusted entry means of the genotype were estimated using GenStat 13th edition (VSN International, Hemel Hempstead, Herfordshire, UK) and the following model:

$$\begin{array}{l} \text{y}=\mu +\text{replication}+\text{genotype}+\text{block}+\text{e} \end{array}$$

with replication and genotype as fixed factors and block as random effect nested within replication. μ represents an overall mean and e is a residual term; y represents the single plot value within each environment.

In addition, best linear unbiased estimations (BLUEs) across all environments were calculated using the software package GenStat 14th edition with:

$$\begin{array}{l} \text{y}=\mu +\text{genotype}+\text{environment}+\text{e} \end{array}$$

with genotype and environment as fixed effects; μ represents an overall mean and e is a residual term. Since the datasets for all environments were complete and balanced, the BLUEs equaled the arithmetic means across environments.

A principal component analysis (PCA) based on 10,000 SNP-markers revealed no apparent population structure among the varieties (Supplemental File 2) as was previously demonstrated in a PCA-analysis based on SSR-markers (Kollers et al., 2014). Therefore, for the analysis of marker-trait associations a mixed linear model was applied using the software package GenStat 14th edition and by applying a kinship matrix as the relationship model:

$$\begin{array}{l} \text{y}=\mu +\text{marker}+\text{genotype}+\text{e} \end{array}$$

with genotype ~ N(0,2 K$\sigma_{\text{genotype}}^{2}$), error ~ N(0,$\sigma_{\text{e}}^{2}$).

Marker refers to a fixed effect of every marker, μ represents an overall mean, e is a residual term, and K denotes the kinship matrix among all genotypes. The Loiselle kinship matrix was calculated with155 SSR-markers, equally distributed on the wheat genome, by using the software package SPAGeDi (Hardy and Vekemans, 2002). This kinship matrix was applied for calculating MTAs with SSR as well as SNP-markers as described by Matthies et al. (2012). The resulting Quantile-quantile (Q-Q) plots comparing the observed vs. expected −log_10_ (*P*-values) for two single environments and the BLUEs for association analysis with SSR-markers and SNP-markers, respectively, were depicted in Supplemental File 3.

Since many MTAs were detected, we chose an overall cutoff significance level of −log_10_ (*P*-value) ≥ 3.0, which means that one false positive is expected in one-thousand events. To ensure an even more stringent threshold, we included a second cutoff which was a Bonferroni correction for multiple testing. This threshold was calculated by dividing *P* < 0.01 with the number of SSR or SNP-markers used for the analysis and resulted in thresholds of −log_10_ (*P*-value) ≥ 4.82 for the SSR-markers and −log_10_ (*P*-value) ≥ 5.89 for SNP-markers. Additive effects and marker effects (R^2^) were estimated using the software package TASSEL 3.0, where for the bi-allelic SNP-markers the most frequent allele was set to zero and the difference of the phenotypic effect toward the less frequent allele was calculated.

Spearman rank order correlations and ANOVA using the adjusted means of the eight environments were calculated with the software package SigmaPlot 11.0. The heritability (H^2^) was calculated from the variance components according to the formula:

H^2^ = Var (genotype)/(Var (genotype) + Var (error)/no. of locations) with variance components calculated with the software package SPSS v. 19. This software was also used to conduct a trait *Post-hoc* test according to Tukey B.

## Results

### Description of phenotypic data

**Figure 1:**
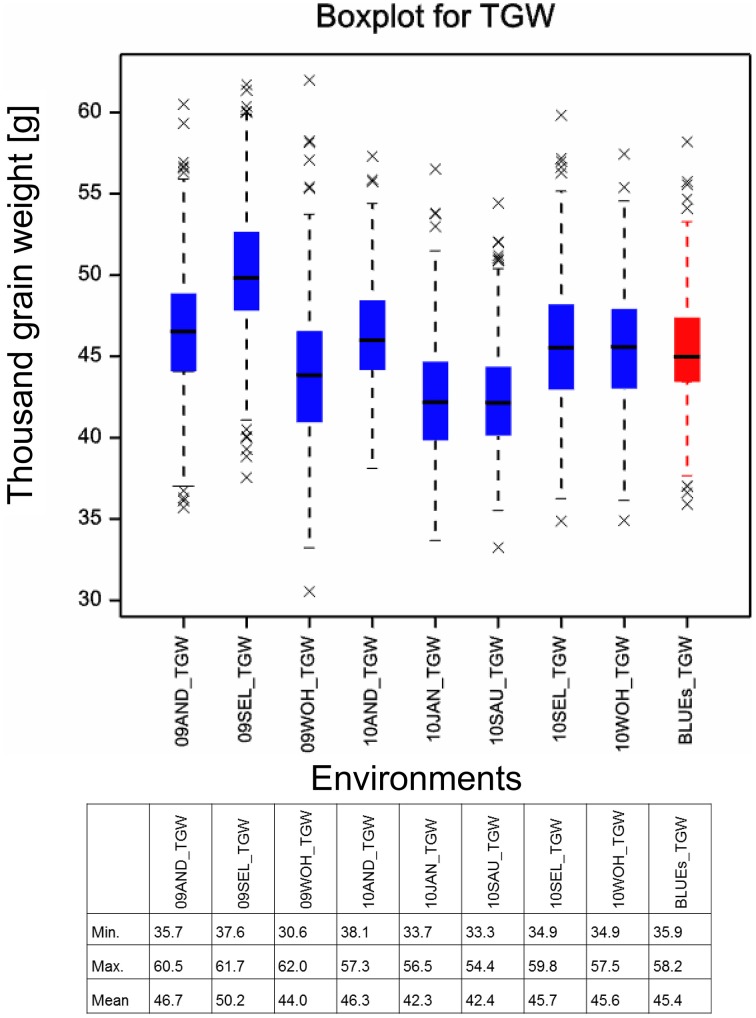
**Boxplot for thousand grain weight**. Boxplots for the eight field environments are depicted in blue, the BLUEs is shown in red. Asterisks mark outlier varieties. The table below indicates the minimal, maximal and mean TGW values measured in the single environments and estimated for the BLUEs.

**Figure 2:**
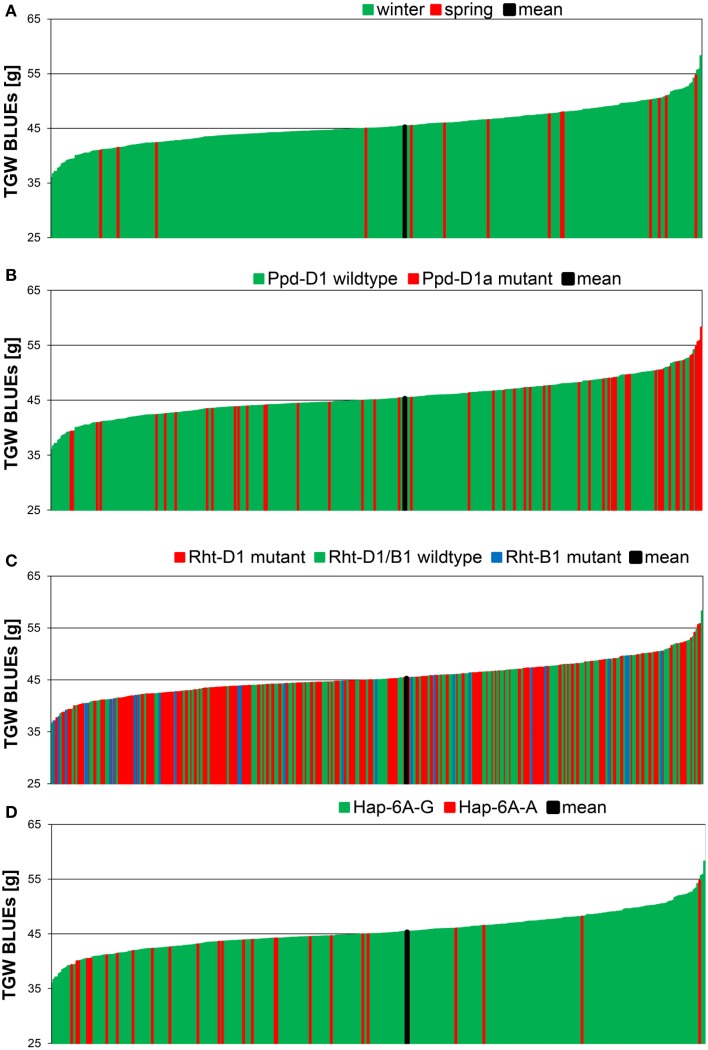
**Phenotypic distribution of TGW BLUEs in 372 wheat varieties**. The BLUEs of the TGW score were calculated across eight environments. TGW BLUEs were arranged according to the growth habit **(A)**, to the distribution of the *Ppd*-*D1* wildtype or *Ppd*-*D1a* mutant gene **(B)**, the distribution of the mutant alleles of the dwarfing genes *Rht*-B1 and *Rht*-D1 **(C)** or the distribution of the haplotypes *Hap*-6A-G and *Hap*-6A-A of the TGW candidate gene TaGW2-6A **(D)**.

The means of TGW across 358 winter wheat plus 14 spring wheat varieties in eight field environments ranged from 42.3 g in the environment 10.JAN to 50.2 g in the environment 09.SEL (Figure 1). The highest phenotypic variance was observed in environment 09.WOH, which contained the lowest single value of 30.6 g as well as the highest single value of 62.0 g. The BLUEs across all eight environments ranged from 35.9 g for variety “Carenius” to 58.2 g for variety “CCB Ingénio” and with a mean value of 45.4 g (Supplemental File 4). The continuous distribution of the TGW phenotype indicated a quantitative mode of inheritance and most spring wheat varieties were found in the second half of the distribution containing the varieties with larger grains (Figure 2A). The distribution of the TGW-BLUEs was close to a normal distribution and ranged within a 95% confidence interval in a normal probability plot (Supplemental File 5).

The Spearman rank order correlations among the eight environments ranged from 0.594 to 0.804 indicating a good reproducibility of the ranking of varieties. The correlations of individual environments with the BLUEs ranged from 0.817 to 0.898 (Supplemental File 6). The ANOVA was significant for genotypes as well as environments (Supplemental File 7). A Tukey B test discriminated five groups among the environments (Supplemental File 8). The broad sense heritability was calculated as *H*^2^ = 0.887.

### Testing of candidate genes

Besides the known dwarfing genes *Rht-B1* on chromosome 4B and *Rht-D1* on chromosome 4D and the photoperiodism gene *Ppd-D1* on chromosome 2D, a number of genes which had been published as candidates for TGW or yield mainly in Chinese germplasm were genotyped on our varieties (Table 1). These included the wheat orthologs to rice gene *OsGW2* involved in rice grain development, *TaGW2*-6A (Su et al., 2011), and *TaGW2*-6B (Qin et al., 2014) on wheat chromosomes 6A and 6B. In our material predominated the haplotype *Hap*-6A-G with a frequency of 93% for *TaGW2*-6A and haplotypes *Hap*-6B-2/3/4 with a frequency of 76% for *TaGW2*-6B.

**Table 1:** **List of tested candidate genes**.

**Gene**	**Allele 1**	**Frequency**	**Allele 2**	**Frequency**	**Allele 3**	**Frequency**	**References**
*Ppd*-D1	*Ppd*-D1 wildtype	0.86	*Ppd*-D1a^*^ mutant	0.14	–		Beales et al., 2007, TAG
*Rht*-B1	*Rht*-B1a^*^	0.93	*Rht*-B1b	0.07	–		Ellis et al., 2002, TAG
*Rht*-D1	*Rht*-D1a	0.42	*Rht*-D1b	0.58	–		Ellis et al., 2002, TAG
*TaGW2*-6A	*Hap*-6A-G^*^	0.93	*Hap*-6A-A	0.07	–		Su et al., 2011, TAG
*TaGW2*-6B	*Hap*-6B-2/3/4	0.76	*Hap*-6B-1	0.24	–		Qin et al., 2014, BMC Plant Biology
*TaGS*-D1-7D	*TaGS*-D1b	0.88	*TaGS*-D1a	0.05	TaGS-D1c	0.03	Zhang et al., 2014, Mol Breed
*TaSus*2-2A	*TaSus2*_2A_*Hap*A	0.97	*TaSus2*_2A_*Hap*G	0.00	–		Hou et al., 2014, Plant Physiol.
*TaSus*1-7A	*TaSus1*_7A_1185_*Hap*-1	0.29	*TaSus1*_7A_1185_*Hap*-2	0.66	–		Hou et al., 2014, Plant Physiol.
*TaSus*1-7A	*TaSus1*_7A_3544_*Hap*-1	0.27	*TaSus1*_7A_3544_*Hap*-2	0.68	–		Hou et al., 2014, Plant Physiol.
*TaSus*1-7B	*TaSus1*_7B_*Hap*T	0.90	*TaSus1*_7B_*Hap*C	0.05	–		Hou et al., 2014, Plant Physiol.
*TaCWI*-4A	*TaCWI*_*Hap*-4A-C	0.77	*TaCWI*_*Hap*-4A-T	0.23	–		Jiang et al., 2015, TAG
*TaCWI*-5D	*TaCWI*_*Hap*-5D-C	0.98	*TaCWI*_*Hap*-5D-G	0.01	–		Jiang et al., 2015, TAG
*TaCKX6*-D1-3D	*TaCKX6*-D1b	0.99	*TaCKX6*-D1a	0.01	–		Zhang et al., 2012, New Phytologist

* Significant for larger grain with −log_10_ (P) > 2.0.

**Figure 3:**
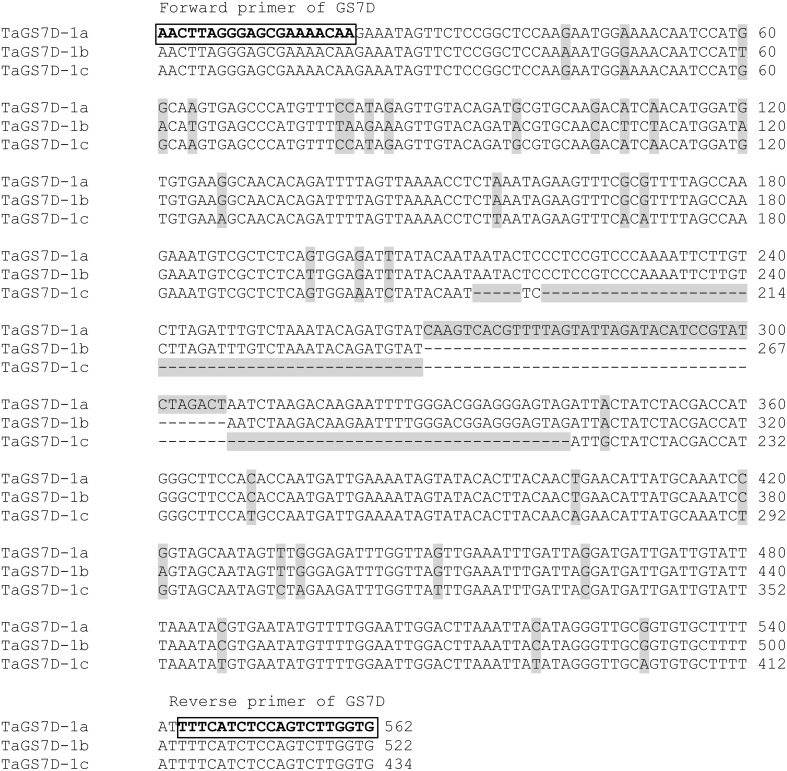
**Alignment of the alleles *TaGS*-D1a, *TaGS*-D1b and *TaGS*-D1c located on wheat chromosome 7DS**. The forward and reverse primers of GS7D used for the PCR are boxed and bold and the SNPs and InDel are shadowed.

The gene *TaGS-D1* on chromosome 7DS in wheat was described as ortholog to rice gene *OsGS3* playing a principal role in controlling grain weight and grain length in rice (Zhang et al., 2014). Genotyping of marker *TaGS-D1* of the second intron resulted besides the known alleles *TaGS-D1a* with 562 bp and *TaGS-D1b* with 522 bp, in a novel allele *TaGS-D1c* with 434 bp in 11 of our varieties (Supplemental File 4). The 40 bp-deletion of allele *TaGS-D1b* compared to *TaGS-D1a* was extended in *TaGS-D1c* to a total of 123 bp plus a small 5 bp-deletion two nucleotides upstream (Figure 3). The SNPs of allele *TaGS-D1c* resembled until nucleotide 120 the allele *TaGS-D1a* and after nucleotide 120 with one exception the allele *TaGS-D1b*, indicating that allele *TaGS-D1c* was derived from a rearrangement of the two other alleles. The most frequent allele was *TaGS-D1b* with a frequency of 88%.

Sucrose synthase haplotypes for genes *TaSus1* and *TaSus2* were associated with TGW in Chinese germplasm (Hou et al., 2014). Our germplasm was monomorphic for the favorable *TaSus2_2A_HapA* on chromosome 2A. The haplotype *TaSus1_7A_Hap-1* on chromosome 7A was detected with a frequency of 29%, while the favorable *TaSus1_7B_HapT* haplotype was present in 90% of our varieties (Table 1). Another candidate gene associated with TGW in Chinese germplasm is cell wall invertase (*TaCWI*) which hydrolyzes sucrose into glucose and fructose (Jiang et al., 2015). The favorable *TaCWI_Hap-5D-C* haplotype on chromosome 5D was present in 98% of our varieties, while for the locus on chromosome 4A haplotype *TaCWI_Hap-4A-C* was present with a frequency of 78% (Table 1).

The genotyping of *TaCKX6-D1* on chromosome 3D (Zhang et al., 2012), which is the wheat ortholog of the rice cytokinin oxidase/dehydrogenase gene *OsCKX2* (Ashikari et al., 2005), resulted in 99% of the varieties carrying haplotype B and only five varieties with haplotype A. Of those four varieties were spring varieties, indicating that the haplotype A was very rare in European winter germplasm.

All candidate genes' genotyping results in our germplasm were subjected to association analysis as far as minor allele frequencies (MAF > 3%) permitted. The highest significances were detected for photoperiodism gene *Ppd-D1* explaining up to 14.4% of phenotypic variance in a single environment (10.JAN) and 4.7% of phenotypic variance for the BLUEs with the photoperiod-insensitive *Ppd-D1a* mutant allele giving higher TGW (Supplemental File 9). This becomes also obvious in the phenotypic distribution of the *Ppd-D1a* mutant, which was mainly present in the varieties with highest TGW (Figure 2B). Of the dwarfing genes, only *Rht-B1* was significant, though the frequency of the mutant allele *Rht-B1b* was only 7%, while 58% carried the mutant allele of *Rht-D1b* (Table 1; Figure 2C). The mutant allele *Rht-B1b* decreased TGW and explained 2.3% of phenotypic variation in the BLUEs (Supplemental File 9).

Moderate significances explaining up to 3.4% of phenotypic variation in a single environment (09.AND) and 2.5% in the BLUEs were found for the *TaGW*-6A gene with the predominant haplotype *Hap*-6A-G increasing TGW (Supplemental File 9). This was in contradiction to published results where haplotype *Hap*-6A-A increased grain size in Chinese germplasm (Su et al., 2011). In the phenotypic distribution of our varieties haplotype *Hap*-6A-A was mainly present in the first half of the distribution containing the varieties with smaller grain sizes (Figure 2D). All other candidate genes failed to deliver significant associations based on a −log_10_ (*P*-value) ≥ 2.0.

### Genome-wide marker-trait associations with SSR and SNP-markers

**Figure 4:**
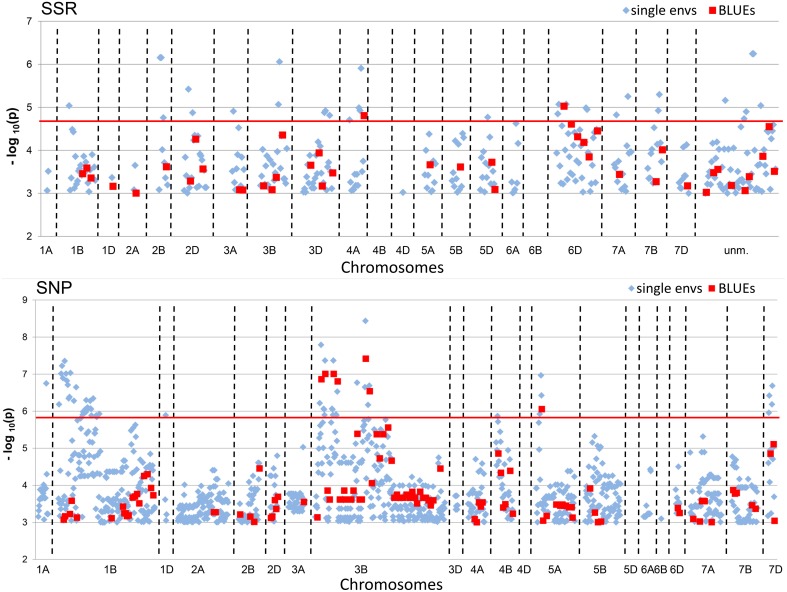
**Manhattan Plots of SSR and SNP-marker alleles associated with TGW BLUEs**. The plots present significant alleles associations at threshold −log_10_ (*P*-value) > 3.0 for the all eight single environments (depicted as blue diamonds) and BLUEs (depicted as red squares) sorted according to their chromosomal location. The red line indicates the threshold −log_10_ (*P*-value) > 4.82 for SSRs and >5.89 for SNPs, respectively, representing Bonferroni correction.

A total of 342 significant (−log_10_ (*P*-value) ≥ 3.0) MTAs were detected for SSR-markers and 1195 MTAs for SNP-markers in all single environments plus the BLUEs (Table 2). When Bonferroni-correction was applied for multiple testing, 28 MTAs remained significant for SSR-markers (−log_10_ (*P*-value) ≥ 4.82) and 58 MTAs for SNP-markers (−log_10_ (*P*-value) ≥ 5.89) (Table 2, Supplemental Files 10, 11). MTAs were present on all chromosomes except chromosomes 4B, 4D, and 6B for SSR-markers and chromosomes 4D and 5D for SNP- markers (Figure 4; Supplemental Files 12, 13). The highest number of significant SNP- markers were found on chromosomes 3B and 1B, while for the SSRs most significant markers were found on chromosomes 6D and 3D, representing the better coverage of the D-genome by the SSRs compared to the SNPs.

**Table 2:** **Number of MTAs per environment for the SSR-markers and the SNPs of the 90K iSelect chip**.

**Environments**	**SSR**		**90K iSelect**	
	**−log_10_ (*P*-value) > 3.0**	**−log_10_ (*P*-value) > 4.82**	**−log_10_(*P*-value) > 3.0**	**−log_10_ (*P*-value) > 5.89**
Andelu (2009)	31	3	77	0
Seligenstadt (2009)	40	4	111	0
Wohlde (2009)	43	8	215	27
Andelu (2010)	23	2	124	3
Janvielle (2010)	58	6	192	7
Saultain (2010)	33	0	125	2
Seligenstadt (2010)	33	1	123	4
Wohlde (2010)	38	3	113	8
BLUEs	43	1	115	7
Sum	342	28	1195	58

### Additive effects for the “Best” and “Worst” alleles

Of the 342 significant MTAs based on SSR-markers, 161 had a negative additive effect decreasing grain size and 181 had a positive effect increasing grain size (Supplemental File 10A). Considering the BLUEs, 20 SSR-markers were found with negative additive effect and 23 SSR-markers with positive additive effect (Supplemental File 10B). The number of significant MTAs for the SNP-markers was 1195, 867 MTAs with negative additive effect and 328 MTAs with positive additive effects (Supplemental File 11A), and only for the BLUEs there were 85 SNP-markers with negative effect and 30 SNP-markers with positive effect (Supplemental File 11B).

**Figure 5:**
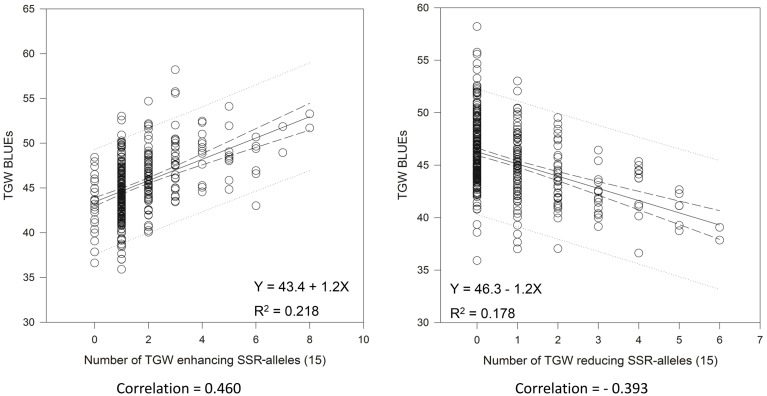
**Linear regressions of the most TGW promoting (“best”) and the most TGW reducing (“worst”) SSR-alleles with TGW-BLUEs**. Linear regression resulted in a relationship between TGW-BLUEs and the 15 “best” or “worst” SSR-alleles in 372 varieties.

We tried to extract the 15 “best” and 15 “worst” markers for SSRs and SNPs, respectively, by choosing the markers with the largest positive or negative additive effects based on the BLUEs, that means the markers with on average having the biggest phenotypic effects in increasing or decreasing grain size. Co-locating or very closely linked markers were omitted (Tables 3, 4). The wheat varieties carried between zero and eight of the 15 “best” TGW enhancing alleles and between zero and six of the 15 “worst” TGW-reducing SSR alleles. The Spearman Rank correlations for the number of “best” or “worst” SSR-alleles per variety with TGW-BLUEs were 0.460 (*P* = 0.001) or −0.393 (*P* = 0.001), respectively. The fit for linear regression with TGW-BLUEs was Y = 43.4 +1.2X with R^2^ = 0.218 for the 15 “best” alleles and Y = 46.3 −1.2X with R^2^ = 0.178 for the 15 “worst” alleles (Figure 5). This means that varieties with many positive alleles and few negative alleles have the highest TGW and that the effects of alleles are at least partially additive.

**Table 3:** **List of the most TGW enhancing (“best”) and most TGW reducing (“worst”) SSR-alleles**.

**Marker alleles**	**Chromosome**	**Position (cM)**	**Alleles belong to the**	
			**15 best**	**15 worst**
GWM0124_197^*^	1B	131.3	x	
GWM0124_213^*^	1B	131.4		x
WMC0429_250	1D	93.2		x
GWM1128_149	2B	16.1		x
GWM261_188	2D	38.3	x	
GWM0539_151	2D	136	x	
BARC0012a_201	3A	19.1	x	
BARC0197_174	3A	125	x	
WMC0808_147	3B	67.8	x	
GWM0566_131	3B	89.2		x
GWM0376c_134	3B	110.1	x	
WMC0366_103	3B	111.7		x
GWM4804a_111	3D	67.3		x
WMC0283b_168	4A, 7A	163.6/65.2		x
BARC0330_105	5A	125.4	x	
WMC0415b_176	5B	97.6	x	
WMC0215_212	5D	200.9	x	
WMC0672b_98	6A	67.5		x
WMC0553_121	6A	104.4		x
GWM0469_176	6D	65.7	x	
GWM1749_130	6D	222.7		x
GWM0631_203	7A	132.3		x
WMC0607b_266	7A	154.6	x	
WMC0396_195	7B	55.7		x
GWM1276_205	7D	201	x	
WMC0009_199	Unmapped	–		x
WMC0419_155	Unmapped	–	x	
WMI0042_184	Unmapped	–	x	
WMJ3100_166	Unmapped	–		x
BARC0131_225	Unmapped	–		x

* Markers with positive and negative additive effects.

**Table 4:** **List of the most TGW enhancing (“best”) and most TGW reducing (“worst”) SNP-alleles**.

**Marker alleles**	**Chromosome**	**Position (cM)**	**Alleles belong to the**	
			**15 best**	**15 worst**
RAC875_c95364_259	1A	0.0		x
Kukri_c76307_182	1B	12.6	x	
D_contig17842_656	1B	41.3	x	
Tdurum_contig48523_1838	1B	54.0		x
Tdurum_contig8580_586	1B	84.9		x
wsnp_Ex_c1358_2602235	1D	12.1	x	
Excalibur_c80601_278	2B	87.5		x
BS00031098_51	2B	135.5		x
IACX5899	3A	84.7		x
Kukri_c21467_571	3B	44.9		x
Tdurum_contig59953_220	3B	94.8	x	
Kukri_c24488_431	3D	3.2	x	
RAC875_c56535_256	4A	111.1	x	
Kukri_c20012_1425	4A	111.9		x
Kukri_c56014_275	4A	120.4		x
RFL_Contig2531_1872	4A	150.6		x
Kukri_rep_c104941_336	4B	63.3	x	
wsnp_CAP11_c951_572693	5A	11.0	x	
BS00076190_51	5A	16.7	x	
wsnp_Ex_c40019_47166980	5A	65.6	x	
IAAV8042	5A	65.6		x
RAC875_c8642_231	5A	114.5	x	
tplb0057m18_546	6A	56.3		x
Tdurum_contig27888_760	6A	86.9		x
Ex_c7086_187	6D	118.4	x	
BS00040601_51	7A	78.7		x
wsnp_Ra_c4418_8012732	7A	80.3	x	
Ex_c12057_797	7B	68.6		x
Kukri_c15768_68	7D	126.5	x	
Ra_c73114_242	7D	177.6	x	

**Figure 6:**
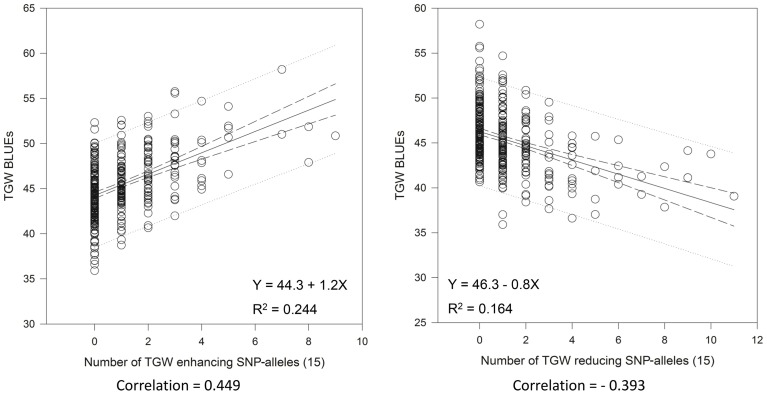
**Linear regressions of the most TGW promoting (“best”) and the most TGW reducing (“worst”) SNP-alleles with TGW-BLUEs**. Linear regression resulted in a relationship between TGW-BLUEs and the 15 “best” or “worst” SNP-alleles from different loci in 372 varieties.

Very similar results were obtained with the 15 “best” and “worst” SNP-alleles, indicating that both marker systems are comparable in their power for marker assisted selection (Figure 6). There were between zero and nine of the “best” TGW enhancing SNP-alleles, and between zero and eleven of the “worst” TGW SNP-reducing alleles present per variety. The respective Spearman Rank correlations for the number of “best” or “worst” SNP-alleles per variety with TGW-BLUEs were 0.449 (*P* = 0.001) and −0.393 (*P* = 0.001). Linear regressions with TGW-BLUEs were Y = 44.3+1.2X with *R*^2^ = 0.244 for the 15 “best” alleles and Y = 46.3 −0.8X with R^2^ = 0.164 for the 15 “worst” alleles (Figure 6).

### Effects of plant height and heading date on TGW

In previous publications, the traits of heading date and plant height were investigated in the same set of varieties (Zanke et al., 2014a,b). In different environments, the Spearman rank order correlations for TGW and heading date ranged between −0.528 and −0.140 and the correlations between TGW and plant height ranged from 0.101 to 0.319. For the BLUEs values across all eight environments the correlation coefficients were −0.330 for heading date and 0.248 for plant height with TGW (Table 5). This means on average earlier heading and taller varieties had larger grain size. This observation is in agreement with the MTAs found for the candidate genes *Ppd-D1* and *Rht-B1*, where the earlier heading *Ppd-D1a* mutant allele and the *Rht-B1a* wild type allele explained larger TGW.

**Table 5:** **Correlations of TGW values of respective environments to plant height (PH) and heading date (HD)**.

	**09.AND.HD**	**09.SEL.HD**	**09.WOH.HD**	**10.AND.HD**	**10.JAN.HD**	**10.SAU.HD**	**10.SEL.HD**	**10.WOH.HD**	**BLUES_HD**
09.AND.TGW	−0.338^***^								
09.SEL.TGW		−0.356^***^							
09.WOH.TGW			−0.528^***^						
10.AND.TGW				−0.177^**^					
10.JAN.TGW					−0.429^***^				
10.SAU.TGW						−0.220^***^			
10.SEL.TGW							−0.251^***^		
10.WOH.TGW								−0.140^*^	
BLUES_TGW									−0.330^***^
	**09.AND.PH**	**09.SEL.PH**	**09.WOH.PH**	**10.AND.PH**	**10.JAN.PH**	**10.SAU.PH**	**10.SEL.PH**	**10.WOH.PH**	**BLUES_PH**
09.AND.TGW	0.180^**^								
09.SEL.TGW		0.272^***^							
09.WOH.TGW			0.150^*^						
10.AND.TGW				0.172^**^					
10.JAN.TGW					0.101				
10.SAU.TGW						0.278^***^			
10.SEL.TGW							0.319^***^		
10.WOH.TGW								0.291^***^	
BLUES_TGW									0.248^***^

* P < 0.01.

** P < 0.001.

*** P < 0.0001.

We investigated the occurrence of SNP- and SSR-markers which detected significant MTAs for two traits, either TGW and heading date or TGW and plant height (Table 6, Supplemental Files 14, 15). The positive correlation observed between TGW and plant height was supported by six SNP and three SSR-alleles which detected MTAs in both traits and had in both traits positive additive effects (Table 6). For heading date, eight significant SNP-markers had positive additive effects for both traits and three SNP-markers accounted for negative additive effects in both traits, which was in contradiction to the observations of the correlation analysis. This can be explained, that the negative correlations between heading date and TGW are most likely mainly an effect of the *Ppd-D1*-alleles, and the other markers had only minor effects on the correlation results.

**Table 6:** **Number of alleles relevant in multiple traits**.

	**HD pos**	**HD neg**	**PH pos**	**PH neg**
TGW positive	8	0 (2)	6 (3)	0
TGW negative	1 (1)	3	2	0

The numbers refer to SNP-alleles. The numbers for SSR-alleles including the Ppd-D1 alleles are given in brackets. HD pos, heading date alleles (LOD ≥ 3.0) with positive additive effect (means later heading); HD neg, heading date alleles (LOD ≥ 3.0) with negative additive effect (means earlier heading); PH pos, plant height alleles (LOD ≥ 4.0) with positive additive effect (means taller plants); PH neg, plant height alleles (LOD ≥ 4.0) with negative additive effect (means shorter plants); TGW positive, thousand kernel weight alleles (LOD ≥ 3.0) with positive additive effect (means larger grain); TGW negative, thousand kernel weight alleles (LOD ≥ 3.0) with negative additive effect (means smaller grain).

## Discussion

Our genome-wide association mapping approach provided a comprehensive overview about the genetic architecture of the trait TGW in European winter wheat varieties. With the employed statistical model using a kinship matrix for population stratification correction, a total of 342 MTAs with (−log_10_ (*P*-value) ≥ 3.0) for SSRs and 1195 MTAs with (−log_10_ (*P*-value) ≥ 3.0) for SNPs were discovered. The quantile-quantile-plots (Supplementary File 3) indicated that the employed model did not completely correct for potential false positives, since the observed values deviated from the expected diagonal. Therefore, a second more stringent significance threshold, corresponding to the Bonferroni correction for multiple testing, was introduced. It resulted in 28 significant for SSRs (−log_10_ (*P*-value) ≥ 4.82) and 58 significant MTAs for SNPs (−log_10_ (*P*-value) ≥ 5.89). Nevertheless, we kept the MTAs with (−log_10_ (*P*-value) ≥ 3.0) in the description of results, because less stringent MTAs may support the MTAs detected above the Bonferroni threshold when discovered in independent environments or with co-segregating or closely linked markers.

The highest number of significant MTAs was discovered on chromosome 3B (Supplemental File 12) which contained at least three distinctive significant chromosomal regions on the SSR-map as well as on the SNP-map (Supplemental File 13). A prominent QTL for yield, TGW and early vigor was described on chromosome 3BL in multiple environments in the bi-parental mapping population RAC875/Kukri (Bennett et al., 2012; Bonneau et al., 2013). The location of this yield QTL in the marker interval GWM114 and wPT-4401 was near to marker WMC632 which was significant in our dataset. Also Maphosa et al. (2014), Börner et al. (2002) and Wang et al. (2012c) reported TGW-QTL in various types of germplasm at similar locations on chromosome 3BL. SNP-marker Tdurum_contig59953_220 was among the “best” SNP-markers based on the additive effects (Table 4). Though Tdurum_contig59953_220 is located in a similar mapping position like WMC632, no linkage disequilibrium (LD) between both markers exist (data not shown), which indicates that they target different QTL.

SSR-marker GWM376 was among the “best” SSR-markers based on the additive effects (Table 3). Its location in the interval of GWM685 and GWM802 coincided with a QTL for TGW (Huang et al., 2004) and a QTL for yield (Huang et al., 2003) described in two advanced backcross populations. The two significant markers WMC675 and WMC612 in our study coincided with the location of a second QTL for TGW and yield on chromosome 3B in the RAC875/Kukri population (Bennett et al., 2012). The allele WMC675b_160 bp was significant in several environments and it was in the LD with a cluster of SNP-markers ranging from wsnp_Ku_c24414_34372822 to BS00091577_51 (Supplemental File 16).

A TGW-QTL on chromosome 3BS in the Drysdale/Gladius bread wheat mapping population distal of marker BARC87 (Maphosa et al., 2014) mapped to a similar position like the two significant markers BARC133 and BARC147 in our study.

A high number of co-segregating SNP-markers were significant on chromosome 1BS in most cases for only one environment, i.e., 09WOH. This QTL site co-located with a cluster of QTL for various kernel morphology and seed weight traits in the tetraploid mapping population Simeto × Molise Colli (Russo et al., 2014). In our SSR-map the significant marker GWM413 corresponded to the respective SNP-cluster. For GWM413 a QTL for seed length was discovered in a bi-parental mapping population (Tyagi et al., 2015). SSR-marker GWM124 on chromosome 1BL is among the “best” SSR-markers for TGW (Table 3). For the closely linked marker GWM268 a yield-QTL was described in an advanced backcross population (Huang et al., 2003; Wang et al., 2012c) identified MTAs for TGW in Chinese wheat germplasm for GWM268.

On chromosome arm 5AS, SNP-marker BS00076190_51 was highly significant in several environments including BLUEs and among the “best” SNP-markers based on the additive effects (Table 4). The same marker was also significant in plant height (Supplemental File 15) (Zanke et al., 2014b) and had an increasing effect on both of traits, TGW and plant height. On chromosome 5AL, marker wsnp_Ex_c40019_47166980 was among the “best” markers based on the additive effects (Table 4). It co-segregated with marker BobWhite_c14689_172, which was also significant for TGW in a GWAS analysis of an elite spring wheat population (Sukumaran et al., 2015). SSR-marker BARC330 on chromosome 5AL was among the “best” SSR-markers and MTAs for TGW for this marker were described also by Wang et al. (2012c).

The highest number of significant MTAs for the SSR-markers was found on chromosome 6D (Supplemental File 12). The SSR-map as well as the SNP-map comprised two major loci, one on the short arm and one on the long arm of chromosome 6D (Supplemental File 13). SSR-marker GWM1749 was among the “worst” markers with a TGW reducing additive effect (Table 3). A QTL for yield was reported for the closely linked marker GDM98 (Huang et al., 2003). The locus on chromosome 6DS comprised of five highly significant SSR-markers with MTAs in multiple environments including BLUEs. Marker GWM469 was among the “best” SSR-markers for positive additive effects (Table 3). We did not find a matching QTL in the literature for this major TGW-locus. A meta-QTL described by Tyagi et al. (2015) for the interval CFD19c—GWM325 did not coincide with the QTL-locus discovered in our data on chromosome 6DS.

The significant SSR-marker GWM1397 located in the interval of BARC126 andGWM44 on chromosome 7DS coincided with the mapping of a TGW-QTL in two advanced backcross populations (Huang et al., 2003, 2004) which was later fine-mapped and confirmed (Röder et al., 2008).

SSR-marker WMC533 on chromosome 3D was significant in several environments including BLUEs. The gene *TaCKX6-D1*, encoding a cytokinin oxidase/dehydrogenase (*CKX*), was reported to be closely linked to WMC533 (Zhang et al., 2012). Haplotypes of *TaCKX6-D1* were associated with grain weight in a panel of Chinese wheat germplasm (Zhang et al., 2012), however in our germplasm only five deviating haplotypes were discovered and no significant association was detected, even when the applied minor allele frequency of 3% was not considered. Therefore, the MTAs at WMC533 and the neighboring markers are most likely caused by another gene.

Of 12 tested candidate genes, only the photoperiodism gene *Ppd-D1*, the dwarfing gene *Rht-B1* and the grain weight gene *TaGW2-6A* were significant (Table 1). It is interesting to note that the mutant allele *Rht-B1b* on chromosome 4B was present in much lower frequency than the mutant allele of *Rht-D1b* on chromosome 4D, and that in a previous study about GWAS in plant height only candidate gene *Rht-D1* had been found significant (Zanke et al., 2014b).

A major and stable QTL for yield and grain weight on chromosome 6A, where *TaGW2-6A* is located, was detected in the mapping population Spark × Rialto and confirmed in nearly isogenic lines (Simmonds et al., 2014). Gene *TaGW2-6A* is a possible candidate for this effect, however sequence polymorphisms for the respective mapping parents were only discovered in the promoter region of the gene.

All other genes, which were considered as candidates for grain weight, were previously mainly identified in Chinese germplasm (Table 1). For gene *TaGS-D1* we found a novel allele which was not described before (Figure 3). Our results are in accordance with the study of Mohler et al. (2015) who found that the alleles increasing TGW of the analyzed genes are either almost fixed or not exploited yet in the European winter wheat germplasm.

Only few markers simultaneously detected significant MTAs for TGW and plant height or heading date (Table 6, Supplemental Files 14, 15), though some significant correlations among these traits were detected (Table 5). Earlier heading may cause an extended period of grain filling resulting in larger grains. This effect seemed to be mainly explained by the *Ppd-D1a* mutant allele.

Overall, the trait of TGW was determined by many MTAs with small effects. The R^2^ values for most significant MTAs ranged from 2 to 3% or were even smaller. Only three SNP-markers had R^2^ values above 6%, i.e., BobWhite_c10402_140 and wsnp_JD_c2623_3541255 co-segregating on chromosome 3BS and BS00076190_51 on chromosome 5A (Supplemental File 11). Nevertheless, 15 chosen SNP-markers or SSR-markers were sufficient to achieve highly significant correlations between the number of TGW-enhancing alleles present in a variety and the TGW-BLUEs (Figures 5, 6) indicating that the alleles are at least partially additive and could be pyramided in a variety.

## Conclusion

It can be concluded that for wheat, in contrast to rice, no genes for grain size have been identified on a molecular base yet. Of possible candidate genes, only the *TaGW*-6A gene gave moderate significances for the trait TGW in an association panel of European winter wheat varieties. While a wealth of significant MTAs for TGW was detected, a locus on chromosome 3BS and another locus on chromosome 5AS had high R^2^-values indicating linkage disequilibrium to genes with strong effects on grain size in wheat. The same marker on chromosome 5AS was also strongly linked to plant height.

In terms of breeding, grain size, besides grain number per plant, is an important yield component, though large grain size does not necessarily result in higher yields. Nevertheless, our data indicated that pyramiding alleles of markers positively associated with grain size could result in wheat varieties with increased TGW.

### Conflict of interest statement

Sonja Kollers, Viktor Korzun, and Erhard Ebmeyer are employed by the company KWS LOCHOW GMBH, Odile Argillier is employed at Syngenta France S.A.S., Gunther Stiewe and Maike Hinze are or were employed by Syngenta Seeds GmbH, Jörg Plieske, Andreas Polley, Andrea Eichhorn and Felix Neumann are or were employed by the company TraitGenetics GmbH. The companies have commercial interest in the results for application in variety development and for the provision of genotyping services. This does not alter the authors' adherence to all Frontiers policies on sharing data and materials. The authors declare that the research was conducted in the absence of any commercial or financial relationships that could be construed as a potential conflict of interest.
