# Supplementary material for: Analysis of main effect QTL for thousand grain weight in European winter wheat (Triticum aestivum L.) by genome-wide association mapping
Source: Front Plant Sci. 2015 Sep 1;6:644. doi: 10.3389/fpls.2015.00644 (PMC4555037; doi:10.3389/fpls.2015.00644)
Supplement: Supplementary file 1 [file DataSheet1.ZIP › Supplementary/152871_Röder_Data_Sheet_1.PDF]

Supplemental file 1: Methods and primers for analysis of candidate genes.

The different alleles of the *TaGW2-6A* gene (Su et al. 2011) and the *TaGW2-6B* gene (Qin et al. 2014) were identified with CAPS (cleaved amplified polymorphic sequence) markers based on polymorphism detected in the promotor regions of these genes. For gene *TaGW2-6A*: forward primer 5'-GAGAAAGGGCTGGTGCTATGGA-3' and reverse primer 5'-GTAACGCTTGATAAACATAGGTAAT-3', PCR products were digested with *Taq*I, separated on 2% agarose gels according to Su et al. (2011) and generated restriction fragments of 167 bp for *Hap*-6A-A and 218 bp for *Hap*-6A-G, respectively; for *TaGW2-6B*: forward primer 5'-GACTCCTCCTCGTCACCCATAAAGT-3' and reverse primer 5'-ATAGCACCAGCCCTTTCTCTTC-3', PCR products were digested with *BstN*I, separated on 1.5% agarose gels according to Qin et al. (2014), whereby *Hap*-6B-1 (no restriction sites) was represented by a single 1.4 kb fragment, whereas *Hap*-6B-2/3/4 was digested into 1.0 Kb and 400 bp fragments.

For the discrimination of the different alleles of the gene *TaGS-D1-7D* (Zhang et al. 2014) the co-dominant STS marker, designated GS7D, was used based on a 40-bp InDel (insertion/deletion) present in the second intron of the gene. According to Zhang et al. (2014) with forward primer 5'-AAC TTA GGG AGC GAA AAC AA-3' and reverse primer 5'-CAC CAA GAC TGG AGA TGA AA-3', this marker amplified a 562bp fragment from the genotype with the allele *TaGS-D1*a, and a 522bp fragment from the genotype with the allele *TaGS*-D1b and an additional 434bp fragment for the genotype with a new allele named *TaGS-D1*c.

One CAPS marker based on the SNP identified in the first exon of the *TaSus2-2A* gene (Hou et al. 2014) was applied to differentiate the two alleles of this gene. According to Hou et al. (2014) the PCR product of *Hap*-A amplified by the genome-specific forward primer 5'-GCAATAGTTCCGTGCTCCTGTG-3' and reverse primer 5'-AGAAATACGCAAGG CAACCAT-3' was 516 bp before enzyme digestion with *Asc*I, and the products of *Hap*-G were 322 and 194 bp after enzyme digestion.

By using two CAPS marker based on SNP 1,185 within intron 4 and SNP 3,544 within exon 13, respectively, the haplotypes *Hap*-1 and *Hap*-2 of *TaSus*1-*7A* gene (Hou et al. 2014) were identified. For the SNP 1,185 the forward primer 5'-GCTGCTCAGGTAACATAACATCGT-3', the reverse primer 5'-CAACCTGTTAAACAATATCCTGT-3' and the restriction enzyme *Taq*I

produced four fragments for *Hap*-1 and only three fragments for *Hap*-2 according to Hou et al. (2014). For the SNP 3,244 the forward primer 5'-CTCACA TTGCTTTCACCCTCC-3', the reverse primer 5'-GCAGGACAAATATTAGAATCCG-3' and the restriction enzyme *ApaL*I showed 908bp and 152bp fragments for *Hap*-1 and only one 1060bp fragment for *Hap*-2 according to Hou et al. (2014).

For the *TaSus1-7B* gene (Hou et al. 2014) the CAPS markers at the InDel site within the intron 11 using restriction endonucleases *Sph*I distinguished the two haplotypes of the gene. The PCR reaction with the forward primer 5'-GATTGTTGGCTGTGACAGTATTAA-3' and reverse primer 5'-CTGGCAGGACAAATATTAGAATG-3' according to Hou et al. (2014) amplified for Hap-C a 1,124bp fragment after restriction digestion by *Sph*I, whereas *Hap*-T produced two fragments with lengths of 730bp and 394bp, respectively.

The molecular analysis of the varieties with regard to the candidate genes *TaCWI-4A* and *TaCWI-5D* was carried out with CAPS marker according to Jiang et al. (2015). For *TaCWI-4A* the genome specific forward primer 5'- AGGGCGTCCGACCAAAGTG-3' and reverse primer 5'-GCGACCTAGCGTGTATCAAGGAG-3' amplified an 885bp fragment which was cut by the restriction enzyme *Tai*I into a 354 and a 531bp fragment within the haplotype *Hap*-4A-C, but not cut within the haplotype *Hap*-4A-T. A 649bp fragment was amplified by the forward primer 5'-CATGTGCCTCTAAAATTAGGTTATG-3' and the reverse primer 5'-CGCCCA GATGATGTTCC-3' from *TaCWI-5D*. The restriction enzyme *BstY*I cut *Hap*-5D-G amplicons into three fragments of 409, 143 and 97 bp, whereas Hap-5D-C was cut into two fragments of 405 and 244 bp.

For the *TaCKX6-D1* gene (Zhang et al. 2012) the developed indel marker with the forward primer 5'-CACGTCGATAGTCTCATGCA-3' and the reverse primer 5'-CAGGAACTCCACGTAAGACA-3' detected the 18bp indel polymorphism in the second intron of the gene and was used to characterize haplotypes A and B of *TaCKX6-D1*.

All cultivars were additionally genotyped for the dwarfing genes *Rht-B1* and *Rht-D1* (Ellis et al. 2002) and the *Ppd-D1a* allele of the photoperiod response locus *Ppd-D1* (Beales et al. 2007) according to the methods described in an earlier study (Kollers et al. 2014).

Supplemental table 1: Primers and methods for genotyping of candidate genes.

Gene	forward primer	reverse primer	Marker / Enzyme	Allele1	fragment size	Allele2	fragment size	Allele 3	fragment size
Ppd-D1	5'-ACGCCTCCCACTACACTG-3'	5'-GTTGGTTCAAACAGAGAGC-3' and 5'-CAC TGG TGG TAG CTG AGA TT – 3'	indel marker	*Ppd*-D1 wildtype	414 bp / 2 kbp indel present	*Ppd*-D1a* mutant	288 bp / 2 kbp indel absent	-	
Rht-B1 wild type	BF: 5'-GGTAGGGAGGCGAGAGGCGAG-3'	WR1: 5'-CATCCCCATGGCCATCTCGAGCTG-3'		*Rht*-B1a	237 bp	*Rht*-B1b	-	-	
Rht-B1 mutant	BF: 5'-GGTAGGGAGGCGAGAGGCGAG-3'	MR1: 5'-CATCCCCATGGCCATCTCGAGCTA-3'			-	*Rht*-B1b	237 bp		
Rht-D1 wild type	*DF2: 5'-GGCAAGCAAAAGCTTCGCG-3'*	*WR2: 5'-GGCCATCTCGAGCTGCAC-3'*		*Rht*-D1a	264 bp	*Rht*-D1b	-	-	
Rht-D1 mutant	*DF: 5'-CGCGCAATTATTGGCCAGAGATAG-3'*	*MR2: 5'-CCCCATGGCCATCTCGAGCTGCTA-3'*		*Rht*-D1a	-	*Rht*-D1b	254 bp		
TaGW2-6A	5'-GAG AAAGGGCTGGTGCTATGGA-3'	5'-GTAACGCTTGATAAACAT AGGTAAT-3'	CASP Marker / *Taq*I,	*Hap*-6A-G*	218 bp	*Hap*-6A-A	167 bp	-	
TaGW2-6B	5'-GACTCCTCCTCGTC ACCCATAAAGT-3'	5'- ATAGCACCAGCCCTTTCTCTTC-3'	CASP Marker / *BstN*I	*Hap*-6B-2/3/4	400 bp and 1 kbp	*Hap*-6B-1	1 kbp (no cut)	-	
TaGS-D1-7D	*5'-AAC TTA GGG AGC GAA AAC AA-3'*	*5'-CAC CAA GAC TGG AGA TGA AA-3'*	STS marker	*TaGS*-D1b	522bp	*TaGS*-D1a	562bp	TaGS-D1c	434bp
*TaSus*2-2A	*5' GCAATAGTTCCGTGCTCCTGTG-3'*	5'-AGAAATACGCAAGG CAACCAT-3'	CAPS marker / *Asc*I	*TaSus2*_2A_*Hap*A	516 bp	*TaSus2*_2A_*Hap*G	322 and 194 bp	-	
*TaSus*1-7A	5'-GCTGCTCAGGTA ACATAACATCGT-3'	5'-CAACCTGTTAAACAATATCCTGT-3	CASP / *Taq*I	*TaSus1*_7A_1185_*Hap*-1	4 fragments	*TaSus1*_7A_1185_*Hap*-2	3 fragments	-	
*TaSus*1-7A	*5'-CTCACA TTGCTTTCACCCTCC-3'*	*5'-GCAGGACAAATATTAGAATCCG-3'*	CASP / *ApaL*I	*TaSus1*_7A_3544_*Hap*-1	908 bp and 152 bp	*TaSus1*_7A_3544_*Hap*-2	1,060 bp	-	
*TaSus*1-7B	5'-GATTGTTGGCTGTGACAGTAT TAA-3'	5'-CTGGCAGGACAAATATTAGAATG-3'	CASP / *Sph*I	*TaSus1*__7B_*Hap*T	730 bp and 394 bp	*TaSus1*_7B_*Hap*C	1,124 bp	-	
TaCWI-4A	*5'- AGGGCGTCCGACCAAAGTG-3'*	*5'-GCGACCTAGCGTGTATCAAGGAG-3'*	CASP / *Tai*I	*TaCWI*_*Hap*-4A-C	354 bp and 531 bp	*TaCWI*_*Hap*-4A-T	885 bp	-	
TaCWI-5D	*5'-CATGTGCCTCTAAAATTAGGTTATG-3'*	*5'-CGCCCA GATGATGTTCC-3'*	CASP / *BstY*I	*TaCWI*_*Hap*-5D-C	405 bp and 244 bp	*TaCWI*_*Hap*-5D-G	409 bp 143 bp and 97 bp	-	
TaCKX6-D1-3D	*5'-CACGTCGATAGTCTCATGCA-3'*	*5' CAGGAACTCCACGTAAGACA-3'*	indel marker	*TaCKX6*-D1b		*TaCKX6*-D1a	18 bp deletion	-	
