# Supplementary material for: Analysis of main effect QTL for thousand grain weight in European winter wheat (Triticum aestivum L.) by genome-wide association mapping
Source: Front Plant Sci. 2015 Sep 1;6:644. doi: 10.3389/fpls.2015.00644 (PMC4555037; doi:10.3389/fpls.2015.00644)
Supplement: Supplementary file 1 [file DataSheet1.ZIP › Supplementary/152871_Röder_Data_Sheet_2.PDF]

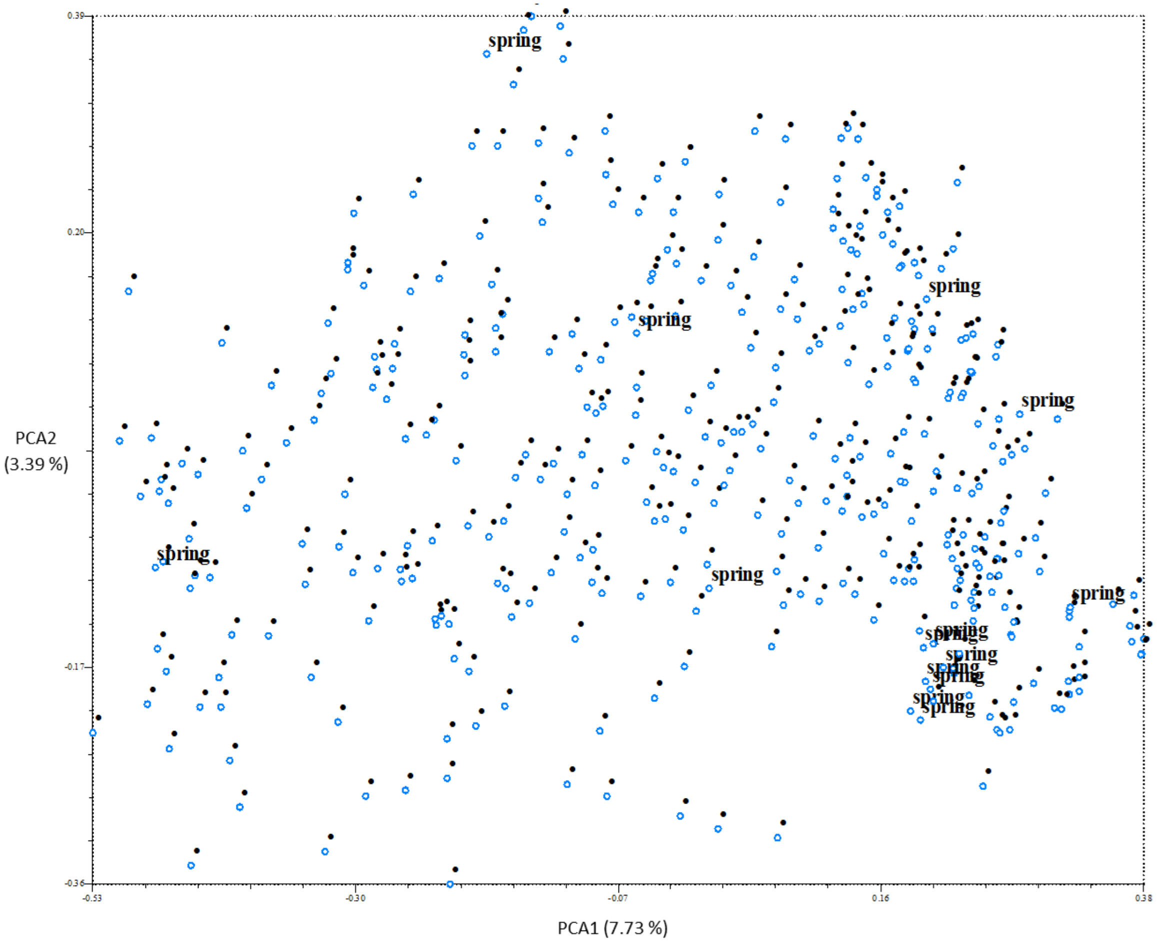

Supplemental file 2: Scatter plot of the 1st and 2nd principal components shows no apparent population structure among the 372 wheat varieties. The PCA analysis was conducted with 10,000 SNP-markers. The varieties were classified by the growth habit; ‘spring’ indicates the 14 spring varieties and black dots indicate the remaining 358 winter varieties. The variance explained by each principal component is given in the axis heading.
