# Supplementary material for: Analysis of main effect QTL for thousand grain weight in European winter wheat (Triticum aestivum L.) by genome-wide association mapping
Source: Front Plant Sci. 2015 Sep 1;6:644. doi: 10.3389/fpls.2015.00644 (PMC4555037; doi:10.3389/fpls.2015.00644)
Supplement: Supplementary file 1 [file DataSheet1.ZIP › Supplementary/152871_Röder_Data_Sheet_3.PDF]

**Supplemental file 3: Quantile-Quantile-plots in genome-wide association mapping with SSR- markers (A-C) and SNP-markers (D-F).**

The graphs plot the observed against the expected $-\log_{10}$ (P-value) of the TGW-BLUEs (A, D) and the two single environment TGW-09AND (B, E) and TGW-10JAN (C, F).

A

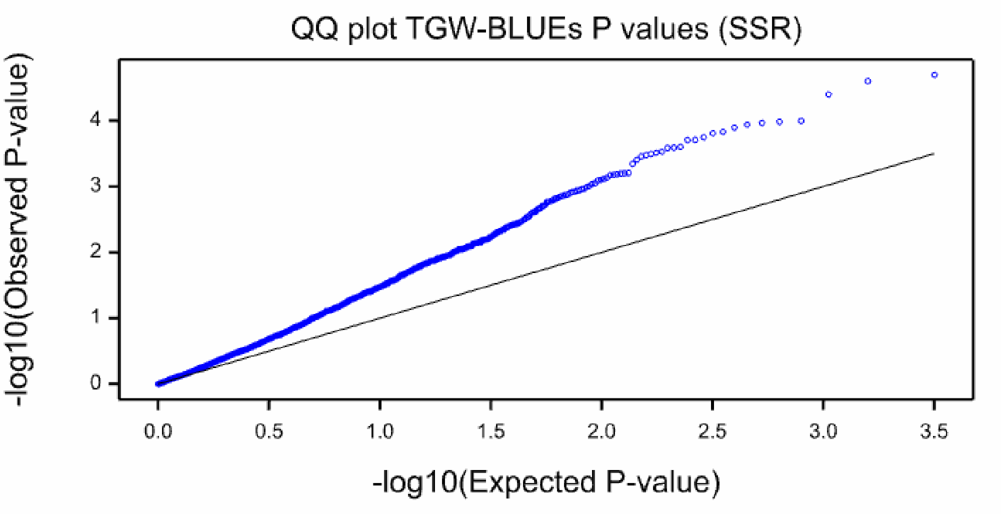


B

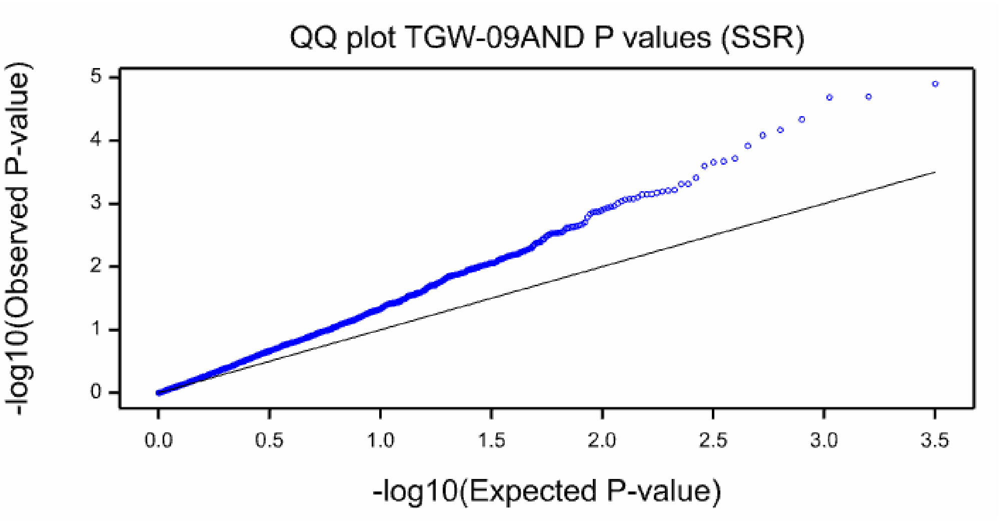


C

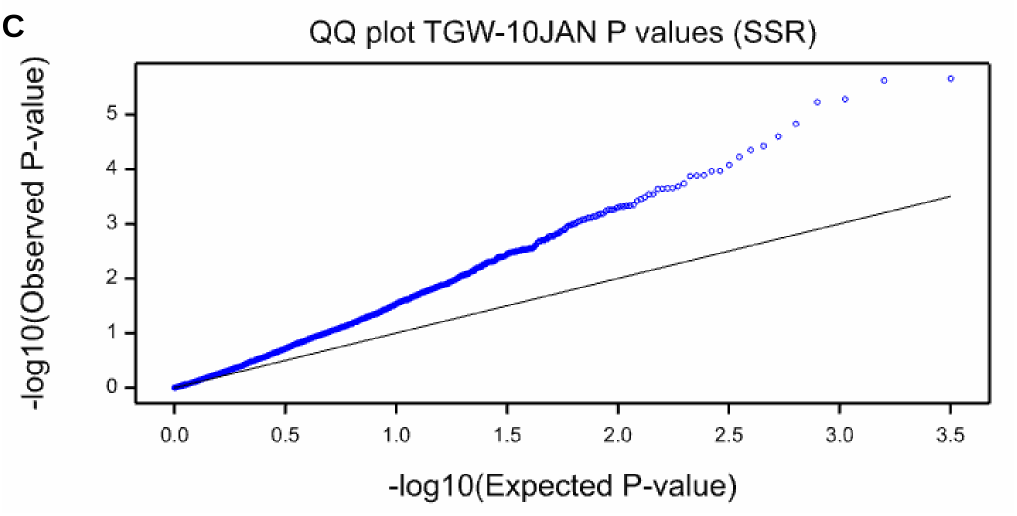

D

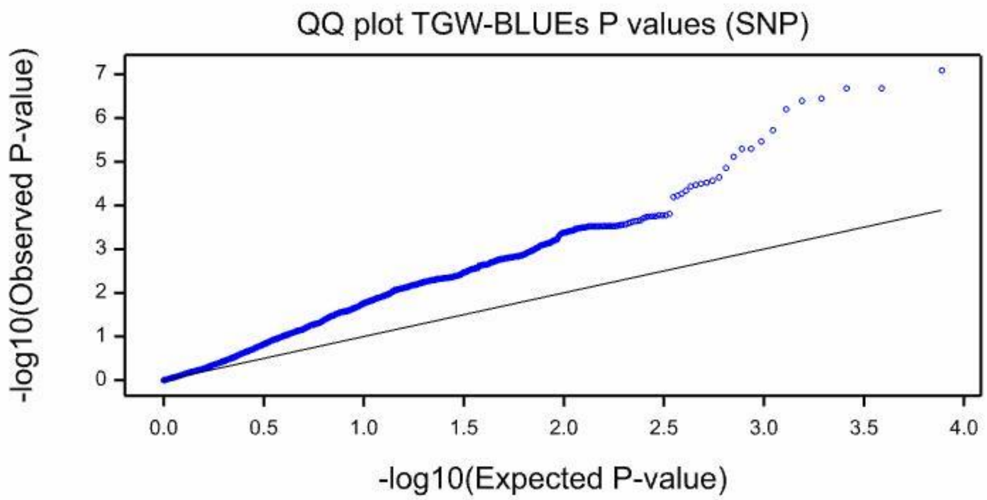
QQ plot TGW-BLUEs P values (SNP)
-log10(Observed P-value)
-log10(Expected P-value)
0
1
2
3
4
5
6
7
0.0
0.5
1.0
1.5
2.0
2.5
3.0
3.5
4.0

E

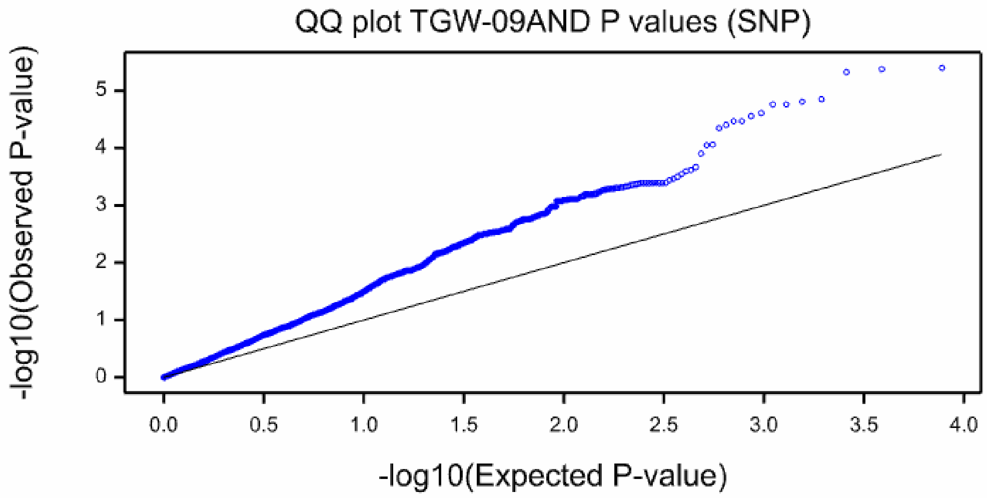
QQ plot TGW-09AND P values (SNP)
-log10(Observed P-value)
-log10(Expected P-value)
0
1
2
3
4
5
0.0
0.5
1.0
1.5
2.0
2.5
3.0
3.5
4.0

F

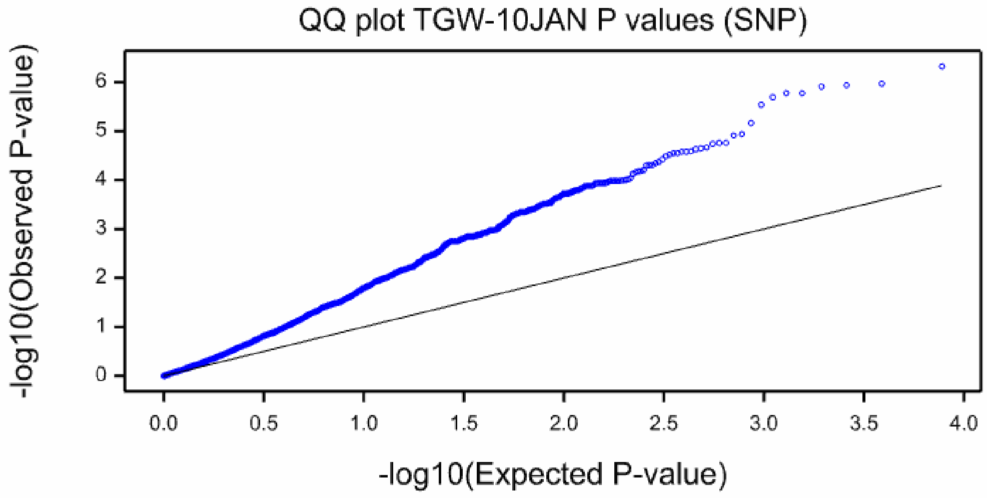
QQ plot TGW-10JAN P values (SNP)
-log10(Observed P-value)
-log10(Expected P-value)
0
1
2
3
4
5
6
0.0
0.5
1.0
1.5
2.0
2.5
3.0
3.5
4.0
