# Supplementary material for: Analysis of main effect QTL for thousand grain weight in European winter wheat (Triticum aestivum L.) by genome-wide association mapping
Source: Front Plant Sci. 2015 Sep 1;6:644. doi: 10.3389/fpls.2015.00644 (PMC4555037; doi:10.3389/fpls.2015.00644)
Supplement: Supplementary file 1 [file DataSheet1.ZIP › Supplementary/152871_Röder_Data_Sheet_4.PDF]

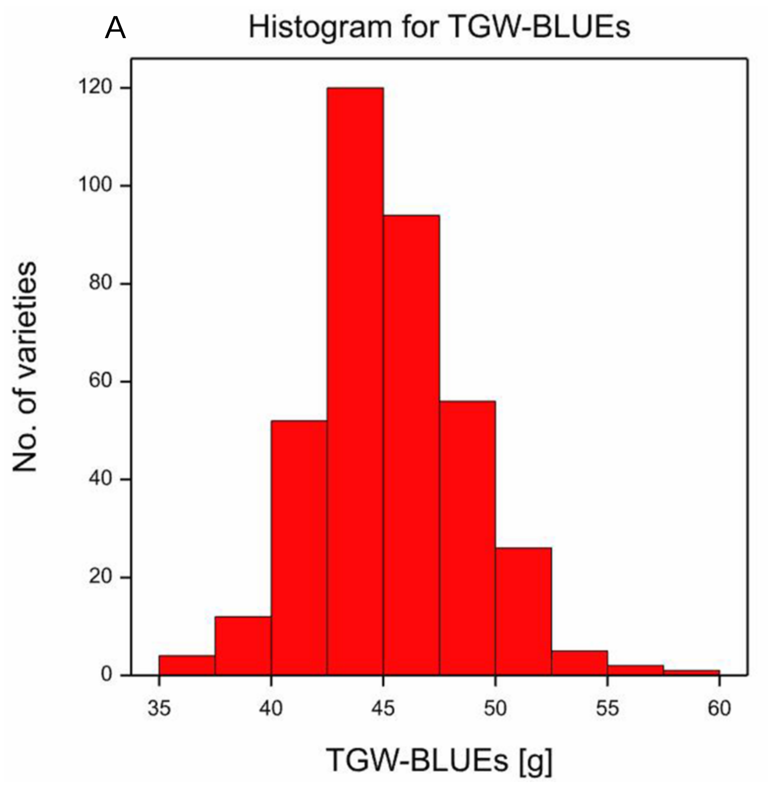

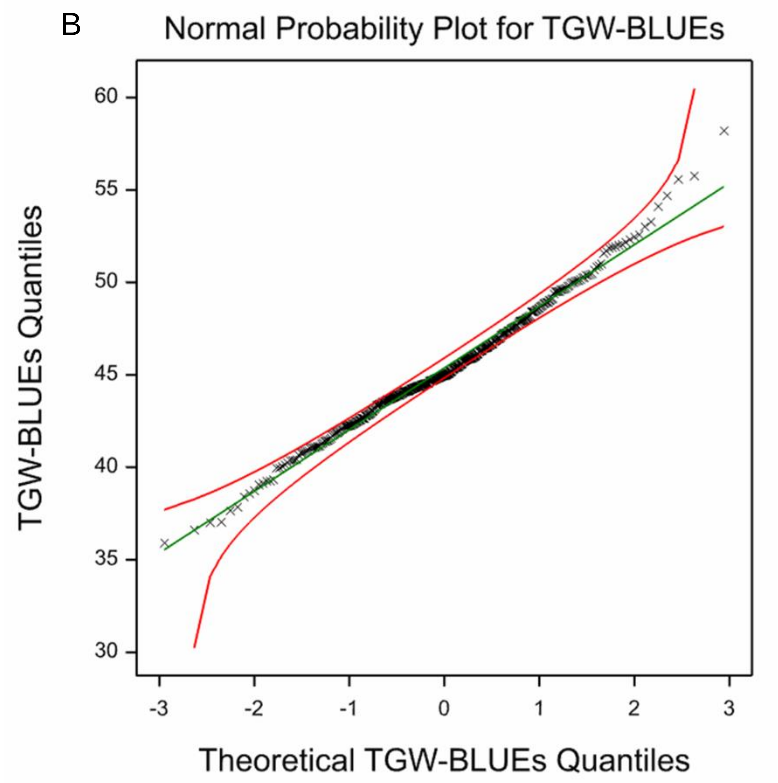

Supplemental file 5: Phenotypic distribution of TGW-BLUEs as histogram (A) and normal probability plot (B).

The distribution of the TGW-BLUEs within the 372 varieties showed a normal distribution (A) and a Normal QQ-plot within a 95% confidence interval (B).
