# Supplementary material for: Analysis of main effect QTL for thousand grain weight in European winter wheat (Triticum aestivum L.) by genome-wide association mapping
Source: Front Plant Sci. 2015 Sep 1;6:644. doi: 10.3389/fpls.2015.00644 (PMC4555037; doi:10.3389/fpls.2015.00644)
Supplement: Supplementary file 1 [file DataSheet1.ZIP › Supplementary/152871_Röder_Data_Sheet_5.PDF]

Supplemental file 6: Spearman rank order correlation of TGW-scores in 372 varieties among eight environments and the BLUEs.

	09SEL.TGW	09WOH.TGW	10AND.TGW	10JAN.TGW	10SAU.TGW	10SEL.TGW	10WOH.TGW	BLUES.TGW
**09AND.TGW**	0.773	0.767	0.676	0.710	0.680	0.755	0.753	0.882
**09SEL.TGW**		0.747	0.621	0.594	0.640	0.772	0.723	0.847
**09WOH.TGW**			0.680	0.765	0.723	0.772	0.742	0.898
**10AND.TGW**				0.690	0.704	0.694	0.734	0.824
**10JAN.TGW**					0.653	0.640	0.649	0.817
**10SAU.TGW**						0.704	0.752	0.833
**10SEL.TGW**							0.804	0.889
**10WOH.TGW**								0.886

P-value = 0.0000002
