# Supplementary material for: Analysis of main effect QTL for thousand grain weight in European winter wheat (Triticum aestivum L.) by genome-wide association mapping
Source: Front Plant Sci. 2015 Sep 1;6:644. doi: 10.3389/fpls.2015.00644 (PMC4555037; doi:10.3389/fpls.2015.00644)
Supplement: Supplementary file 1 [file DataSheet1.ZIP › Supplementary/152871_Röder_Data_Sheet_6.PDF]

Supplemental file 7: Analysis of variance (ANOVA) of TGW of 372 varieties in eight environments.

Source of Variation	DF	SS	MS	F	P
Genotype	371	33331.446	89.842	24	<0,001
Environment	7	16949.235	2421.319	656	<0,001
Residual	2597	9579.618	3.689		
Total	2975	59860.299	20.121		

DF = Degrees of freedom
SS = Sum of Squares
MS = Mean of Squares
***** P<0.001
