# Supplementary material for: Analysis of main effect QTL for thousand grain weight in European winter wheat (Triticum aestivum L.) by genome-wide association mapping
Source: Front Plant Sci. 2015 Sep 1;6:644. doi: 10.3389/fpls.2015.00644 (PMC4555037; doi:10.3389/fpls.2015.00644)
Supplement: Supplementary file 1 [file DataSheet1.ZIP › Supplementary/152871_Röder_Data_Sheet_7.PDF]

Supplemental file 8: Estimation of variance components and broad sense heritability and estimation of differences between groups (= environments) using ANOVA and a Tukey B test.

Variance Estimates

Component	Estimate
Var(Genotype)	9.957
Var(Error)	10.188

Dependent Variable: TGW
Method: Minimum Norm Quadratic Unbiased Estimation (Weight = 1 for Random Effects and Residual)

$H^2 = 0.887$

ANOVA

TGW

	Sum of Squares	df	Mean Square	F	Sig.
Between Groups	16949.235	7	2421.319	167.474	.000
Within Groups	42911.063	2968	14.458		
Total	59860.299	2975			

TGW Posthoc test

Tukey B[a]

env no	N	Subset for alpha = 0.05				
		1	2	3	4	5
10.JAN.TGW Mean	372	42.3330				
10.SAU.TGW Mean	372	42.3840				
09.WOH.TGW Mean	372		43.9568			
10.WOH.TGW Mean	372			45.5537		
10.SEL.TGW Mean	372			45.6992		
10.AND.TGW Mean	372			46.2518	46.2518	
09.AND.TGW Mean	372				46.6594	
090.SEL.TGW Mean	372					50.1495

Means for groups in homogeneous subsets are displayed.

a. Uses Harmonic Mean Sample Size = 372.000.
