# Supplementary material for: Analysis of main effect QTL for thousand grain weight in European winter wheat (Triticum aestivum L.) by genome-wide association mapping
Source: Front Plant Sci. 2015 Sep 1;6:644. doi: 10.3389/fpls.2015.00644 (PMC4555037; doi:10.3389/fpls.2015.00644)
Supplement: Supplementary file 1 [file DataSheet1.ZIP › Supplementary/152871_Röder_Data_Sheet_8.PDF]

**Supplemental file 12: Number of MTAs per chromosome for the SSR and SNP markers.**

| | Number of analyzed loci | | MTAs with LOD ≥3.0 | | MTAs with LOD ≥ 4.82 | MTAs with LOD≥ 5.89 |
|---|---|---|---|---|---|---|
| | SSR* | SNP* | SSR (BLUEs) | SNP (BLUEs) | SSR (BLUEs) | SNP (BLUEs) |
| Chr 1A | 22 | 506 | 2 (0) | 23 (0) | 0 | 1 (0) |
| Chr 1B | 33 | 676 | 26 (3) | 220 (19) | 1 (0) | 26 (0) |
| Chr 1D | 25 | 79 | 2 (1) | 4 (0) | 0 | 0 |
| Chr 2A | 32 | 461 | 3 (1) | 116 (2) | 0 | 0 |
| Chr 2B | 35 | 721 | 9 (1) | 48 (4) | 2 (0) | 0 |
| Chr 2D | 36 | 96 | 22 (3) | 19 (5) | 2 (0) | 0 |
| Chr 3A | 32 | 470 | 12 (2) | 38 (1) | 1 (0) | 0 |
| Chr 3B | 39 | 764 | 26 (4) | 296 (41) | 2 (0) | 23 (6) |
| Chr 3D | 39 | 52 | 29 (4) | 6 (0) | 2 (0) | 0 |
| Chr 4A | 32 | 235 | 15 (1) | 41 (5) | 3 (0) | 0 |
| Chr 4B | 20 | 339 | 0 | 39 (6) | 0 | 0 |
| Chr 4D | 18 | 12 | 1 (0) | 0 | 0 | 0 |
| Chr 5A | 25 | 456 | 11 (1) | 89 (12) | 0 | 4 (1) |
| Chr 5B | 33 | 759 | 12 (1) | 81 (4) | 0 | 0 |
| Chr 5D | 48 | 91 | 15 (2) | 0 | 0 | 0 |
| Chr 6A | 17 | 535 | 7 (0) | 17 (0) | 0 | 0 |
| Chr 6B | 18 | 364 | 0 | 1 (0) | 0 | 0 |
| Chr 6D | 30 | 49 | 40 (6) | 10 (2) | 6 (1) | 0 |
| Chr 7A | 31 | 554 | 17 (1) | 72 (6) | 2 (0) | 0 |
| Chr 7B | 24 | 481 | 15 (2) | 59 (5) | 2 (0) | 0 |
| Chr 7D | 46 | 69 | 10 (1) | 16 (3) | 0 | 4 (0) |
| unm. | 135 | 0 | 68 (9) | 0 | 5 (0) | 0 |
| **SUM** | **770** | **7769** | **342 (43)** | **1195 (115)** | **28 (1)** | **58 (7)** |

* No. of loci used for analysis
