# Supplementary material for: Analysis of main effect QTL for thousand grain weight in European winter wheat (Triticum aestivum L.) by genome-wide association mapping
Source: Front Plant Sci. 2015 Sep 1;6:644. doi: 10.3389/fpls.2015.00644 (PMC4555037; doi:10.3389/fpls.2015.00644)
Supplement: Supplementary file 1 [file DataSheet1.ZIP › Supplementary/152871_Röder_Image_1.PDF]

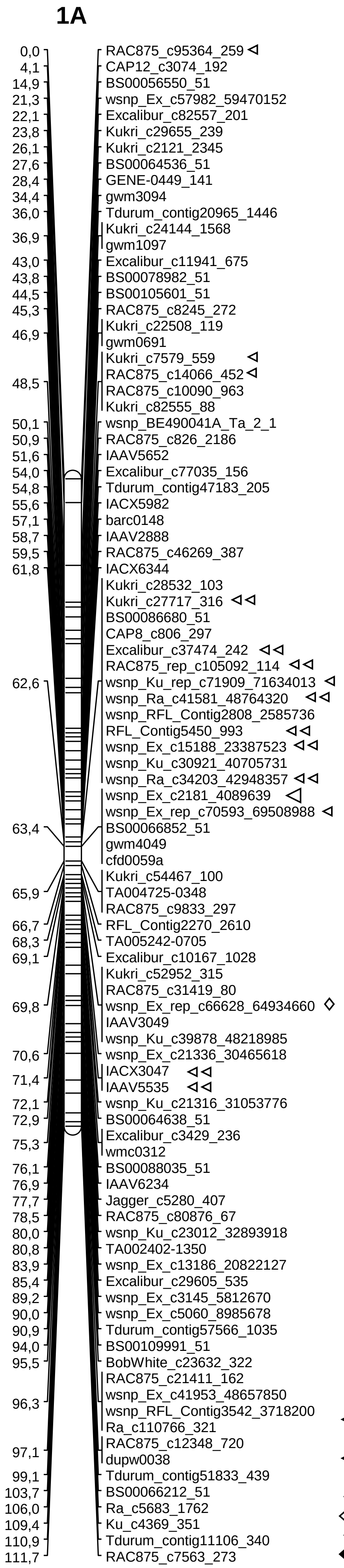

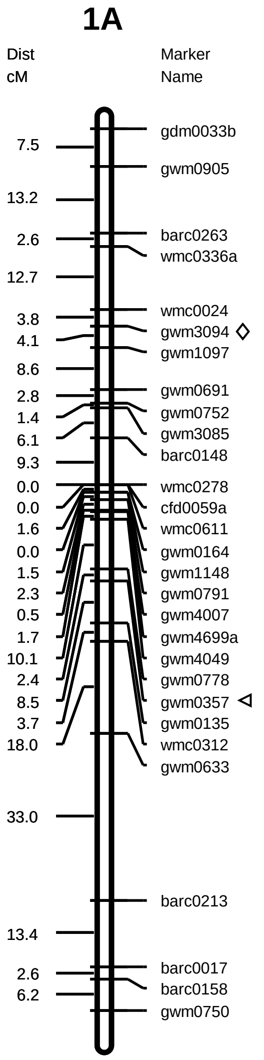

Supplemental file 13: Chromosomal location of marker trait associations.

◁ Thousand grain weight decreasing effect, single environment, $-\log_{10}(p)$-value ≥ 3.0
◁ Thousand grain weight decreasing effect, single environment, $-\log_{10}(p)$-value ≥ 4.82 (SSR) ≥ 5.89 (SNP)
◀ Thousand grain weight decreasing effect, BLUEs, $-\log_{10}(p)$-value ≥ 3.0
◀ Thousand grain weight decreasing effect, BLUEs, $-\log_{10}(p)$-value ≥ 4.82 (SSR) ≥ 5.89 (SNP)

◇ Thousand grain weight increasing effect, single environment, $-\log_{10}(p)$-value ≥ 3.0
◇ Thousand grain weight increasing effect, single environment, $-\log_{10}(p)$-value ≥ 4.82 (SSR) ≥ 5.89 (SNP)
◆ Thousand grain weight increasing effect, BLUEs, $-\log_{10}(p)$-value ≥ 3.0
◆ Thousand grain weight increasing effect, BLUEs, $-\log_{10}(p)$-value ≥ 4.82 (SSR) ≥ 5.89 (SNP)

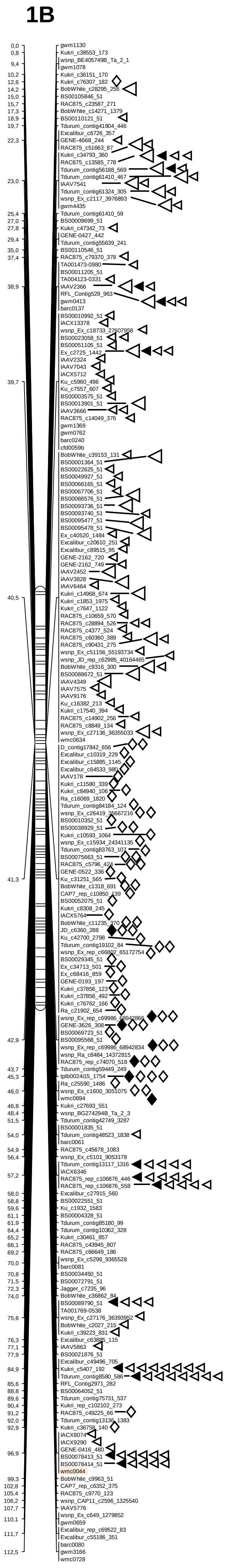

1B

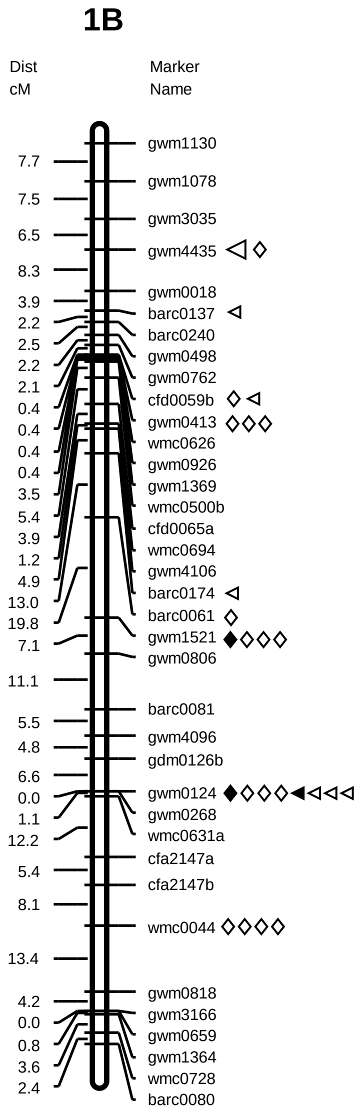

1B
Dist cM
Marker Name
7.7
7.5
6.5
8.3
3.9
2.2
2.5
2.2
2.1
0.4
0.4
0.4
0.4
3.5
5.4
3.9
1.2
4.9
13.0
19.8
7.1
11.1
5.5
4.8
6.6
0.0
1.1
12.2
5.4
8.1
13.4
4.2
0.0
0.8
3.6
2.4
gwm1130
gwm1078
gwm3035
gwm4435
gwm0018
barc0137
barc0240
gwm0498
gwm0762
cfd0059b
gwm0413
wmc0626
gwm0926
gwm1369
wmc0500b
cfd0065a
wmc0694
gwm4106
barc0174
barc0061
gwm1521
gwm0806
barc0081
gwm4096
gdm0126b
gwm0124
gwm0268
wmc0631a
cfa2147a
cfa2147b
wmc0044
gwm0818
gwm3166
gwm0659
gwm1364
wmc0728
barc0080

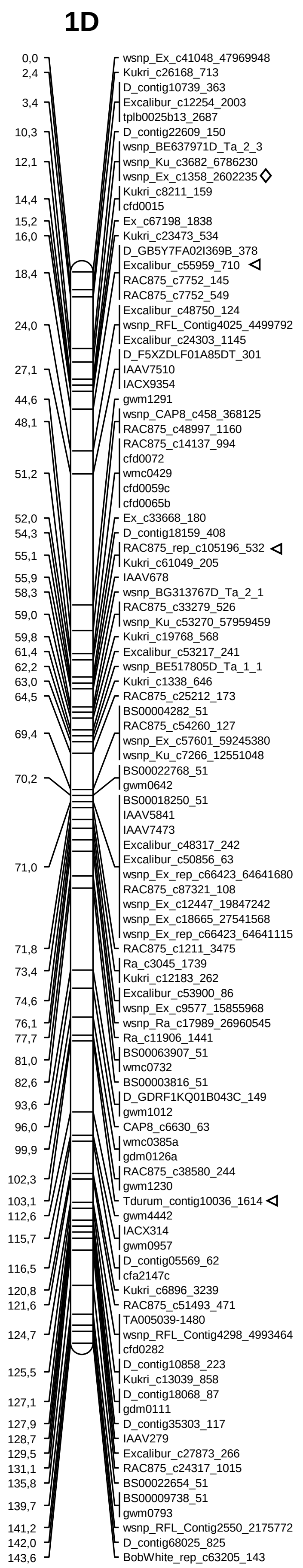
1D
0,0
2,4
3,4
10,3
12,1
14,4
15,2
16,0
18,4
24,0
27,1
44,6
48,1
51,2
52,0
54,3
55,1
55,9
58,3
59,0
59,8
61,4
62,2
63,0
64,5
69,4
70,2
71,0
71,8
73,4
74,6
76,1
77,7
81,0
82,6
93,6
96,0
99,9
102,3
103,1
112,6
115,7
116,5
120,8
121,6
124,7
125,5
127,1
127,9
128,7
129,5
131,1
135,8
139,7
141,2
142,0
143,6
wsnp_Ex_c41048_47969948
Kukri_c26168_713
D_contig10739_363
Excalibur_c12254_2003
tplb0025b13_2687
D_contig22609_150
wsnp_BE637971D_Ta_2_3
wsnp_Ku_c3682_6786230
wsnp_Ex_c1358_2602235
Kukri_c8211_159
cfd0015
Ex_c67198_1838
Kukri_c23473_534
D_GB5Y7FA02I369B_378
Excalibur_c55959_710
RAC875_c7752_145
RAC875_c7752_549
Excalibur_c48750_124
wsnp_RFL_Contig4025_4499792
Excalibur_c24303_1145
D_F5XZDLF01A85DT_301
IAAV7510
IACX9354
gwm1291
wsnp_CAP8_c458_368125
RAC875_c48997_1160
RAC875_c14137_994
cfd0072
wmc0429
cfd0059c
cfd0065b
Ex_c33668_180
D_contig18159_408
RAC875_rep_c105196_532
Kukri_c61049_205
IAAV678
wsnp_BG313767D_Ta_2_1
RAC875_c33279_526
wsnp_Ku_c53270_57959459
Kukri_c19768_568
Excalibur_c53217_241
wsnp_BE517805D_Ta_1_1
Kukri_c1338_646
RAC875_c25212_173
BS00004282_51
RAC875_c54260_127
wsnp_Ex_c57601_59245380
wsnp_Ku_c7266_12551048
BS00022768_51
gwm0642
BS00018250_51
IAAV5841
IAAV7473
Excalibur_c48317_242
Excalibur_c50856_63
wsnp_Ex_rep_c66423_64641680
RAC875_c87321_108
wsnp_Ex_c12447_19847242
wsnp_Ex_c18665_27541568
wsnp_Ex_rep_c66423_64641115
RAC875_c1211_3475
Ra_c3045_1739
Kukri_c12183_262
Excalibur_c53900_86
wsnp_Ex_c9577_15855968
wsnp_Ra_c17989_26960545
Ra_c11906_1441
BS00063907_51
wmc0732
BS00003816_51
D_GDRF1KQ01B043C_149
gwm1012
CAP8_c6630_63
wmc0385a
gdm0126a
RAC875_c38580_244
gwm1230
Tdurum_contig10036_1614
gwm4442
IACX314
gwm0957
D_contig05569_62
cfa2147c
Kukri_c6896_3239
RAC875_c51493_471
TA005039-1480
wsnp_RFL_Contig4298_4993464
cfd0282
D_contig10858_223
Kukri_c13039_858
D_contig18068_87
gdm0111
D_contig35303_117
IAAV279
Excalibur_c27873_266
RAC875_c24317_1015
BS00022654_51
BS00009738_51
gwm0793
wsnp_RFL_Contig2550_2175772
D_contig68025_825
BobWhite_rep_c63205_143

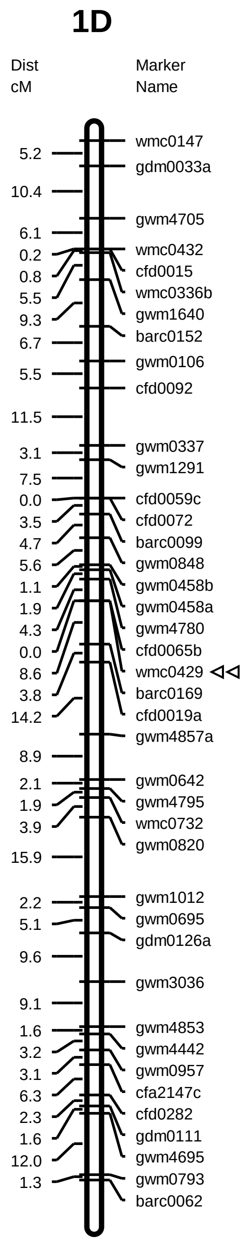
1D
Dist cM
Marker Name
5.2
10.4
6.1
0.2
0.8
5.5
9.3
6.7
5.5
11.5
3.1
7.5
0.0
3.5
4.7
5.6
1.1
1.9
4.3
0.0
8.6
3.8
14.2
8.9
2.1
1.9
3.9
15.9
2.2
5.1
9.6
9.1
1.6
3.2
3.1
6.3
2.3
1.6
12.0
1.3
wmc0147
gdm0033a
gwm4705
wmc0432
cfd0015
wmc0336b
gwm1640
barc0152
gwm0106
cfd0092
gwm0337
gwm1291
cfd0059c
cfd0072
barc0099
gwm0848
gwm0458b
gwm0458a
gwm4780
cfd0065b
wmc0429
barc0169
cfd0019a
gwm4857a
gwm0642
gwm4795
wmc0732
gwm0820
gwm1012
gwm0695
gdm0126a
gwm3036
gwm4853
gwm4442
gwm0957
cfa2147c
cfd0282
gdm0111
gwm4695
gwm0793
barc0062

2A

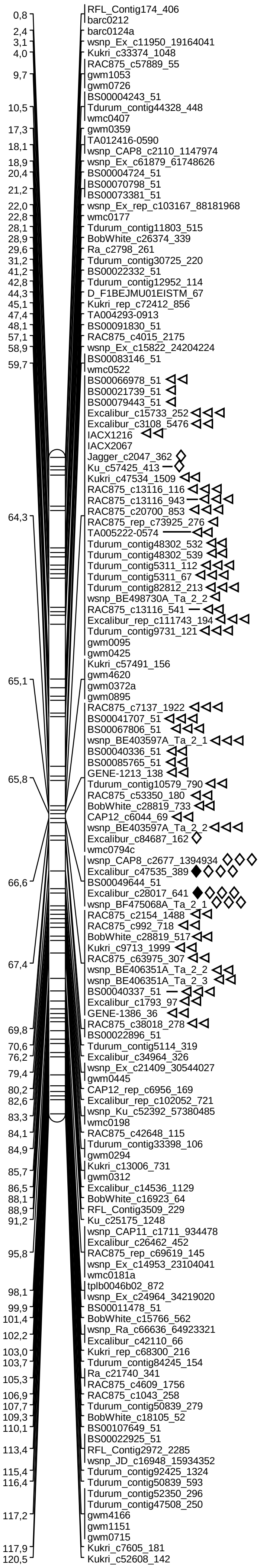
0,8
2,4
3,1
4,0
9,7
10,5
17,3
18,1
18,9
20,4
21,2
22,0
22,8
28,1
28,9
29,6
31,2
41,2
42,8
44,3
45,1
47,4
48,1
57,1
58,9
59,7
64,3
65,1
65,8
66,6
67,4
69,8
70,6
76,2
79,4
80,2
82,6
83,3
84,1
84,9
85,7
86,5
88,1
88,9
91,2
95,8
98,1
99,9
101,4
102,2
103,0
103,7
105,3
106,9
107,7
109,3
110,1
113,4
115,4
116,4
117,2
117,9
120,5
RFL_Contig174_406
barc0212
barc0124a
wsnp_Ex_c11950_19164041
Kukri_c33374_1048
RAC875_c57889_55
gwm1053
gwm0726
BS00004243_51
Tdurum_contig44328_448
wmc0407
gwm0359
TA012416-0590
wsnp_CAP8_c2110_1147974
wsnp_Ex_c61879_61748626
BS00004724_51
BS00070798_51
BS00073381_51
wsnp_Ex_rep_c103167_88181968
wmc0177
Tdurum_contig11803_515
BobWhite_c26374_339
Ra_c2798_261
Tdurum_contig30725_220
BS00022332_51
Tdurum_contig12952_114
D_F1BEJMU01EISTM_67
Kukri_rep_c72412_856
TA004293-0913
BS00091830_51
RAC875_c4015_2175
wsnp_Ex_c15822_24204224
BS00083146_51
wmc0522
BS00066978_51
BS00021739_51
BS00079443_51
Excalibur_c15733_252
Excalibur_c3108_5476
IACX1216
IACX2067
Jagger_c2047_362
Ku_c57425_413
Kukri_c47534_1509
RAC875_c13116_116
RAC875_c13116_943
RAC875_c20700_853
RAC875_rep_c73925_276
TA005222-0574
Tdurum_contig48302_532
Tdurum_contig48302_539
Tdurum_contig5311_112
Tdurum_contig5311_67
Tdurum_contig82812_213
wsnp_BE498730A_Ta_2_2
RAC875_c13116_541
Excalibur_rep_c111743_194
Tdurum_contig9731_121
gwm0095
gwm0425
Kukri_c57491_156
gwm4620
gwm0372a
gwm0895
RAC875_c7137_1922
BS00041707_51
BS00067806_51
wsnp_BE403597A_Ta_2_1
BS00040336_51
BS00085765_51
GENE-1213_138
Tdurum_contig10579_790
RAC875_c53350_180
BobWhite_c28819_733
CAP12_c6044_69
wsnp_BE403597A_Ta_2_2
Excalibur_c84687_162
wmc0794c
wsnp_CAP8_c2677_1394934
Excalibur_c47535_389
BS00049644_51
Excalibur_c28017_641
wsnp_BF475068A_Ta_2_1
RAC875_c2154_1488
RAC875_c992_718
BobWhite_c28819_517
Kukri_c9713_1999
RAC875_c63975_307
wsnp_BE406351A_Ta_2_2
wsnp_BE406351A_Ta_2_3
BS00040337_51
Excalibur_c1793_97
GENE-1386_36
RAC875_c38018_278
BS00022896_51
Tdurum_contig5114_319
Excalibur_c34964_326
wsnp_Ex_c21409_30544027
gwm0445
CAP12_rep_c6956_169
Excalibur_rep_c102052_721
wsnp_Ku_c52392_57380485
wmc0198
RAC875_c42648_115
Tdurum_contig33398_106
gwm0294
Kukri_c13006_731
gwm0312
Excalibur_c14536_1129
BobWhite_c16923_64
RFL_Contig3509_229
Ku_c25175_1248
wsnp_CAP11_c1711_934478
Excalibur_c26462_452
RAC875_rep_c69619_145
wsnp_Ex_c14953_23104041
wmc0181a
tplb0046b02_872
wsnp_Ex_c24964_34219020
BS00011478_51
BobWhite_c15766_562
wsnp_Ra_c66636_64923321
Excalibur_c42110_66
Kukri_rep_c68300_216
Tdurum_contig84245_154
Ra_c21740_341
RAC875_c4609_1756
RAC875_c1043_258
Tdurum_contig50839_279
BobWhite_c18105_52
BS00107649_51
BS00022925_51
RFL_Contig2972_2285
wsnp_JD_c16948_15934352
Tdurum_contig92425_1324
Tdurum_contig50839_593
Tdurum_contig52350_296
Tdurum_contig47508_250
gwm4166
gwm1151
gwm0715
Kukri_c7605_181
Kukri_c52608_142

2A

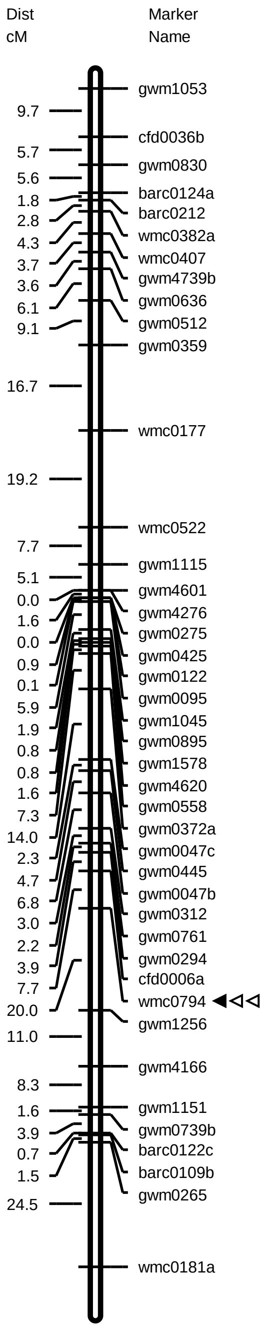
Dist
cM
Marker
Name
9.7
5.7
5.6
1.8
2.8
4.3
3.7
3.6
6.1
9.1
16.7
19.2
7.7
5.1
0.0
1.6
0.0
0.9
0.1
5.9
1.9
0.8
0.8
1.6
7.3
14.0
2.3
4.7
6.8
3.0
2.2
3.9
7.7
20.0
11.0
8.3
1.6
3.9
0.7
1.5
24.5
gwm1053
cfd0036b
gwm0830
barc0124a
barc0212
wmc0382a
wmc0407
gwm4739b
gwm0636
gwm0512
gwm0359
wmc0177
wmc0522
gwm1115
gwm4601
gwm4276
gwm0275
gwm0425
gwm0122
gwm0095
gwm1045
gwm0895
gwm1578
gwm4620
gwm0558
gwm0372a
gwm0047c
gwm0445
gwm0047b
gwm0312
gwm0761
gwm0294
cfd0006a
wmc0794
gwm1256
gwm4166
gwm1151
gwm0739b
barc0122c
barc0109b
gwm0265
wmc0181a

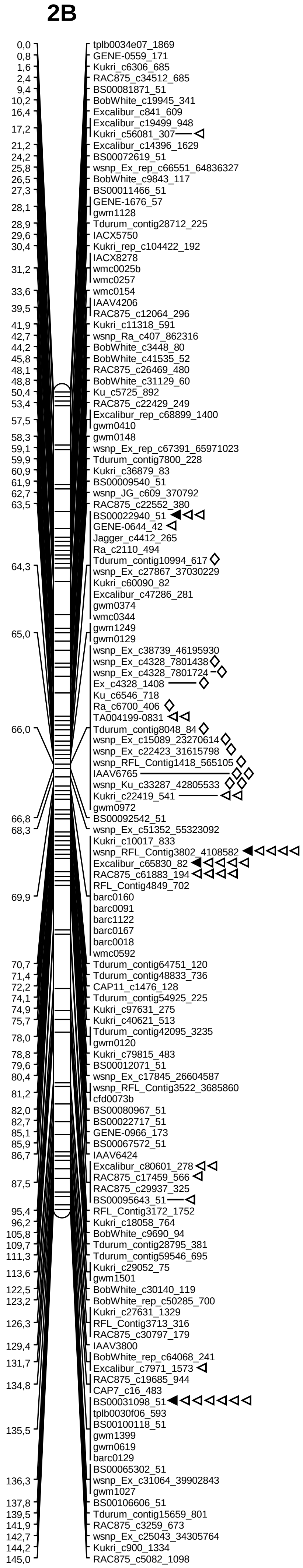
2B
0,0
0,8
1,6
2,4
9,4
10,2
16,4
17,2
21,2
24,2
25,8
26,5
27,3
28,1
28,9
29,6
30,4
31,2
33,6
39,5
41,9
42,7
44,2
45,8
48,1
48,8
50,4
53,4
57,5
58,3
59,1
59,9
60,9
61,9
62,7
63,5
64,3
65,0
66,0
66,8
68,3
69,9
70,7
71,4
72,2
74,1
74,9
75,7
78,0
78,8
79,6
80,4
81,2
82,0
82,7
85,1
85,9
86,7
87,5
95,4
96,2
105,8
109,7
111,3
113,6
122,5
123,2
126,3
129,4
131,7
134,8
135,5
136,3
137,8
139,5
141,9
142,7
144,2
145,0
tplb0034e07_1869
GENE-0559_171
Kukri_c6306_685
RAC875_c34512_685
BS00081871_51
BobWhite_c19945_341
Excalibur_c841_609
Excalibur_c19499_948
Kukri_c56081_307
Excalibur_c14396_1629
BS00072619_51
wsnp_Ex_rep_c66551_64836327
BobWhite_c9843_117
BS00011466_51
GENE-1676_57
gwm1128
Tdurum_contig28712_225
IACX5750
Kukri_rep_c104422_192
IACX8278
wmc0025b
wmc0257
wmc0154
IAAV4206
RAC875_c12064_296
Kukri_c11318_591
wsnp_Ra_c407_862316
BobWhite_c3448_80
BobWhite_c41535_52
RAC875_c26469_480
BobWhite_c31129_60
Ku_c5725_892
RAC875_c22429_249
Excalibur_rep_c68899_1400
gwm0410
gwm0148
wsnp_Ex_rep_c67391_65971023
Tdurum_contig7800_228
Kukri_c36879_83
BS00009540_51
wsnp_JG_c609_370792
RAC875_c22552_380
BS00022940_51
GENE-0644_42
Jagger_c4412_265
Ra_c2110_494
Tdurum_contig10994_617
wsnp_Ex_c27867_37030229
Kukri_c60090_82
Excalibur_c47286_281
gwm0374
wmc0344
gwm1249
gwm0129
wsnp_Ex_c38739_46195930
wsnp_Ex_c4328_7801438
wsnp_Ex_c4328_7801724
Ex_c4328_1408
Ku_c6546_718
Ra_c6700_406
TA004199-0831
Tdurum_contig8048_84
wsnp_Ex_c15089_23270614
wsnp_Ex_c22423_31615798
wsnp_RFL_Contig1418_565105
IAAV6765
wsnp_Ku_c33287_42805533
Kukri_c22419_541
gwm0972
BS00092542_51
wsnp_Ex_c51352_55323092
Kukri_c10017_833
wsnp_RFL_Contig3802_4108582
Excalibur_c65830_82
RAC875_c61883_194
RFL_Contig4849_702
barc0160
barc0091
barc1122
barc0167
barc0018
wmc0592
Tdurum_contig64751_120
Tdurum_contig48833_736
CAP11_c1476_128
Tdurum_contig54925_225
Kukri_c97631_275
Kukri_c40621_513
Tdurum_contig42095_3235
gwm0120
Kukri_c79815_483
BS00012071_51
wsnp_Ex_c17845_26604587
wsnp_RFL_Contig3522_3685860
cfd0073b
BS00080967_51
BS00022717_51
GENE-0966_173
BS00067572_51
IAAV6424
Excalibur_c80601_278
RAC875_c17459_566
RAC875_c29937_325
BS00095643_51
RFL_Contig3172_1752
Kukri_c18058_764
BobWhite_c9690_94
Tdurum_contig28795_381
Tdurum_contig59546_695
Kukri_c29052_75
gwm1501
BobWhite_c30140_119
BobWhite_rep_c50285_700
Kukri_c27631_1329
RFL_Contig3713_316
RAC875_c30797_179
IAAV3800
BobWhite_rep_c64068_241
Excalibur_c7971_1573
RAC875_c19685_944
CAP7_c16_483
BS00031098_51
tplb0030f06_593
BS00100118_51
gwm1399
gwm0619
barc0129
BS00065302_51
wsnp_Ex_c31064_39902843
gwm1027
BS00106606_51
Tdurum_contig15659_801
RAC875_c3259_673
wsnp_Ex_c25043_34305764
Kukri_c900_1334
RAC875_c5082_1098

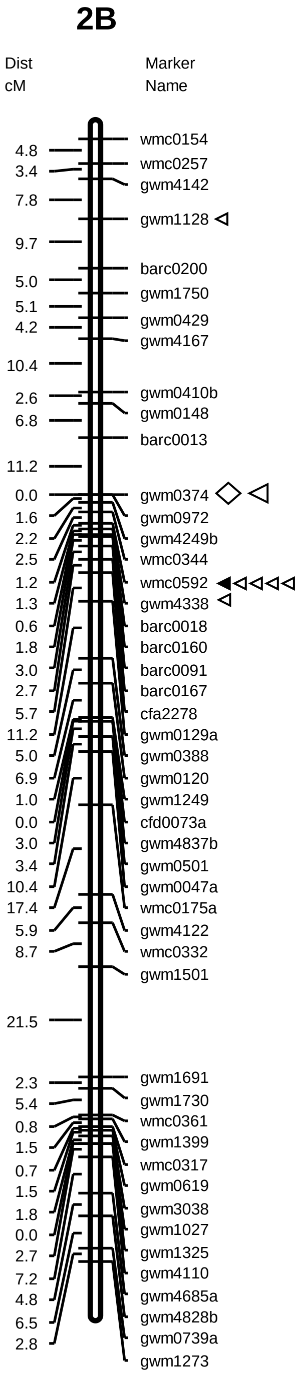
2B
Dist
cM
Marker
Name
4.8
3.4
7.8
9.7
5.0
5.1
4.2
10.4
2.6
6.8
11.2
0.0
1.6
2.2
2.5
1.2
1.3
0.6
1.8
3.0
2.7
5.7
11.2
5.0
6.9
1.0
0.0
3.0
3.4
10.4
17.4
5.9
8.7
21.5
2.3
5.4
0.8
1.5
0.7
1.5
1.8
0.0
2.7
7.2
4.8
6.5
2.8
wmc0154
wmc0257
gwm4142
gwm1128
barc0200
gwm1750
gwm0429
gwm4167
gwm0410b
gwm0148
barc0013
gwm0374
gwm0972
gwm4249b
wmc0344
wmc0592
gwm4338
barc0018
barc0160
barc0091
barc0167
cfa2278
gwm0129a
gwm0388
gwm0120
gwm1249
cfd0073a
gwm4837b
gwm0501
gwm0047a
wmc0175a
gwm4122
wmc0332
gwm1501
gwm1691
gwm1730
wmc0361
gwm1399
wmc0317
gwm0619
gwm3038
gwm1027
gwm1325
gwm4110
gwm4685a
gwm4828b
gwm0739a
gwm1273

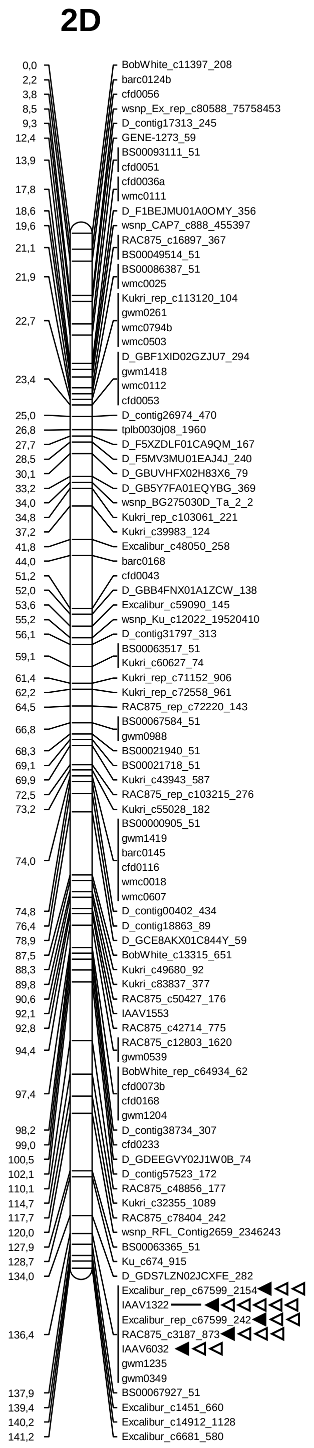
2D
0,0 BobWhite_c11397_208
2,2 barc0124b
3,8 cfd0056
8,5 wsnp_Ex_rep_c80588_75758453
9,3 D_contig17313_245
12,4 GENE-1273_59
13,9 BS00093111_51
cfd0051
17,8 cfd0036a
wmc0111
18,6 D_F1BEJMU01A0OMY_356
19,6 wsnp_CAP7_c888_455397
21,1 RAC875_c16897_367
BS00049514_51
21,9 BS00086387_51
wmc0025
22,7 Kukri_rep_c113120_104
gwm0261
wmc0794b
wmc0503
23,4 D_GBF1XID02GZJU7_294
gwm1418
wmc0112
cfd0053
25,0 D_contig26974_470
26,8 tplb0030j08_1960
27,7 D_F5XZDLF01CA9QM_167
28,5 D_F5MV3MU01EAJ4J_240
30,1 D_GBUVHFX02H83X6_79
33,2 D_GB5Y7FA01EQYBG_369
34,0 wsnp_BG275030D_Ta_2_2
34,8 Kukri_rep_c103061_221
37,2 Kukri_c39983_124
41,8 Excalibur_c48050_258
44,0 barc0168
51,2 cfd0043
52,0 D_GBB4FNX01A1ZCW_138
53,6 Excalibur_c59090_145
55,2 wsnp_Ku_c12022_19520410
56,1 D_contig31797_313
59,1 BS00063517_51
Kukri_c60627_74
61,4 Kukri_rep_c71152_906
62,2 Kukri_rep_c72558_961
64,5 RAC875_rep_c72220_143
66,8 BS00067584_51
gwm0988
68,3 BS00021940_51
69,1 BS00021718_51
69,9 Kukri_c43943_587
72,5 RAC875_rep_c103215_276
73,2 Kukri_c55028_182
74,0 BS00000905_51
gwm1419
barc0145
cfd0116
wmc0018
wmc0607
74,8 D_contig00402_434
76,4 D_contig18863_89
78,9 D_GCE8AKX01C844Y_59
87,5 BobWhite_c13315_651
88,3 Kukri_c49680_92
89,8 Kukri_c83837_377
90,6 RAC875_c50427_176
92,1 IAAV1553
92,8 RAC875_c42714_775
94,4 RAC875_c12803_1620
gwm0539
97,4 BobWhite_rep_c64934_62
cfd0073b
cfd0168
gwm1204
98,2 D_contig38734_307
99,0 cfd0233
100,5 D_GDEEGVY02J1W0B_74
102,1 D_contig57523_172
110,1 RAC875_c48856_177
114,7 Kukri_c32355_1089
117,7 RAC875_c78404_242
120,0 wsnp_RFL_Contig2659_2346243
127,9 BS00063365_51
128,7 Ku_c674_915
134,0 D_GDS7LZN02JCXFE_282
136,4 Excalibur_rep_c67599_2154
IAAV1322
Excalibur_rep_c67599_242
RAC875_c3187_873
IAAV6032
gwm1235
gwm0349
137,9 BS00067927_51
139,4 Excalibur_c1451_660
140,2 Excalibur_c14912_1128
141,2 Excalibur_c6681_580

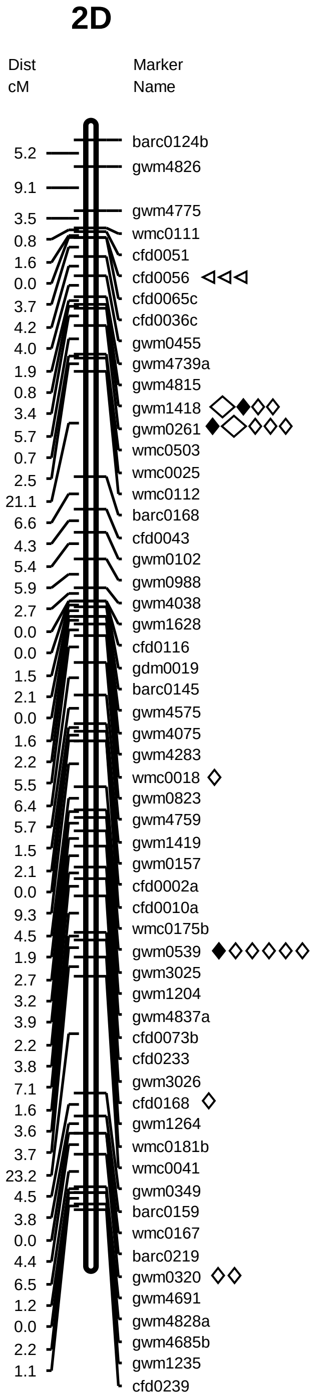
2D
Dist cM
Marker Name
5.2
9.1
3.5
0.8
1.6
0.0
3.7
4.2
4.0
1.9
0.8
3.4
5.7
0.7
2.5
21.1
6.6
4.3
5.4
5.9
2.7
0.0
0.0
1.5
2.1
0.0
1.6
2.2
5.5
6.4
5.7
1.5
2.1
0.0
9.3
4.5
1.9
2.7
3.2
3.9
2.2
3.8
7.1
1.6
3.6
3.7
23.2
4.5
3.8
0.0
4.4
6.5
1.2
0.0
2.2
1.1
barc0124b
gwm4826
gwm4775
wmc0111
cfd0051
cfd0056
cfd0065c
cfd0036c
gwm0455
gwm4739a
gwm4815
gwm1418
gwm0261
wmc0503
wmc0025
wmc0112
barc0168
cfd0043
gwm0102
gwm0988
gwm4038
gwm1628
cfd0116
gdm0019
barc0145
gwm4575
gwm4075
gwm4283
wmc0018
gwm0823
gwm4759
gwm1419
gwm0157
cfd0002a
cfd0010a
wmc0175b
gwm0539
gwm3025
gwm1204
gwm4837a
cfd0073b
cfd0233
gwm3026
cfd0168
gwm1264
wmc0181b
wmc0041
gwm0349
barc0159
wmc0167
barc0219
gwm0320
gwm4691
gwm4828a
gwm4685b
gwm1235
cfd0239

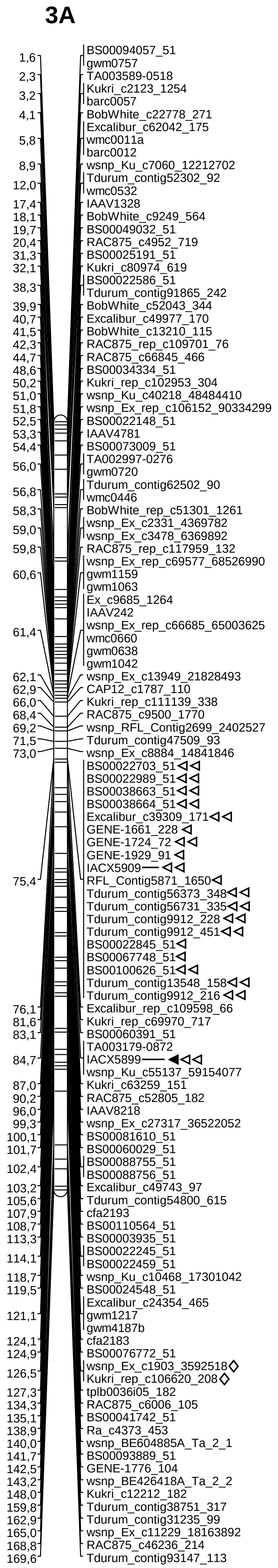
3A
1,6
2,3
3,2
4,1
5,8
8,9
12,0
17,4
18,1
19,7
20,4
31,3
32,1
38,3
39,9
40,7
41,5
42,3
44,7
48,6
50,2
51,0
51,8
52,5
53,3
54,4
56,0
56,8
58,3
59,0
59,8
60,6
61,4
62,1
62,9
66,0
68,4
69,2
71,5
73,0
75,4
76,1
81,6
83,1
84,7
87,0
90,2
96,0
99,3
100,1
101,7
102,4
103,2
105,6
107,9
108,7
113,3
114,1
118,7
119,5
121,1
124,1
124,9
126,5
127,3
134,3
135,1
138,9
140,0
141,7
142,5
143,2
148,0
159,8
162,9
165,0
168,8
169,6
BS00094057_51
gwm0757
TA003589-0518
Kukri_c2123_1254
barc0057
BobWhite_c22778_271
Excalibur_c62042_175
wmc0011a
barc0012
wsnp_Ku_c7060_12212702
Tdurum_contig52302_92
wmc0532
IAAV1328
BobWhite_c9249_564
BS00049032_51
RAC875_c4952_719
BS00025191_51
Kukri_c80974_619
BS00022586_51
Tdurum_contig91865_242
BobWhite_c52043_344
Excalibur_c49977_170
BobWhite_c13210_115
RAC875_rep_c109701_76
RAC875_c66845_466
BS00034334_51
Kukri_rep_c102953_304
wsnp_Ku_c40218_48484410
wsnp_Ex_rep_c106152_90334299
BS00022148_51
IAAV4781
BS00073009_51
TA002997-0276
gwm0720
Tdurum_contig62502_90
wmc0446
BobWhite_rep_c51301_1261
wsnp_Ex_c2331_4369782
wsnp_Ex_c3478_6369892
RAC875_rep_c117959_132
wsnp_Ex_rep_c69577_68526990
gwm1159
gwm1063
Ex_c9685_1264
IAAV242
wsnp_Ex_rep_c66685_65003625
wmc0660
gwm0638
gwm1042
wsnp_Ex_c13949_21828493
CAP12_c1787_110
Kukri_rep_c111139_338
RAC875_c9500_1770
wsnp_RFL_Contig2699_2402527
Tdurum_contig47509_93
wsnp_Ex_c8884_14841846
BS00022703_51
BS00022989_51
BS00038663_51
BS00038664_51
Excalibur_c39309_171
GENE-1661_228
GENE-1724_72
GENE-1929_91
IACX5909
RFL_Contig5871_1650
Tdurum_contig56373_348
Tdurum_contig56731_335
Tdurum_contig9912_228
Tdurum_contig9912_451
BS00022845_51
BS00067748_51
BS00100626_51
Tdurum_contig13548_158
Tdurum_contig9912_216
Excalibur_rep_c109598_66
Kukri_rep_c69970_717
BS00060391_51
TA003179-0872
IACX5899
wsnp_Ku_c55137_59154077
Kukri_c63259_151
RAC875_c52805_182
IAAV8218
wsnp_Ex_c27317_36522052
BS00081610_51
BS00060029_51
BS00088755_51
BS00088756_51
Excalibur_c49743_97
Tdurum_contig54800_615
cfa2193
BS00110564_51
BS00003935_51
BS00022245_51
BS00022459_51
wsnp_Ku_c10468_17301042
BS00024548_51
Excalibur_c24354_465
gwm1217
gwm4187b
cfa2183
BS00076772_51
wsnp_Ex_c1903_3592518
Kukri_rep_c106620_208
tplb0036i05_182
RAC875_c6006_105
BS00041742_51
Ra_c4373_453
wsnp_BE604885A_Ta_2_1
BS00093889_51
GENE-1776_104
wsnp_BE426418A_Ta_2_2
Kukri_c12212_182
Tdurum_contig38751_317
Tdurum_contig31235_99
wsnp_Ex_c11229_18163892
RAC875_c46236_214
Tdurum_contig93147_113

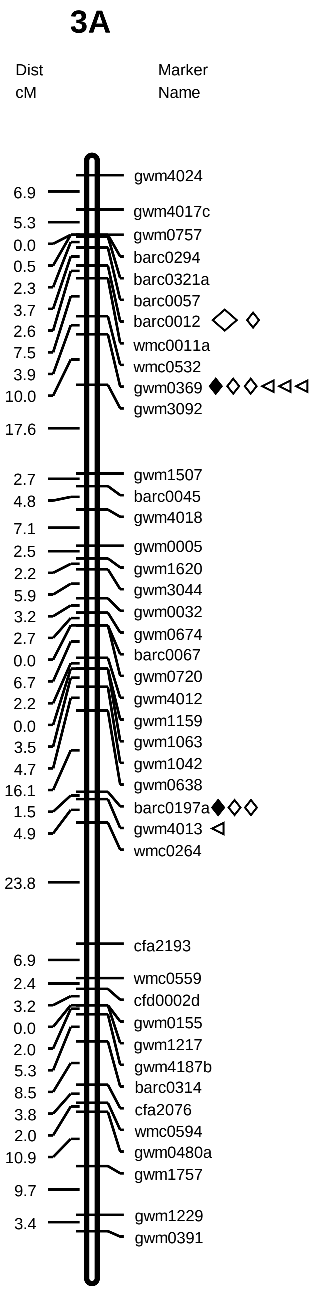
3A
Dist
cM
Marker
Name
6.9
5.3
0.0
0.5
2.3
3.7
2.6
7.5
3.9
10.0
17.6
2.7
4.8
7.1
2.5
2.2
5.9
3.2
2.7
0.0
6.7
2.2
0.0
3.5
4.7
16.1
1.5
4.9
23.8
6.9
2.4
3.2
0.0
2.0
5.3
8.5
3.8
2.0
10.9
9.7
3.4
gwm4024
gwm4017c
gwm0757
barc0294
barc0321a
barc0057
barc0012
wmc0011a
wmc0532
gwm0369
gwm3092
gwm1507
barc0045
gwm4018
gwm0005
gwm1620
gwm3044
gwm0032
gwm0674
barc0067
gwm0720
gwm4012
gwm1159
gwm1063
gwm1042
gwm0638
barc0197a
gwm4013
wmc0264
cfa2193
wmc0559
cfd0002d
gwm0155
gwm1217
gwm4187b
barc0314
cfa2076
wmc0594
gwm0480a
gwm1757
gwm1229
gwm0391

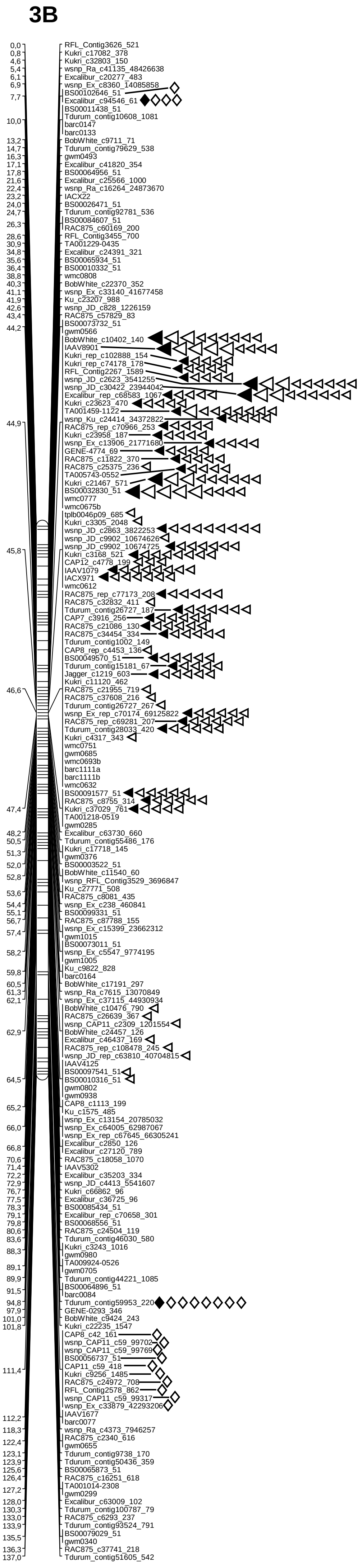
3B
0,0
0,8
4,6
5,4
6,1
6,9
7,7
10,0
13,2
14,7
16,3
17,1
17,8
21,6
22,4
23,2
24,0
24,7
26,3
28,6
30,9
34,8
35,6
36,4
38,8
40,3
41,1
41,9
42,6
43,4
44,2
44,9
45,8
46,6
47,4
48,2
50,5
51,3
52,0
52,8
53,6
54,4
55,1
56,7
57,4
58,2
59,8
60,5
61,3
62,1
62,9
64,5
65,2
66,0
66,8
70,6
71,4
72,2
72,9
76,7
77,5
78,3
79,1
79,8
80,6
83,6
88,3
89,1
89,9
91,5
94,8
97,9
101,0
101,8
111,4
112,2
118,3
122,4
123,1
123,9
125,6
126,4
127,2
128,0
130,3
133,0
133,9
135,5
136,3
137,0
RFL_Contig3626_521
Kukri_c17082_378
Kukri_c32803_150
wsnp_Ra_c41135_48426638
Excalibur_c20277_483
wsnp_Ex_c8360_14085858
BS00102646_51
Excalibur_c94546_61
BS00011438_51
Tdurum_contig10608_1081
barc0147
barc0133
BobWhite_c9711_71
Tdurum_contig79629_538
gwm0493
Excalibur_c41820_354
BS00064956_51
Excalibur_c25566_1000
wsnp_Ra_c16264_24873670
IACX22
BS00026471_51
Tdurum_contig92781_536
BS00084607_51
RAC875_c60169_200
RFL_Contig3455_700
TA001229-0435
Excalibur_c24391_321
BS00065934_51
BS00010332_51
wmc0808
BobWhite_c22370_352
wsnp_Ex_c33140_41677458
Ku_c23207_988
wsnp_JD_c828_1226159
RAC875_c57829_83
BS00073732_51
gwm0566
BobWhite_c10402_140
IAAV8901
Kukri_rep_c102888_154
Kukri_rep_c74178_178
RFL_Contig2267_1589
wsnp_JD_c2623_3541255
wsnp_JD_c30422_23944042
Excalibur_rep_c68583_1067
Kukri_c23623_470
TA001459-1122
wsnp_Ku_c24414_34372822
RAC875_rep_c70966_253
Kukri_c23958_187
wsnp_Ex_c13906_21771680
GENE-4774_69
RAC875_c11822_370
RAC875_c25375_236
TA005743-0552
Kukri_c21467_571
BS00032830_51
wmc0777
wmc0675b
tplb0046p09_685
Kukri_c3305_2048
wsnp_JD_c2863_3822253
wsnp_JD_c9902_10674626
wsnp_JD_c9902_10674725
Kukri_c3168_521
CAP12_c4778_199
IAAV1079
IACX971
wmc0612
RAC875_rep_c77173_208
RAC875_c32832_411
Tdurum_contig26727_187
CAP7_c3916_256
RAC875_c21086_130
RAC875_c34454_334
Tdurum_contig1002_149
CAP8_rep_c4453_136
BS00049570_51
Tdurum_contig15181_67
Jagger_c1219_603
Kukri_c11120_462
RAC875_c21955_719
RAC875_c37608_216
Tdurum_contig26727_267
wsnp_Ex_rep_c70174_69125822
RAC875_rep_c69281_207
Tdurum_contig28033_420
Kukri_c4317_343
wmc0751
gwm0685
wmc0693b
barc1111a
barc1111b
wmc0632
BS00091577_51
RAC875_c8755_314
Kukri_c37029_761
TA001218-0519
gwm0285
Excalibur_c63730_660
Tdurum_contig55486_176
Kukri_c17718_145
gwm0376
BS00003522_51
BobWhite_c11540_60
wsnp_RFL_Contig3529_3696847
Ku_c27771_508
RAC875_c8081_435
wsnp_Ex_c238_460841
BS00099331_51
RAC875_c87788_155
wsnp_Ex_c15399_23662312
gwm1015
BS00073011_51
wsnp_Ex_c5547_9774195
gwm1005
Ku_c9822_828
barc0164
BobWhite_c17191_297
wsnp_Ra_c7615_13070849
wsnp_Ex_c37115_44930934
BobWhite_c10476_790
RAC875_c26639_367
wsnp_CAP11_c2309_1201554
BobWhite_c24457_126
Excalibur_c46437_169
RAC875_rep_c108478_245
wsnp_JD_rep_c63810_40704815
IAAV4125
BS00097541_51
BS00010316_51
gwm0802
gwm0938
CAP8_c1113_199
Ku_c1575_485
wsnp_Ex_c13154_20785032
wsnp_Ex_c64005_62987067
wsnp_Ex_rep_c67645_66305241
Excalibur_c2850_126
Excalibur_c27120_789
RAC875_c18058_1070
IAAV5302
Excalibur_c35203_334
wsnp_JD_c4413_5541607
Kukri_c66862_96
Excalibur_c36725_96
BS00085434_51
Excalibur_rep_c70658_301
BS00068556_51
RAC875_c24504_119
Tdurum_contig46030_580
Kukri_c3243_1016
gwm0980
TA009924-0526
gwm0705
Tdurum_contig44221_1085
BS00064896_51
barc0084
Tdurum_contig59953_220
GENE-0293_346
BobWhite_c9424_243
Kukri_c22235_1547
CAP8_c42_161
wsnp_CAP11_c59_99702
wsnp_CAP11_c59_99769
BS00056737_51
CAP11_c59_418
Kukri_c9256_1485
RAC875_c24972_708
RFL_Contig2578_862
wsnp_CAP11_c59_99317
wsnp_Ex_c33879_42293206
IAAV1677
barc0077
wsnp_Ra_c4373_7946257
RAC875_c2340_616
gwm0655
Tdurum_contig9738_170
Tdurum_contig50436_359
BS00065873_51
RAC875_c16251_618
TA001014-2308
gwm0299
Excalibur_c63009_102
Tdurum_contig100787_79
RAC875_c6293_237
Tdurum_contig93524_791
BS00079029_51
gwm0340
RAC875_c37741_218
Tdurum_contig51605_542

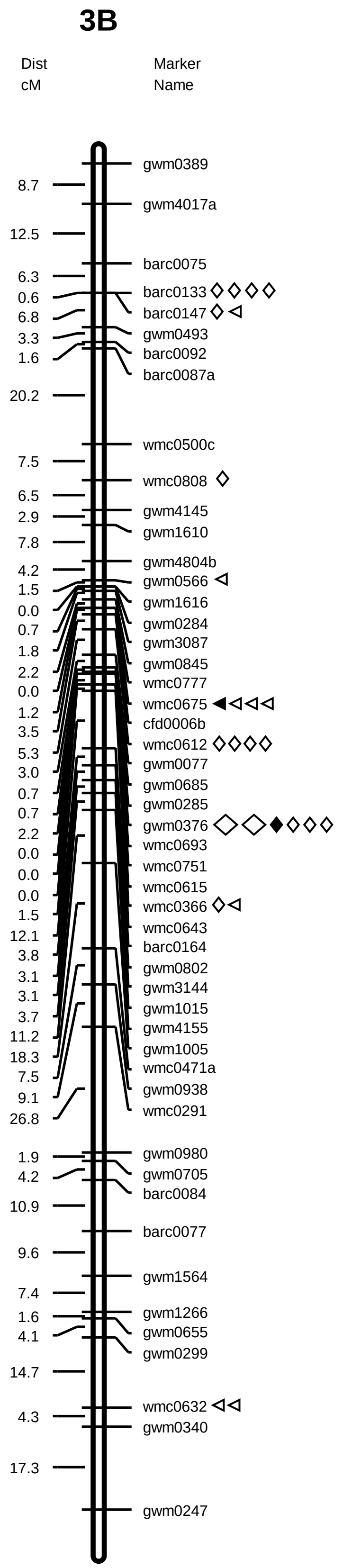
3B
Dist
cM
Marker
Name
gwm0389
8.7
gwm4017a
12.5
barc0075
6.3
barc0133
0.6
barc0147
6.8
gwm0493
3.3
barc0092
1.6
barc0087a
20.2
wmc0500c
7.5
wmc0808
6.5
gwm4145
2.9
gwm1610
7.8
gwm4804b
4.2
gwm0566
1.5
gwm1616
0.0
gwm0284
0.7
gwm3087
1.8
gwm0845
2.2
wmc0777
0.0
wmc0675
1.2
cfd0006b
3.5
wmc0612
5.3
gwm0077
3.0
gwm0685
0.7
gwm0285
0.7
gwm0376
2.2
wmc0693
0.0
wmc0751
0.0
wmc0615
0.0
wmc0366
1.5
wmc0643
12.1
barc0164
3.8
gwm0802
3.1
gwm3144
3.1
gwm1015
3.7
gwm4155
11.2
gwm1005
18.3
wmc0471a
7.5
gwm0938
9.1
wmc0291
26.8
gwm0980
1.9
gwm0705
4.2
barc0084
10.9
barc0077
9.6
gwm1564
7.4
gwm1266
1.6
gwm0655
4.1
gwm0299
14.7
wmc0632
4.3
gwm0340
17.3
gwm0247

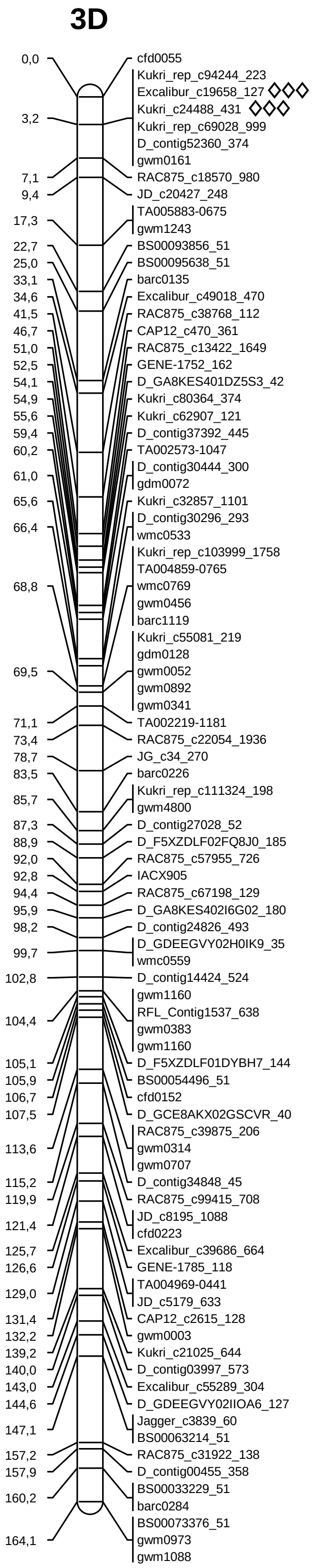

3D
0,0
3,2
7,1
9,4
17,3
22,7
25,0
33,1
34,6
41,5
46,7
51,0
52,5
54,1
54,9
55,6
59,4
60,2
61,0
65,6
66,4
68,8
69,5
71,1
73,4
78,7
83,5
85,7
87,3
88,9
92,0
92,8
94,4
95,9
98,2
99,7
102,8
104,4
105,1
105,9
106,7
107,5
113,6
115,2
119,9
121,4
125,7
126,6
129,0
131,4
132,2
139,2
140,0
143,0
144,6
147,1
157,2
157,9
160,2
164,1
cfd0055
Kukri_rep_c94244_223
Excalibur_c19658_127
Kukri_c24488_431
Kukri_rep_c69028_999
D_contig52360_374
gwm0161
RAC875_c18570_980
JD_c20427_248
TA005883-0675
gwm1243
BS00093856_51
BS00095638_51
barc0135
Excalibur_c49018_470
RAC875_c38768_112
CAP12_c470_361
RAC875_c13422_1649
GENE-1752_162
D_GA8KES401DZ5S3_42
Kukri_c80364_374
Kukri_c62907_121
D_contig37392_445
TA002573-1047
D_contig30444_300
gdm0072
Kukri_c32857_1101
D_contig30296_293
wmc0533
Kukri_rep_c103999_1758
TA004859-0765
wmc0769
gwm0456
barc1119
Kukri_c55081_219
gdm0128
gwm0052
gwm0892
gwm0341
TA002219-1181
RAC875_c22054_1936
JG_c34_270
barc0226
Kukri_rep_c111324_198
gwm4800
D_contig27028_52
D_F5XZDLF02FQ8J0_185
RAC875_c57955_726
IACX905
RAC875_c67198_129
D_GA8KES402I6G02_180
D_contig24826_493
D_GDEEGVY02H0IK9_35
wmc0559
D_contig14424_524
gwm1160
RFL_Contig1537_638
gwm0383
gwm1160
D_F5XZDLF01DYBH7_144
BS00054496_51
cfd0152
D_GCE8AKX02GSCVR_40
RAC875_c39875_206
gwm0314
gwm0707
D_contig34848_45
RAC875_c99415_708
JD_c8195_1088
cfd0223
Excalibur_c39686_664
GENE-1785_118
TA004969-0441
JD_c5179_633
CAP12_c2615_128
gwm0003
Kukri_c21025_644
D_contig03997_573
Excalibur_c55289_304
D_GDEEGVY02IIOA6_127
Jagger_c3839_60
BS00063214_51
RAC875_c31922_138
D_contig00455_358
BS00033229_51
barc0284
BS00073376_51
gwm0973
gwm1088

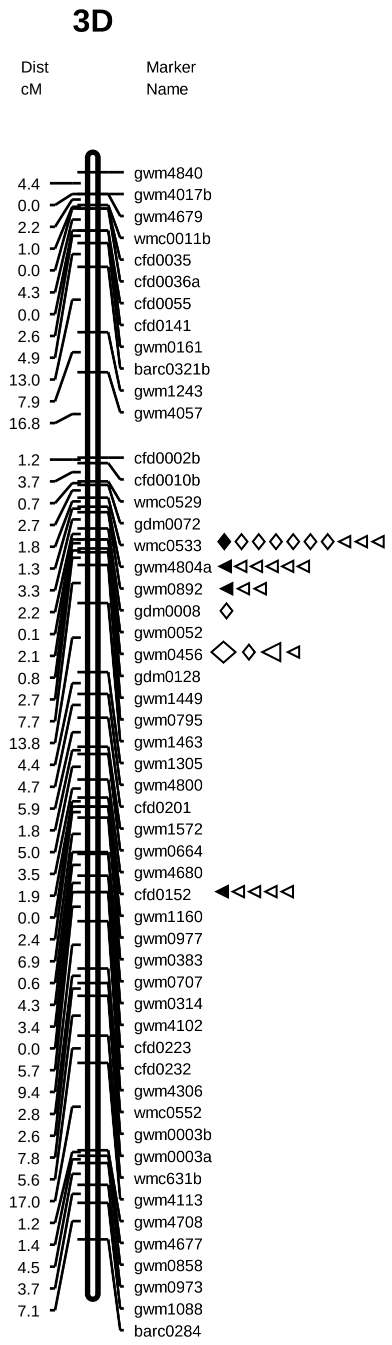

3D
Dist
cM
Marker
Name
4.4
0.0
2.2
1.0
0.0
4.3
0.0
2.6
4.9
13.0
7.9
16.8
1.2
3.7
0.7
2.7
1.8
1.3
3.3
2.2
0.1
2.1
0.8
2.7
7.7
13.8
4.4
4.7
5.9
1.8
5.0
3.5
1.9
0.0
2.4
6.9
0.6
4.3
3.4
0.0
5.7
9.4
2.8
2.6
7.8
5.6
17.0
1.2
1.4
4.5
3.7
7.1
gwm4840
gwm4017b
gwm4679
wmc0011b
cfd0035
cfd0036a
cfd0055
cfd0141
gwm0161
barc0321b
gwm1243
gwm4057
cfd0002b
cfd0010b
wmc0529
gdm0072
wmc0533
gwm4804a
gwm0892
gdm0008
gwm0052
gwm0456
gdm0128
gwm1449
gwm0795
gwm1463
gwm1305
gwm4800
cfd0201
gwm1572
gwm0664
gwm4680
cfd0152
gwm1160
gwm0977
gwm0383
gwm0707
gwm0314
gwm4102
cfd0223
cfd0232
gwm4306
wmc0552
gwm0003b
gwm0003a
wmc631b
gwm4113
gwm4708
gwm4677
gwm0858
gwm0973
gwm1088
barc0284

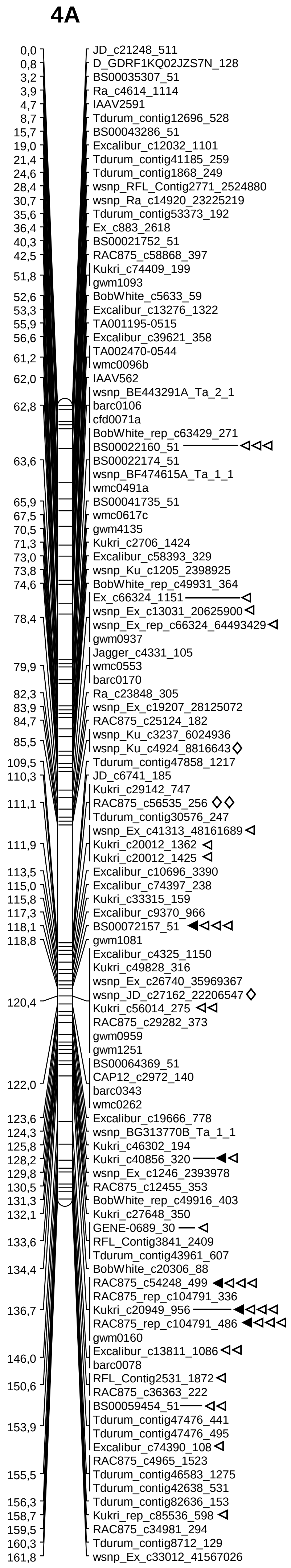
4A
0,0
0,8
3,2
3,9
4,7
8,7
15,7
19,0
21,4
24,6
28,4
30,7
35,6
36,4
40,3
42,5
51,8
52,6
53,3
55,9
56,6
61,2
62,0
62,8
63,6
65,9
67,5
70,5
71,3
73,0
73,8
74,6
78,4
79,9
82,3
83,9
84,7
85,5
109,5
110,3
111,1
111,9
113,5
115,0
115,8
117,3
118,1
118,8
120,4
122,0
123,6
124,3
125,8
128,2
129,8
130,5
131,3
132,1
133,6
134,4
136,7
146,0
150,6
153,9
155,5
156,3
158,7
159,5
160,3
161,8
JD_c21248_511
D_GDRF1KQ02JZS7N_128
BS00035307_51
Ra_c4614_1114
IAAV2591
Tdurum_contig12696_528
BS00043286_51
Excalibur_c12032_1101
Tdurum_contig41185_259
Tdurum_contig1868_249
wsnp_RFL_Contig2771_2524880
wsnp_Ra_c14920_23225219
Tdurum_contig53373_192
Ex_c883_2618
BS00021752_51
RAC875_c58868_397
Kukri_c74409_199
gwm1093
BobWhite_c5633_59
Excalibur_c13276_1322
TA001195-0515
Excalibur_c39621_358
TA002470-0544
wmc0096b
IAAV562
wsnp_BE443291A_Ta_2_1
barc0106
cfd0071a
BobWhite_rep_c63429_271
BS00022160_51
BS00022174_51
wsnp_BF474615A_Ta_1_1
wmc0491a
BS00041735_51
wmc0617c
gwm4135
Kukri_c2706_1424
Excalibur_c58393_329
wsnp_Ku_c1205_2398925
BobWhite_rep_c49931_364
Ex_c66324_1151
wsnp_Ex_c13031_20625900
wsnp_Ex_rep_c66324_64493429
gwm0937
Jagger_c4331_105
wmc0553
barc0170
Ra_c23848_305
wsnp_Ex_c19207_28125072
RAC875_c25124_182
wsnp_Ku_c3237_6024936
wsnp_Ku_c4924_8816643
Tdurum_contig47858_1217
JD_c6741_185
Kukri_c29142_747
RAC875_c56535_256
Tdurum_contig30576_247
wsnp_Ex_c41313_48161689
Kukri_c20012_1362
Kukri_c20012_1425
Excalibur_c10696_3390
Excalibur_c74397_238
Kukri_c33315_159
Excalibur_c9370_966
BS00072157_51
gwm1081
Excalibur_c4325_1150
Kukri_c49828_316
wsnp_Ex_c26740_35969367
wsnp_JD_c27162_22206547
Kukri_c56014_275
RAC875_c29282_373
gwm0959
gwm1251
BS00064369_51
CAP12_c2972_140
barc0343
wmc0262
Excalibur_c19666_778
wsnp_BG313770B_Ta_1_1
Kukri_c46302_194
Kukri_c40856_320
wsnp_Ex_c1246_2393978
RAC875_c12455_353
BobWhite_rep_c49916_403
Kukri_c27648_350
GENE-0689_30
RFL_Contig3841_2409
Tdurum_contig43961_607
BobWhite_c20306_88
RAC875_c54248_499
RAC875_rep_c104791_336
Kukri_c20949_956
RAC875_rep_c104791_486
gwm0160
Excalibur_c13811_1086
barc0078
RFL_Contig2531_1872
RAC875_c36363_222
BS00059454_51
Tdurum_contig47476_441
Tdurum_contig47476_495
Excalibur_c74390_108
RAC875_c4965_1523
Tdurum_contig46583_1275
Tdurum_contig42638_531
Tdurum_contig82636_153
Kukri_rep_c85536_598
RAC875_c34981_294
Tdurum_contig8712_129
wsnp_Ex_c33012_41567026

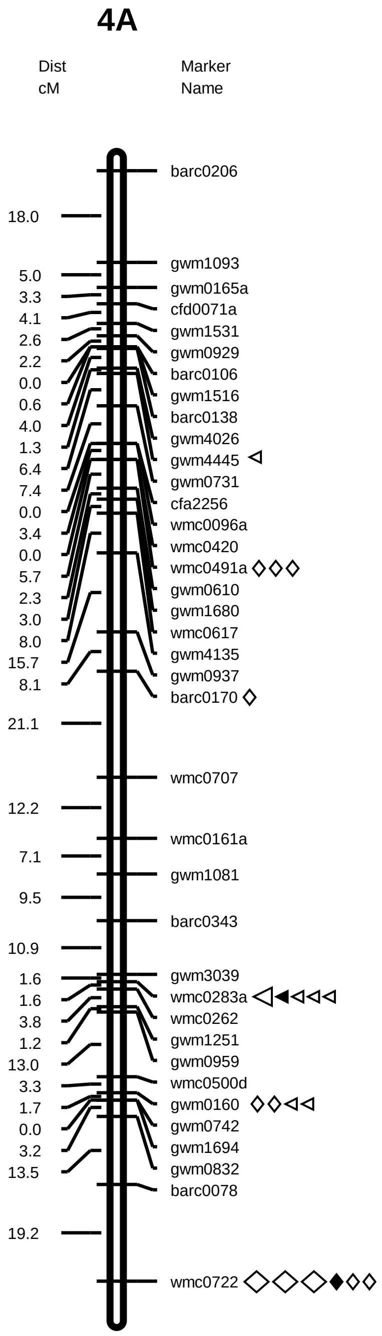
4A
Dist
cM
Marker
Name
18.0
5.0
3.3
4.1
2.6
2.2
0.0
0.6
4.0
1.3
6.4
7.4
0.0
3.4
0.0
5.7
2.3
3.0
8.0
15.7
8.1
21.1
12.2
7.1
9.5
10.9
1.6
1.6
3.8
1.2
13.0
3.3
1.7
0.0
3.2
13.5
19.2
barc0206
gwm1093
gwm0165a
cfd0071a
gwm1531
gwm0929
barc0106
gwm1516
barc0138
gwm4026
gwm4445
gwm0731
cfa2256
wmc0096a
wmc0420
wmc0491a
gwm0610
gwm1680
wmc0617
gwm4135
gwm0937
barc0170
wmc0707
wmc0161a
gwm1081
barc0343
gwm3039
wmc0283a
wmc0262
gwm1251
gwm0959
wmc0500d
gwm0160
gwm0742
gwm1694
gwm0832
barc0078
wmc0722

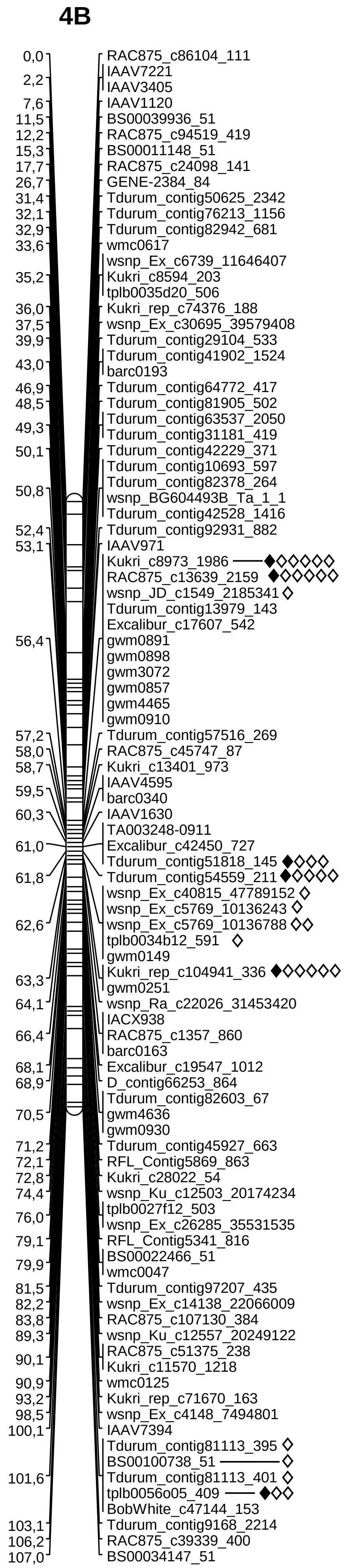
4B
0,0
2,2
7,6
11,5
12,2
15,3
17,7
26,7
31,4
32,1
32,9
33,6
35,2
36,0
37,5
39,9
43,0
46,9
48,5
49,3
50,1
50,8
52,4
53,1
56,4
57,2
58,0
58,7
59,5
60,3
61,0
61,8
62,6
63,3
64,1
66,4
68,1
68,9
70,5
71,2
72,1
72,8
74,4
76,0
79,1
79,9
81,5
82,2
83,8
89,3
90,1
90,9
93,2
98,5
100,1
101,6
103,1
106,2
107,0
RAC875_c86104_111
IAAV7221
IAAV3405
IAAV1120
BS00039936_51
RAC875_c94519_419
BS00011148_51
RAC875_c24098_141
GENE-2384_84
Tdurum_contig50625_2342
Tdurum_contig76213_1156
Tdurum_contig82942_681
wmc0617
wsnp_Ex_c6739_11646407
Kukri_c8594_203
tplb0035d20_506
Kukri_rep_c74376_188
wsnp_Ex_c30695_39579408
Tdurum_contig29104_533
Tdurum_contig41902_1524
barc0193
Tdurum_contig64772_417
Tdurum_contig81905_502
Tdurum_contig63537_2050
Tdurum_contig31181_419
Tdurum_contig42229_371
Tdurum_contig10693_597
Tdurum_contig82378_264
wsnp_BG604493B_Ta_1_1
Tdurum_contig42528_1416
Tdurum_contig92931_882
IAAV971
Kukri_c8973_1986
RAC875_c13639_2159
wsnp_JD_c1549_2185341
Tdurum_contig13979_143
Excalibur_c17607_542
gwm0891
gwm0898
gwm3072
gwm0857
gwm4465
gwm0910
Tdurum_contig57516_269
RAC875_c45747_87
Kukri_c13401_973
IAAV4595
barc0340
IAAV1630
TA003248-0911
Excalibur_c42450_727
Tdurum_contig51818_145
Tdurum_contig54559_211
wsnp_Ex_c40815_47789152
wsnp_Ex_c5769_10136243
wsnp_Ex_c5769_10136788
tplb0034b12_591
gwm0149
Kukri_rep_c104941_336
gwm0251
wsnp_Ra_c22026_31453420
IACX938
RAC875_c1357_860
barc0163
Excalibur_c19547_1012
D_contig66253_864
Tdurum_contig82603_67
gwm4636
gwm0930
Tdurum_contig45927_663
RFL_Contig5869_863
Kukri_c28022_54
wsnp_Ku_c12503_20174234
tplb0027f12_503
wsnp_Ex_c26285_35531535
RFL_Contig5341_816
BS00022466_51
wmc0047
Tdurum_contig97207_435
wsnp_Ex_c14138_22066009
RAC875_c107130_384
wsnp_Ku_c12557_20249122
RAC875_c51375_238
Kukri_c11570_1218
wmc0125
Kukri_rep_c71670_163
wsnp_Ex_c4148_7494801
IAAV7394
Tdurum_contig81113_395
BS00100738_51
Tdurum_contig81113_401
tplb0056o05_409
BobWhite_c47144_153
Tdurum_contig9168_2214
RAC875_c39339_400
BS00034147_51

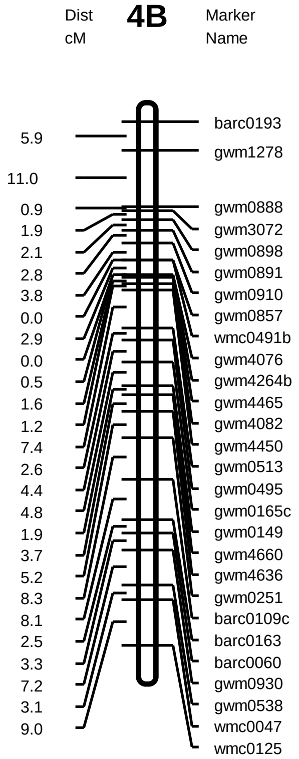
Dist
cM
4B
Marker
Name
5.9
11.0
0.9
1.9
2.1
2.8
3.8
0.0
2.9
0.0
0.5
1.6
1.2
7.4
2.6
4.4
4.8
1.9
3.7
5.2
8.3
8.1
2.5
3.3
7.2
3.1
9.0
barc0193
gwm1278
gwm0888
gwm3072
gwm0898
gwm0891
gwm0910
gwm0857
wmc0491b
gwm4076
gwm4264b
gwm4465
gwm4082
gwm4450
gwm0513
gwm0495
gwm0165c
gwm0149
gwm4660
gwm4636
gwm0251
barc0109c
barc0163
barc0060
gwm0930
gwm0538
wmc0047
wmc0125

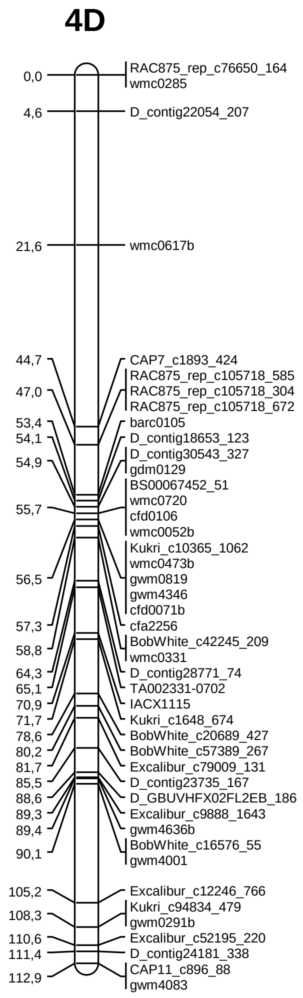
4D
0,0
4,6
21,6
44,7
47,0
53,4
54,1
54,9
55,7
56,5
57,3
58,8
64,3
65,1
70,9
71,7
78,6
80,2
81,7
85,5
88,6
89,3
89,4
90,1
105,2
108,3
110,6
111,4
112,9
RAC875_rep_c76650_164
wmc0285
D_contig22054_207
wmc0617b
CAP7_c1893_424
RAC875_rep_c105718_585
RAC875_rep_c105718_304
RAC875_rep_c105718_672
barc0105
D_contig18653_123
D_contig30543_327
gdm0129
BS00067452_51
wmc0720
cfd0106
wmc0052b
Kukri_c10365_1062
wmc0473b
gwm0819
gwm4346
cfd0071b
cfa2256
BobWhite_c42245_209
wmc0331
D_contig28771_74
TA002331-0702
IACX1115
Kukri_c1648_674
BobWhite_c20689_427
BobWhite_c57389_267
Excalibur_c79009_131
D_contig23735_167
D_GBUVHFX02FL2EB_186
Excalibur_c9888_1643
gwm4636b
BobWhite_c16576_55
gwm4001
Excalibur_c12246_766
Kukri_c94834_479
gwm0291b
Excalibur_c52195_220
D_contig24181_338
CAP11_c896_88
gwm4083

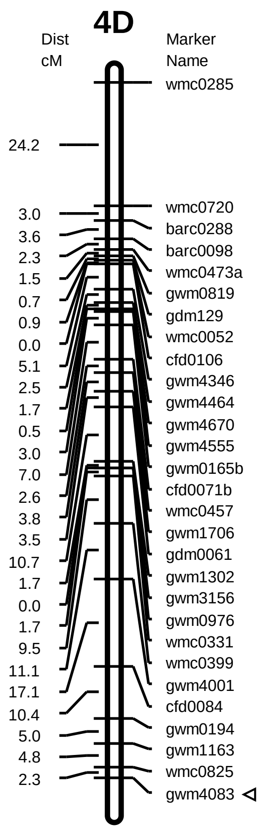
4D
Dist cM
Marker Name
24.2
3.0
3.6
2.3
1.5
0.7
0.9
0.0
5.1
2.5
1.7
0.5
3.0
7.0
2.6
3.8
3.5
10.7
1.7
0.0
1.7
9.5
11.1
17.1
10.4
5.0
4.8
2.3
wmc0285
wmc0720
barc0288
barc0098
wmc0473a
gwm0819
gdm129
wmc0052
cfd0106
gwm4346
gwm4464
gwm4670
gwm4555
gwm0165b
cfd0071b
wmc0457
gwm1706
gdm0061
gwm1302
gwm3156
gwm0976
wmc0331
wmc0399
gwm4001
cfd0084
gwm0194
gwm1163
wmc0825
gwm4083

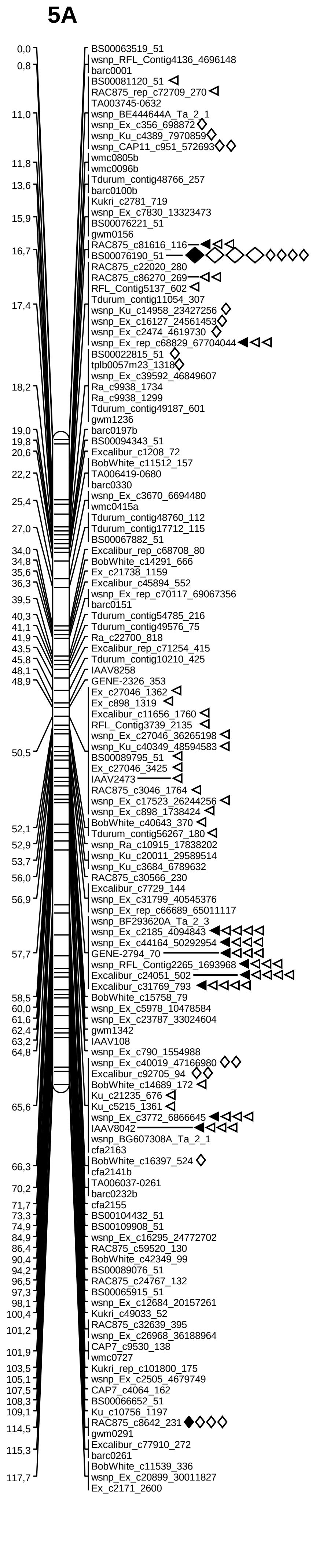
5A
0,0
0,8
11,0
11,8
13,6
15,9
16,7
17,4
18,2
19,0
19,8
20,6
22,2
25,4
27,0
34,0
34,8
35,6
36,3
39,5
40,3
41,1
41,9
43,5
45,8
48,1
48,9
50,5
52,1
52,9
53,7
56,0
56,9
57,7
58,5
60,0
61,6
62,4
63,2
64,8
65,6
66,3
70,2
71,7
73,3
74,9
84,9
86,4
90,4
94,2
96,5
97,3
98,1
100,4
101,2
101,9
103,5
105,1
107,5
108,3
109,1
114,5
115,3
117,7
BS00063519_51
wsnp_RFL_Contig4136_4696148
barc0001
BS00081120_51
RAC875_rep_c72709_270
TA003745-0632
wsnp_BE444644A_Ta_2_1
wsnp_Ex_c356_698872
wsnp_Ku_c4389_7970859
wsnp_CAP11_c951_572693
wmc0805b
wmc0096b
Tdurum_contig48766_257
barc0100b
Kukri_c2781_719
wsnp_Ex_c7830_13323473
BS00076221_51
gwm0156
RAC875_c81616_116
BS00076190_51
RAC875_c22020_280
RAC875_c86270_269
RFL_Contig5137_602
Tdurum_contig11054_307
wsnp_Ku_c14958_23427256
wsnp_Ex_c16127_24561453
wsnp_Ex_c2474_4619730
wsnp_Ex_rep_c68829_67704044
BS00022815_51
tplb0057m23_1318
wsnp_Ex_c39592_46849607
Ra_c9938_1734
Ra_c9938_1299
Tdurum_contig49187_601
gwm1236
barc0197b
BS00094343_51
Excalibur_c1208_72
BobWhite_c11512_157
TA006419-0680
barc0330
wsnp_Ex_c3670_6694480
wmc0415a
Tdurum_contig48760_112
Tdurum_contig17712_115
BS00067882_51
Excalibur_rep_c68708_80
BobWhite_c14291_666
Ex_c21738_1159
Excalibur_c45894_552
wsnp_Ex_rep_c70117_69067356
barc0151
Tdurum_contig54785_216
Tdurum_contig49576_75
Ra_c22700_818
Excalibur_rep_c71254_415
Tdurum_contig10210_425
IAAV8258
GENE-2326_353
Ex_c27046_1362
Ex_c898_1319
Excalibur_c11656_1760
RFL_Contig3739_2135
wsnp_Ex_c27046_36265198
wsnp_Ku_c40349_48594583
BS00089795_51
Ex_c27046_3425
IAAV2473
RAC875_c3046_1764
wsnp_Ex_c17523_26244256
wsnp_Ex_c898_1738424
BobWhite_c40643_370
Tdurum_contig56267_180
wsnp_Ra_c10915_17838202
wsnp_Ku_c20011_29589514
wsnp_Ku_c3684_6789632
RAC875_c30566_230
Excalibur_c7729_144
wsnp_Ex_c31799_40545376
wsnp_Ex_rep_c66689_65011117
wsnp_BF293620A_Ta_2_3
wsnp_Ex_c2185_4094843
wsnp_Ex_c44164_50292954
GENE-2794_70
wsnp_RFL_Contig2265_1693968
Excalibur_c24051_502
Excalibur_c31769_793
BobWhite_c15758_79
wsnp_Ex_c5978_10478584
wsnp_Ex_c23787_33024604
gwm1342
IAAV108
wsnp_Ex_c790_1554988
wsnp_Ex_c40019_47166980
Excalibur_c92705_94
BobWhite_c14689_172
Ku_c21235_676
Ku_c5215_1361
wsnp_Ex_c3772_6866645
IAAV8042
wsnp_BG607308A_Ta_2_1
cfa2163
BobWhite_c16397_524
cfa2141b
TA006037-0261
barc0232b
cfa2155
BS00104432_51
BS00109908_51
wsnp_Ex_c16295_24772702
RAC875_c59520_130
BobWhite_c42349_99
BS00089076_51
RAC875_c24767_132
BS00065915_51
wsnp_Ex_c12684_20157261
Kukri_c49033_52
RAC875_c32639_395
wsnp_Ex_c26968_36188964
CAP7_c9530_138
wmc0727
Kukri_rep_c101800_175
wsnp_Ex_c2505_4679749
CAP7_c4064_162
BS00066652_51
Ku_c10756_1197
RAC875_c8642_231
gwm0291
Excalibur_c77910_272
barc0261
BobWhite_c11539_336
wsnp_Ex_c20899_30011827
Ex_c2171_2600

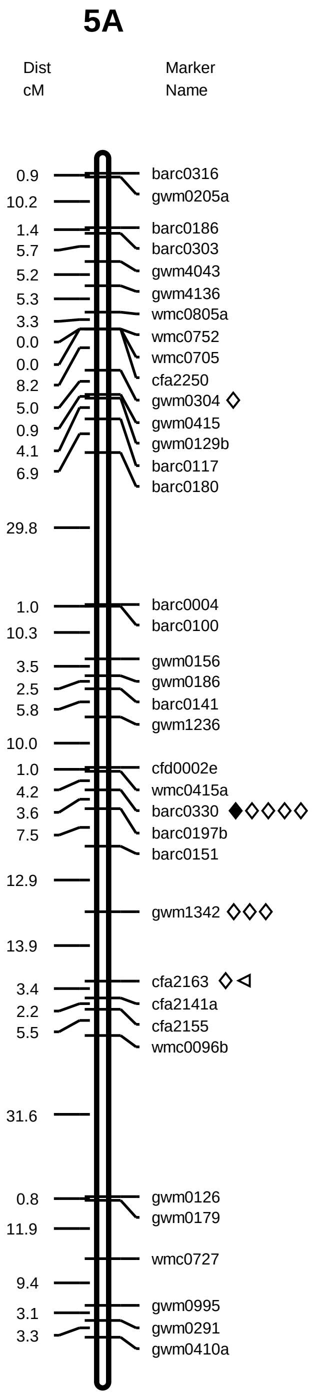
5A
Dist
cM
Marker
Name
0.9
10.2
1.4
5.7
5.2
5.3
3.3
0.0
0.0
8.2
5.0
0.9
4.1
6.9
29.8
1.0
10.3
3.5
2.5
5.8
10.0
1.0
4.2
3.6
7.5
12.9
13.9
3.4
2.2
5.5
31.6
0.8
11.9
9.4
3.1
3.3
barc0316
gwm0205a
barc0186
barc0303
gwm4043
gwm4136
wmc0805a
wmc0752
wmc0705
cfa2250
gwm0304
gwm0415
gwm0129b
barc0117
barc0180
barc0004
barc0100
gwm0156
gwm0186
barc0141
gwm1236
cfd0002e
wmc0415a
barc0330
barc0197b
barc0151
gwm1342
cfa2163
cfa2141a
cfa2155
wmc0096b
gwm0126
gwm0179
wmc0727
gwm0995
gwm0291
gwm0410a

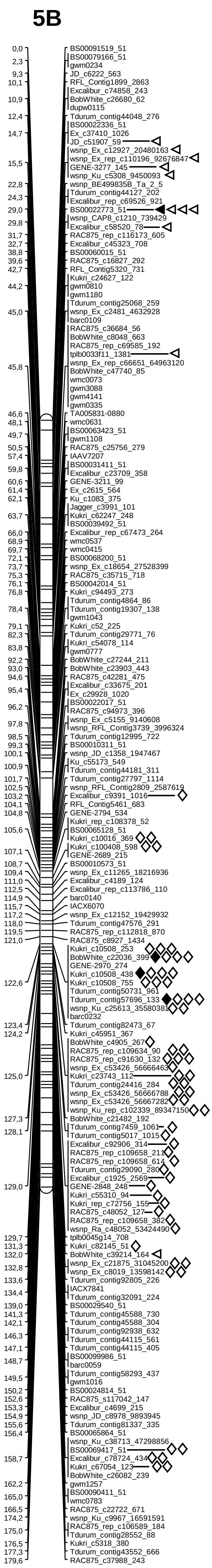

5B
0,0
2,3
9,3
10,1
10,9
12,4
14,7
15,5
22,8
24,3
29,0
29,8
31,7
32,7
38,8
39,6
42,7
44,2
45,0
45,8
46,6
48,1
49,7
50,5
57,4
59,8
60,6
61,4
62,1
63,7
66,0
68,9
69,7
72,1
73,7
75,3
76,1
76,8
78,4
79,1
82,3
83,8
92,2
93,0
94,6
95,4
96,2
97,8
98,5
99,3
100,1
100,9
101,7
102,5
103,2
104,1
104,8
105,6
107,1
108,7
109,4
111,0
112,5
114,9
115,7
117,2
118,0
119,5
121,0
122,6
123,4
124,2
125,0
127,3
128,1
129,0
129,7
131,3
132,0
132,8
133,6
134,4
139,0
141,3
142,1
146,3
147,1
148,7
149,5
150,2
152,6
153,3
154,9
155,6
156,4
158,7
162,2
165,0
166,5
174,2
175,0
176,5
177,3
179,6
BS00091519_51
BS00079166_51
gwm0234
JD_c6222_563
RFL_Contig1899_2863
Excalibur_c74858_243
BobWhite_c26680_62
dupw0115
Tdurum_contig44048_276
BS00022336_51
Ex_c37410_1026
JD_c51907_59
wsnp_Ex_c12927_20480163
wsnp_Ex_rep_c110196_92676847
GENE-3277_145
wsnp_Ku_c5308_9450093
wsnp_BE499835B_Ta_2_5
Tdurum_contig44127_202
Excalibur_rep_c69526_921
BS00022773_51
wsnp_CAP8_c1210_739429
Excalibur_c58520_78
RAC875_rep_c116173_605
Excalibur_c45323_708
BS00060015_51
RAC875_c16827_292
RFL_Contig5320_731
Kukri_c24627_122
gwm0810
gwm1180
Tdurum_contig25068_259
wsnp_Ex_c2481_4632928
barc0109
RAC875_c36684_56
BobWhite_c8048_663
RAC875_rep_c69585_192
tplb0033f11_1381
wsnp_Ex_rep_c66651_64963120
BobWhite_c47740_85
wmc0073
gwm3088
gwm4141
gwm0335
TA005831-0880
wmc0631
BS00063423_51
gwm1108
RAC875_c25756_279
IAAV7207
BS00031411_51
Excalibur_c23709_358
GENE-3211_99
Ex_c2615_564
Ku_c1083_375
Jagger_c3991_101
Kukri_c62247_248
BS00039492_51
Excalibur_rep_c67473_264
wmc0537
wmc0415
BS00068200_51
wsnp_Ex_c18654_27528399
RAC875_c35715_718
BS00042014_51
Kukri_c94493_273
Tdurum_contig4864_86
Tdurum_contig19307_138
gwm1043
Kukri_c52_225
Tdurum_contig29771_76
Kukri_c54078_114
gwm0777
BobWhite_c27244_211
BobWhite_c23903_443
RAC875_c42281_475
Excalibur_c33675_201
Ex_c29928_1020
BS00022017_51
RAC875_c94973_396
wsnp_Ex_c5155_9140608
wsnp_RFL_Contig3739_3996324
Tdurum_contig12995_722
BS00010311_51
wsnp_JD_c1358_1947467
Ku_c55173_549
Tdurum_contig44181_311
Tdurum_contig27797_1114
wsnp_RFL_Contig2809_2587619
Excalibur_c9391_1016
RFL_Contig5461_683
GENE-2794_534
Kukri_rep_c108378_52
BS00065128_51
Kukri_c10016_369
Kukri_c100408_598
GENE-2689_215
BS00010573_51
wsnp_Ex_c11265_18216936
Excalibur_c4189_124
Excalibur_rep_c113786_110
barc0140
IACX6070
wsnp_Ex_c12152_19429932
Tdurum_contig47576_291
RAC875_rep_c112818_870
RAC875_c8927_1434
Kukri_c10508_253
BobWhite_c22036_399
GENE-2970_274
Kukri_c10508_438
Kukri_c10508_755
Tdurum_contig50731_961
Tdurum_contig57696_133
wsnp_Ku_c25613_35580381
barc0232
Tdurum_contig82473_67
Kukri_c45951_367
BobWhite_c4905_267
RAC875_rep_c109634_90
RAC875_rep_c91630_132
wsnp_Ex_c53426_56666463
Kukri_c23743_112
Tdurum_contig24416_284
wsnp_Ex_c53426_56666788
wsnp_Ex_c53426_56667282
wsnp_Ku_rep_c102339_89347150
BobWhite_c21482_192
Tdurum_contig7459_1061
Tdurum_contig5017_1015
Excalibur_c92906_314
RAC875_rep_c109658_211
RAC875_rep_c109658_614
Tdurum_contig29090_280
Excalibur_c1925_2569
GENE-2848_248
Kukri_c55310_94
Kukri_rep_c72756_155
RAC875_c48052_127
RAC875_rep_c109658_382
wsnp_Ra_c48052_53424490
tplb0045g14_708
Kukri_c82145_51
BobWhite_c39214_164
wsnp_Ex_c21875_31045200
wsnp_Ex_c8019_13598142
Tdurum_contig92805_226
IACX7841
Tdurum_contig32091_224
BS00029540_51
Tdurum_contig45588_730
Tdurum_contig45588_304
Tdurum_contig92938_632
Tdurum_contig44115_561
Tdurum_contig44115_405
BS00099986_51
barc0059
Tdurum_contig58293_437
gwm1016
BS00024814_51
RAC875_s117042_147
Excalibur_c4699_215
wsnp_JD_c8978_9893945
Tdurum_contig81337_335
BS00065864_51
wsnp_Ku_c38713_47298856
BS00069417_51
Excalibur_c78724_434
Kukri_c67054_123
BobWhite_c26082_239
gwm1257
BS00090411_51
wmc0783
RAC875_c22722_671
wsnp_Ku_c9967_16591591
RAC875_rep_c106589_184
Tdurum_contig28552_88
Kukri_c5318_380
Tdurum_contig43552_666
RAC875_c37988_243

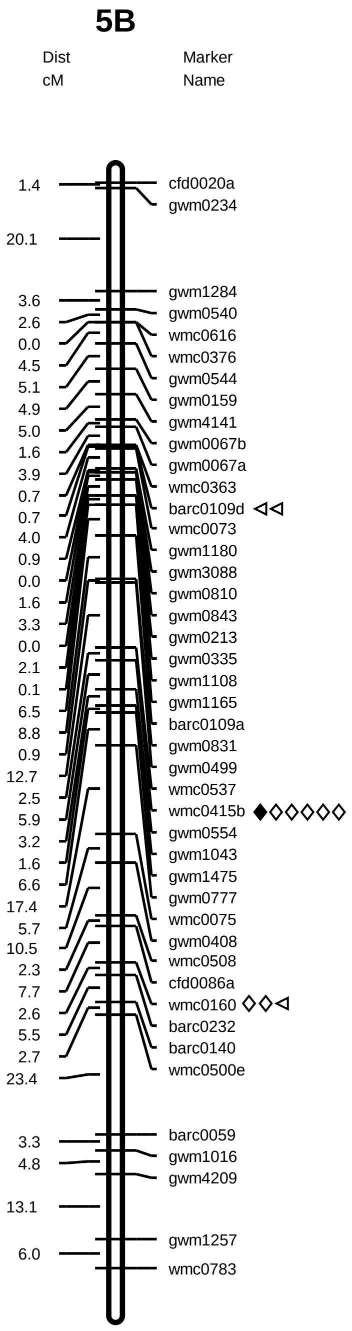

5B
Dist
cM
Marker
Name
1.4
20.1
3.6
2.6
0.0
4.5
5.1
4.9
5.0
1.6
3.9
0.7
0.7
4.0
0.9
0.0
1.6
3.3
0.0
2.1
0.1
6.5
8.8
0.9
12.7
2.5
5.9
3.2
1.6
6.6
17.4
5.7
10.5
2.3
7.7
2.6
5.5
2.7
23.4
3.3
4.8
13.1
6.0
cfd0020a
gwm0234
gwm1284
gwm0540
wmc0616
wmc0376
gwm0544
gwm0159
gwm4141
gwm0067b
gwm0067a
wmc0363
barc0109d
wmc0073
gwm1180
gwm3088
gwm0810
gwm0843
gwm0213
gwm0335
gwm1108
gwm1165
barc0109a
gwm0831
gwm0499
wmc0537
wmc0415b
gwm0554
gwm1043
gwm1475
gwm0777
wmc0075
gwm0408
wmc0508
cfd0086a
wmc0160
barc0232
barc0140
wmc0500e
barc0059
gwm1016
gwm4209
gwm1257
wmc0783

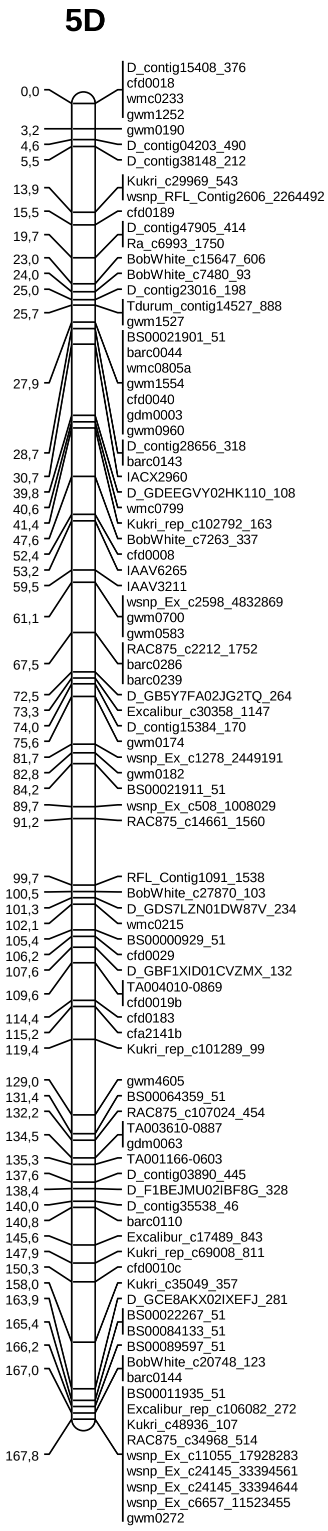
5D
0,0
D_contig15408_376
cfd0018
wmc0233
gwm1252
3,2
gwm0190
4,6
D_contig04203_490
5,5
D_contig38148_212
13,9
Kukri_c29969_543
wsnp_RFL_Contig2606_2264492
15,5
cfd0189
19,7
D_contig47905_414
Ra_c6993_1750
23,0
BobWhite_c15647_606
24,0
BobWhite_c7480_93
25,0
D_contig23016_198
25,7
Tdurum_contig14527_888
gwm1527
27,9
BS00021901_51
barc0044
wmc0805a
gwm1554
cfd0040
gdm0003
gwm0960
28,7
D_contig28656_318
barc0143
30,7
IACX2960
39,8
D_GDEEGVY02HK110_108
40,6
wmc0799
41,4
Kukri_rep_c102792_163
47,6
BobWhite_c7263_337
52,4
cfd0008
53,2
IAAV6265
59,5
IAAV3211
61,1
wsnp_Ex_c2598_4832869
gwm0700
gwm0583
67,5
RAC875_c2212_1752
barc0286
barc0239
72,5
D_GB5Y7FA02JG2TQ_264
73,3
Excalibur_c30358_1147
74,0
D_contig15384_170
75,6
gwm0174
81,7
wsnp_Ex_c1278_2449191
82,8
gwm0182
84,2
BS00021911_51
89,7
wsnp_Ex_c508_1008029
91,2
RAC875_c14661_1560
99,7
RFL_Contig1091_1538
100,5
BobWhite_c27870_103
101,3
D_GDS7LZN01DW87V_234
102,1
wmc0215
105,4
BS00000929_51
106,2
cfd0029
107,6
D_GBF1XID01CVZMX_132
109,6
TA004010-0869
cfd0019b
114,4
cfd0183
115,2
cfa2141b
119,4
Kukri_rep_c101289_99
129,0
gwm4605
131,4
BS00064359_51
132,2
RAC875_c107024_454
134,5
TA003610-0887
gdm0063
135,3
TA001166-0603
137,6
D_contig03890_445
138,4
D_F1BEJMU02IBF8G_328
140,0
D_contig35538_46
140,8
barc0110
145,6
Excalibur_c17489_843
147,9
Kukri_rep_c69008_811
150,3
cfd0010c
158,0
Kukri_c35049_357
163,9
D_GCE8AKX02IXEFJ_281
165,4
BS00022267_51
BS00084133_51
166,2
BS00089597_51
167,0
BobWhite_c20748_123
barc0144
167,8
BS00011935_51
Excalibur_rep_c106082_272
Kukri_c48936_107
RAC875_c34968_514
wsnp_Ex_c11055_17928283
wsnp_Ex_c24145_33394561
wsnp_Ex_c24145_33394644
wsnp_Ex_c6657_11523455
gwm0272

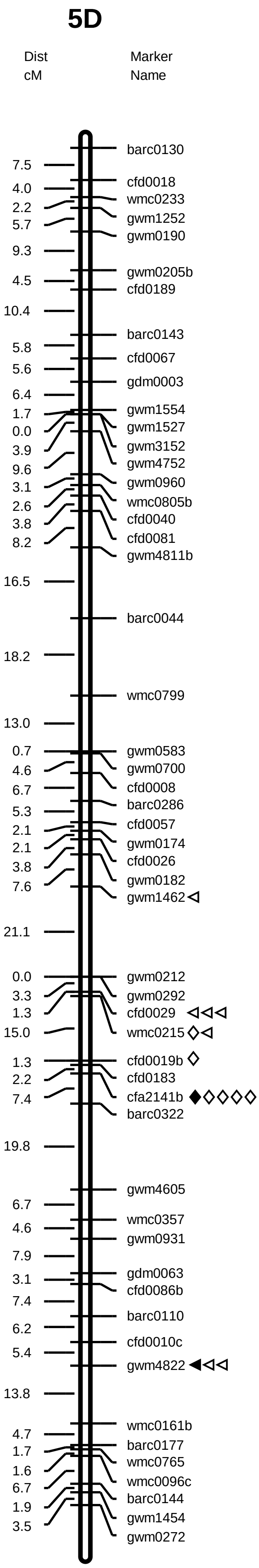
5D
Dist
cM
Marker
Name
barc0130
7.5
cfd0018
4.0
wmc0233
2.2
gwm1252
5.7
gwm0190
9.3
gwm0205b
4.5
cfd0189
10.4
barc0143
5.8
cfd0067
5.6
gdm0003
6.4
gwm1554
1.7
gwm1527
0.0
gwm3152
3.9
gwm4752
9.6
gwm0960
3.1
wmc0805b
2.6
cfd0040
3.8
cfd0081
8.2
gwm4811b
16.5
barc0044
18.2
wmc0799
13.0
gwm0583
0.7
gwm0700
4.6
cfd0008
6.7
barc0286
5.3
cfd0057
2.1
gwm0174
2.1
cfd0026
3.8
gwm0182
7.6
gwm1462
21.1
gwm0212
0.0
gwm0292
3.3
cfd0029
1.3
wmc0215
15.0
cfd0019b
1.3
cfd0183
2.2
cfa2141b
7.4
barc0322
19.8
gwm4605
6.7
wmc0357
4.6
gwm0931
7.9
gdm0063
3.1
cfd0086b
7.4
barc0110
6.2
cfd0010c
5.4
gwm4822
13.8
wmc0161b
4.7
barc0177
1.7
wmc0765
1.6
wmc0096c
6.7
barc0144
1.9
gwm1454
3.5
gwm0272

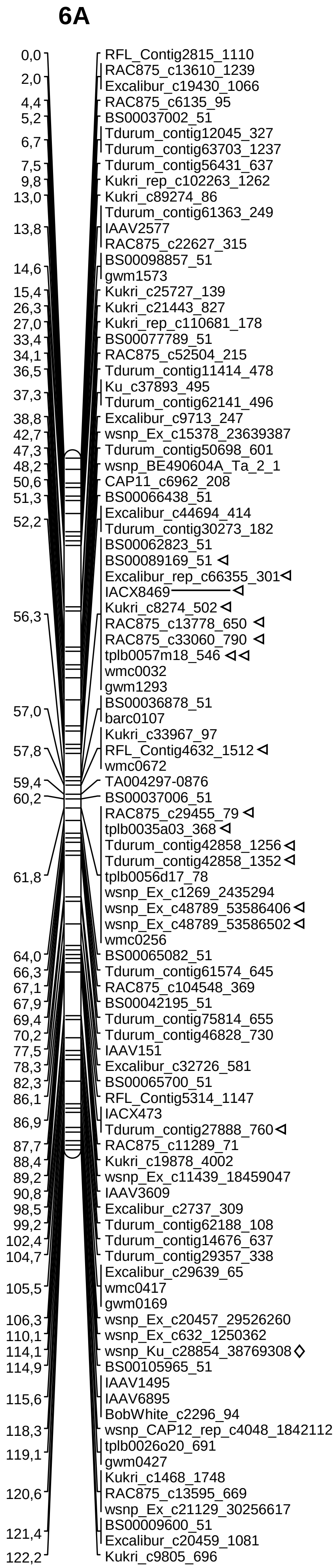
6A
0,0
2,0
4,4
5,2
6,7
7,5
9,8
13,0
13,8
14,6
15,4
26,3
27,0
33,4
34,1
36,5
37,3
38,8
42,7
47,3
48,2
50,6
51,3
52,2
56,3
57,0
57,8
59,4
60,2
61,8
64,0
66,3
67,1
67,9
69,4
70,2
77,5
78,3
82,3
86,1
86,9
87,7
88,4
89,2
90,8
98,5
99,2
102,4
104,7
105,5
106,3
110,1
114,1
114,9
115,6
118,3
119,1
120,6
121,4
122,2
RFL_Contig2815_1110
RAC875_c13610_1239
Excalibur_c19430_1066
RAC875_c6135_95
BS00037002_51
Tdurum_contig12045_327
Tdurum_contig63703_1237
Tdurum_contig56431_637
Kukri_rep_c102263_1262
Kukri_c89274_86
Tdurum_contig61363_249
IAAV2577
RAC875_c22627_315
BS00098857_51
gwm1573
Kukri_c25727_139
Kukri_c21443_827
Kukri_rep_c110681_178
BS00077789_51
RAC875_c52504_215
Tdurum_contig11414_478
Ku_c37893_495
Tdurum_contig62141_496
Excalibur_c9713_247
wsnp_Ex_c15378_23639387
Tdurum_contig50698_601
wsnp_BE490604A_Ta_2_1
CAP11_c6962_208
BS00066438_51
Excalibur_c44694_414
Tdurum_contig30273_182
BS00062823_51
BS00089169_51 ⊲
Excalibur_rep_c66355_301⊲
IACX8469 ⊲
Kukri_c8274_502 ⊲
RAC875_c13778_650 ⊲
RAC875_c33060_790 ⊲
tplb0057m18_546 ⊲⊲
wmc0032
gwm1293
BS00036878_51
barc0107
Kukri_c33967_97
RFL_Contig4632_1512 ⊲
wmc0672
TA004297-0876
BS00037006_51
RAC875_c29455_79 ⊲
tplb0035a03_368 ⊲
Tdurum_contig42858_1256 ⊲
Tdurum_contig42858_1352 ⊲
tplb0056d17_78
wsnp_Ex_c1269_2435294
wsnp_Ex_c48789_53586406 ⊲
wsnp_Ex_c48789_53586502 ⊲
wmc0256
BS00065082_51
Tdurum_contig61574_645
RAC875_c104548_369
BS00042195_51
Tdurum_contig75814_655
Tdurum_contig46828_730
IAAV151
Excalibur_c32726_581
BS00065700_51
RFL_Contig5314_1147
IACX473
Tdurum_contig27888_760 ⊲
RAC875_c11289_71
Kukri_c19878_4002
wsnp_Ex_c11439_18459047
IAAV3609
Excalibur_c2737_309
Tdurum_contig62188_108
Tdurum_contig14676_637
Tdurum_contig29357_338
Excalibur_c29639_65
wmc0417
gwm0169
wsnp_Ex_c20457_29526260
wsnp_Ex_c632_1250362
wsnp_Ku_c28854_38769308 ◇
BS00105965_51
IAAV1495
IAAV6895
BobWhite_c2296_94
wsnp_CAP12_rep_c4048_1842112
tplb0026o20_691
gwm0427
Kukri_c1468_1748
RAC875_c13595_669
wsnp_Ex_c21129_30256617
BS00009600_51
Excalibur_c20459_1081
Kukri_c9805_696

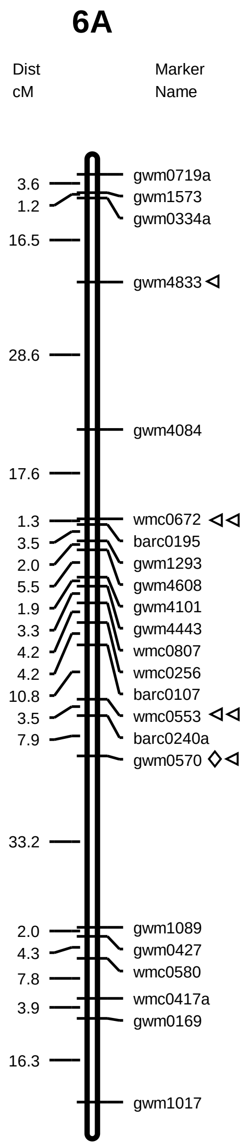
6A
Dist cM
Marker Name
3.6
1.2
16.5
28.6
17.6
1.3
3.5
2.0
5.5
1.9
3.3
4.2
4.2
10.8
3.5
7.9
33.2
2.0
4.3
7.8
3.9
16.3
gwm0719a
gwm1573
gwm0334a
gwm4833 ⊲
gwm4084
wmc0672 ⊲⊲
barc0195
gwm1293
gwm4608
gwm4101
gwm4443
wmc0807
wmc0256
barc0107
wmc0553 ⊲⊲
barc0240a
gwm0570 ◇⊲
gwm1089
gwm0427
wmc0580
wmc0417a
gwm0169
gwm1017

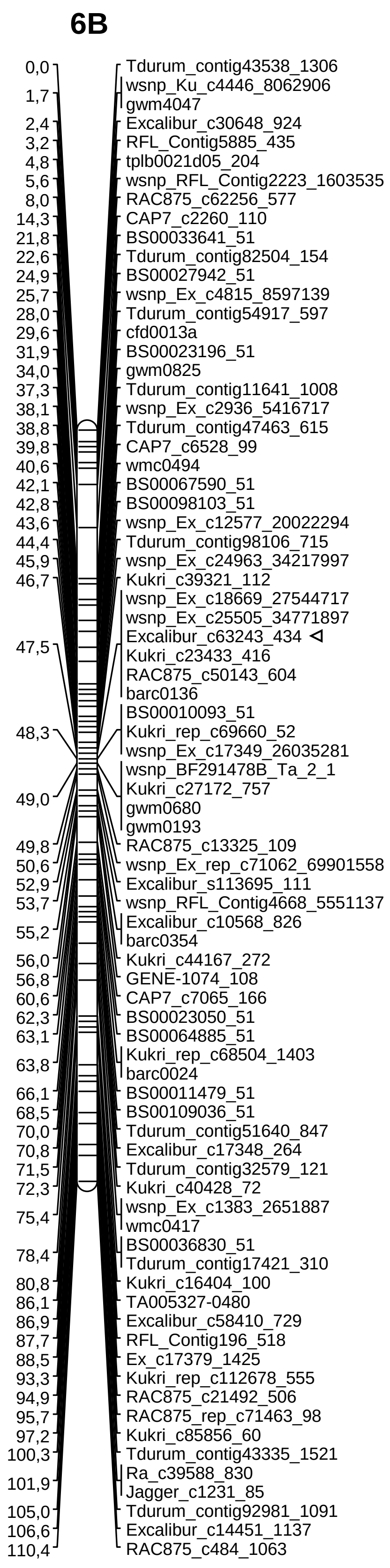
6B
0,0
1,7
2,4
3,2
4,8
5,6
8,0
14,3
21,8
22,6
24,9
25,7
28,0
29,6
31,9
34,0
37,3
38,1
38,8
39,8
40,6
42,1
42,8
43,6
44,4
45,9
46,7
47,5
48,3
49,0
49,8
50,6
52,9
53,7
55,2
56,0
56,8
60,6
62,3
63,1
63,8
66,1
68,5
70,0
70,8
71,5
72,3
75,4
78,4
80,8
86,1
86,9
87,7
88,5
93,3
94,9
95,7
97,2
100,3
101,9
105,0
106,6
110,4
Tdurum_contig43538_1306
wsnp_Ku_c4446_8062906
gwm4047
Excalibur_c30648_924
RFL_Contig5885_435
tplb0021d05_204
wsnp_RFL_Contig2223_1603535
RAC875_c62256_577
CAP7_c2260_110
BS00033641_51
Tdurum_contig82504_154
BS00027942_51
wsnp_Ex_c4815_8597139
Tdurum_contig54917_597
cfd0013a
BS00023196_51
gwm0825
Tdurum_contig11641_1008
wsnp_Ex_c2936_5416717
Tdurum_contig47463_615
CAP7_c6528_99
wmc0494
BS00067590_51
BS00098103_51
wsnp_Ex_c12577_20022294
Tdurum_contig98106_715
wsnp_Ex_c24963_34217997
Kukri_c39321_112
wsnp_Ex_c18669_27544717
wsnp_Ex_c25505_34771897
Excalibur_c63243_434 ◁
Kukri_c23433_416
RAC875_c50143_604
barc0136
BS00010093_51
Kukri_rep_c69660_52
wsnp_Ex_c17349_26035281
wsnp_BF291478B_Ta_2_1
Kukri_c27172_757
gwm0680
gwm0193
RAC875_c13325_109
wsnp_Ex_rep_c71062_69901558
Excalibur_s113695_111
wsnp_RFL_Contig4668_5551137
Excalibur_c10568_826
barc0354
Kukri_c44167_272
GENE-1074_108
CAP7_c7065_166
BS00023050_51
BS00064885_51
Kukri_rep_c68504_1403
barc0024
BS00011479_51
BS00109036_51
Tdurum_contig51640_847
Excalibur_c17348_264
Tdurum_contig32579_121
Kukri_c40428_72
wsnp_Ex_c1383_2651887
wmc0417
BS00036830_51
Tdurum_contig17421_310
Kukri_c16404_100
TA005327-0480
Excalibur_c58410_729
RFL_Contig196_518
Ex_c17379_1425
Kukri_rep_c112678_555
RAC875_c21492_506
RAC875_rep_c71463_98
Kukri_c85856_60
Tdurum_contig43335_1521
Ra_c39588_830
Jagger_c1231_85
Tdurum_contig92981_1091
Excalibur_c14451_1137
RAC875_c484_1063

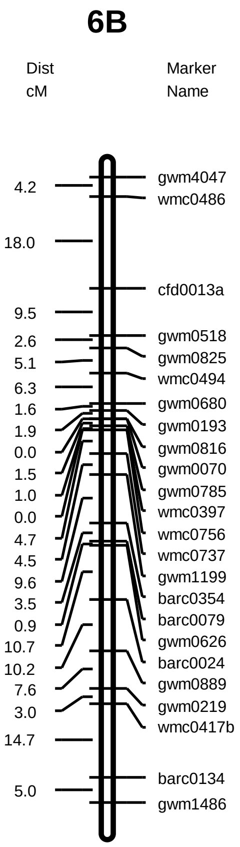
6B
Dist
cM
Marker
Name
4.2
18.0
9.5
2.6
5.1
6.3
1.6
1.9
0.0
1.5
1.0
0.0
4.7
4.5
9.6
3.5
0.9
10.7
10.2
7.6
3.0
14.7
5.0
gwm4047
wmc0486
cfd0013a
gwm0518
gwm0825
wmc0494
gwm0680
gwm0193
gwm0816
gwm0070
gwm0785
wmc0397
wmc0756
wmc0737
gwm1199
barc0354
barc0079
gwm0626
barc0024
gwm0889
gwm0219
wmc0417b
barc0134
gwm1486

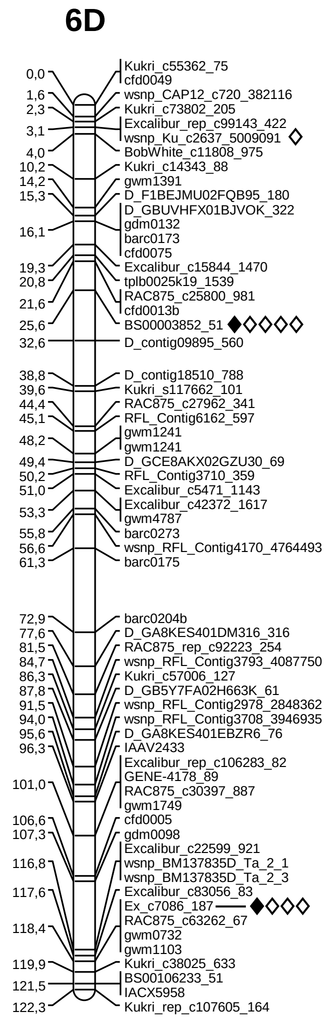
6D
0,0
1,6
2,3
3,1
4,0
10,2
14,2
15,3
16,1
19,3
20,8
21,6
25,6
32,6
38,8
39,6
44,4
45,1
48,2
49,4
50,2
51,0
53,3
55,8
56,6
61,3
72,9
77,6
81,5
84,7
86,3
87,8
91,5
94,0
95,6
96,3
101,0
106,6
107,3
116,8
117,6
118,4
119,9
121,5
122,3
Kukri_c55362_75
cfd0049
wsnp_CAP12_c720_382116
Kukri_c73802_205
Excalibur_rep_c99143_422
wsnp_Ku_c2637_5009091
BobWhite_c11808_975
Kukri_c14343_88
gwm1391
D_F1BEJMU02FQB95_180
D_GBUVHFX01BJVOK_322
gdm0132
barc0173
cfd0075
Excalibur_c15844_1470
tplb0025k19_1539
RAC875_c25800_981
cfd0013b
BS00003852_51
D_contig09895_560
D_contig18510_788
Kukri_s117662_101
RAC875_c27962_341
RFL_Contig6162_597
gwm1241
gwm1241
D_GCE8AKX02GZU30_69
RFL_Contig3710_359
Excalibur_c5471_1143
Excalibur_c42372_1617
gwm4787
barc0273
wsnp_RFL_Contig4170_4764493
barc0175
barc0204b
D_GA8KES401DM316_316
RAC875_rep_c92223_254
wsnp_RFL_Contig3793_4087750
Kukri_c57006_127
D_GB5Y7FA02H663K_61
wsnp_RFL_Contig2978_2848362
wsnp_RFL_Contig3708_3946935
D_GA8KES401EBZR6_76
IAAV2433
Excalibur_rep_c106283_82
GENE-4178_89
RAC875_c30397_887
gwm1749
cfd0005
gdm0098
Excalibur_c22599_921
wsnp_BM137835D_Ta_2_1
wsnp_BM137835D_Ta_2_3
Excalibur_c83056_83
Ex_c7086_187
RAC875_c63262_67
gwm0732
gwm1103
Kukri_c38025_633
BS00106233_51
IACX5958
Kukri_rep_c107605_164

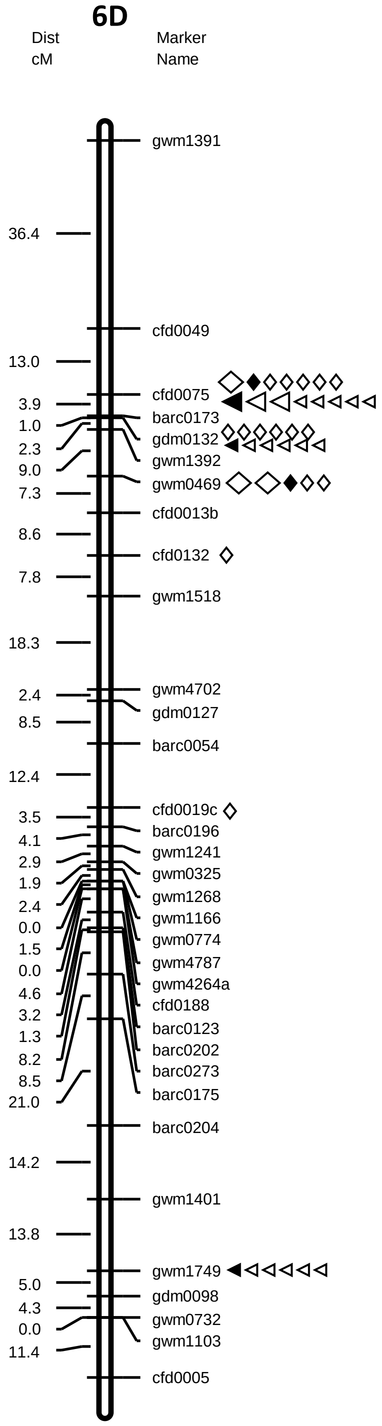
6D
Dist cM
Marker Name
36.4
13.0
3.9
1.0
2.3
9.0
7.3
8.6
7.8
18.3
2.4
8.5
12.4
3.5
4.1
2.9
1.9
2.4
0.0
1.5
0.0
4.6
3.2
1.3
8.2
8.5
21.0
14.2
13.8
5.0
4.3
0.0
11.4
gwm1391
cfd0049
cfd0075
barc0173
gdm0132
gwm1392
gwm0469
cfd0013b
cfd0132
gwm1518
gwm4702
gdm0127
barc0054
cfd0019c
barc0196
gwm1241
gwm0325
gwm1268
gwm1166
gwm0774
gwm4787
gwm4264a
cfd0188
barc0123
barc0202
barc0273
barc0175
barc0204
gwm1401
gwm1749
gdm0098
gwm0732
gwm1103
cfd0005

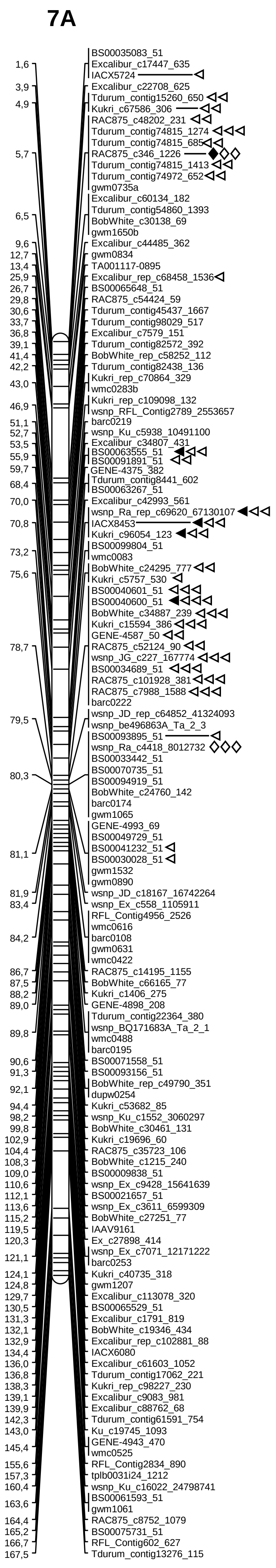
7A
1,6
3,9
4,9
5,7
6,5
9,6
12,7
13,4
25,9
26,7
29,8
30,6
33,7
36,8
39,1
41,4
42,2
43,0
46,9
51,1
52,7
53,5
55,9
59,7
68,4
70,0
70,8
73,2
75,6
78,7
79,5
80,3
81,1
81,9
83,4
84,2
86,7
87,5
88,2
89,0
89,8
90,6
91,3
92,1
94,4
98,2
99,8
102,9
104,4
108,3
109,0
110,6
112,1
113,6
115,2
119,5
120,3
121,1
124,1
124,8
129,7
130,5
131,3
132,1
132,9
134,4
136,0
136,8
138,3
139,1
139,9
142,3
143,0
145,4
155,6
157,3
160,4
163,6
164,4
165,2
166,7
167,5
BS00035083_51
Excalibur_c17447_635
IACX5724
Excalibur_c22708_625
Tdurum_contig15260_650
Kukri_c67586_306
RAC875_c48202_231
Tdurum_contig74815_1274
Tdurum_contig74815_685
RAC875_c346_1226
Tdurum_contig74815_1413
Tdurum_contig74972_652
gwm0735a
Excalibur_c60134_182
Tdurum_contig54860_1393
BobWhite_c30138_69
gwm1650b
Excalibur_c44485_362
gwm0834
TA001117-0895
Excalibur_rep_c68458_1536
BS00065648_51
RAC875_c54424_59
Tdurum_contig45437_1667
Tdurum_contig98029_517
Excalibur_c7579_151
Tdurum_contig82572_392
BobWhite_rep_c58252_112
Tdurum_contig82438_136
Kukri_rep_c70864_329
wmc0283b
Kukri_rep_c109098_132
wsnp_RFL_Contig2789_2553657
barc0219
wsnp_Ku_c5938_10491100
Excalibur_c34807_431
BS00063555_51
BS00091891_51
GENE-4375_382
Tdurum_contig8441_602
BS00063267_51
Excalibur_c42993_561
wsnp_Ra_rep_c69620_67130107
IACX8453
Kukri_c96054_123
BS00099804_51
wmc0083
BobWhite_c24295_777
Kukri_c5757_530
BS00040601_51
BS00040600_51
BobWhite_c34887_239
Kukri_c15594_386
GENE-4587_50
RAC875_c52124_90
wsnp_JG_c227_167774
BS00034689_51
RAC875_c101928_381
RAC875_c7988_1588
barc0222
wsnp_JD_rep_c64852_41324093
wsnp_be496863A_Ta_2_3
BS00093895_51
wsnp_Ra_c4418_8012732
BS00033442_51
BS00070735_51
BS00094919_51
BobWhite_c24760_142
barc0174
gwm1065
GENE-4993_69
BS00049729_51
BS00041232_51
BS00030028_51
gwm1532
gwm0890
wsnp_JD_c18167_16742264
wsnp_Ex_c558_1105911
RFL_Contig4956_2526
wmc0616
barc0108
gwm0631
wmc0422
RAC875_c14195_1155
BobWhite_c66165_77
Kukri_c1406_275
GENE-4898_208
Tdurum_contig22364_380
wsnp_BQ171683A_Ta_2_1
wmc0488
barc0195
BS00071558_51
BS00093156_51
BobWhite_rep_c49790_351
dupw0254
Kukri_c53682_85
wsnp_Ku_c1552_3060297
BobWhite_c30461_131
Kukri_c19696_60
RAC875_c35723_106
BobWhite_c1215_240
BS00009838_51
wsnp_Ex_c9428_15641639
BS00021657_51
wsnp_Ex_c3611_6599309
BobWhite_c27251_77
IAAV9161
Ex_c27898_414
wsnp_Ex_c7071_12171222
barc0253
Kukri_c40735_318
gwm1207
Excalibur_c113078_320
BS00065529_51
Excalibur_c1791_819
BobWhite_c19346_434
Excalibur_rep_c102881_88
IACX6080
Excalibur_c61603_1052
Tdurum_contig17062_221
Kukri_rep_c98227_230
Excalibur_c9083_981
Excalibur_c88762_68
Tdurum_contig61591_754
Ku_c19745_1093
GENE-4943_470
wmc0525
RFL_Contig2834_890
tplb0031i24_1212
wsnp_Ku_c16022_24798741
BS00061593_51
gwm1061
RAC875_c8752_1079
BS00075731_51
RFL_Contig602_627
Tdurum_contig13276_115

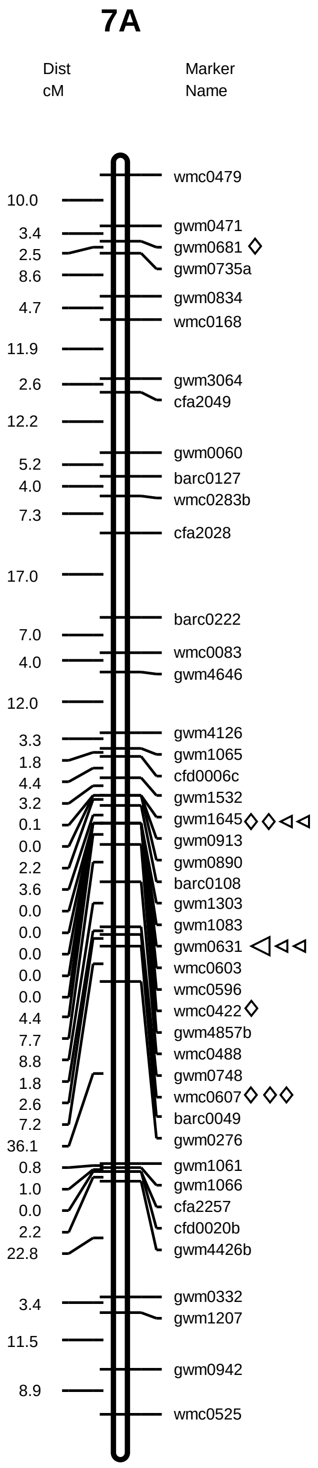
7A
Dist
cM
Marker
Name
10.0
3.4
2.5
8.6
4.7
11.9
2.6
12.2
5.2
4.0
7.3
17.0
7.0
4.0
12.0
3.3
1.8
4.4
3.2
0.1
0.0
2.2
3.6
0.0
0.0
0.0
0.0
0.0
4.4
7.7
8.8
1.8
2.6
7.2
36.1
0.8
1.0
0.0
2.2
22.8
3.4
11.5
8.9
wmc0479
gwm0471
gwm0681
gwm0735a
gwm0834
wmc0168
gwm3064
cfa2049
gwm0060
barc0127
wmc0283b
cfa2028
barc0222
wmc0083
gwm4646
gwm4126
gwm1065
cfd0006c
gwm1532
gwm1645
gwm0913
gwm0890
barc0108
gwm1303
gwm1083
gwm0631
wmc0603
wmc0596
wmc0422
gwm4857b
wmc0488
gwm0748
wmc0607
barc0049
gwm0276
gwm1061
gwm1066
cfa2257
cfd0020b
gwm4426b
gwm0332
gwm1207
gwm0942
wmc0525

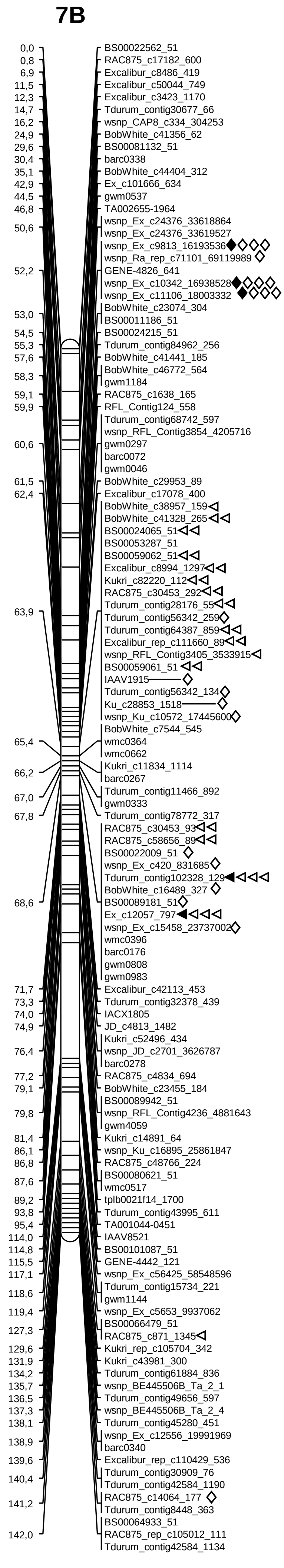
7B
0,0 BS00022562_51
0,8 RAC875_c17182_600
6,9 Excalibur_c8486_419
11,5 Excalibur_c50044_749
12,3 Excalibur_c3423_1170
14,7 Tdurum_contig30677_66
16,2 wsnp_CAP8_c334_304253
24,9 BobWhite_c41356_62
29,6 BS00081132_51
30,4 barc0338
35,1 BobWhite_c44404_312
42,9 Ex_c101666_634
44,5 gwm0537
46,8 TA002655-1964
50,6 wsnp_Ex_c24376_33618864
wsnp_Ex_c24376_33619527
wsnp_Ex_c9813_16193536
wsnp_Ra_rep_c71101_69119989
52,2 GENE-4826_641
wsnp_Ex_c10342_16938528
wsnp_Ex_c11106_18003332
53,0 BobWhite_c23074_304
BS00011186_51
54,5 BS00024215_51
55,3 Tdurum_contig84962_256
57,6 BobWhite_c41441_185
58,3 BobWhite_c46772_564
gwm1184
59,1 RAC875_c1638_165
59,9 RFL_Contig124_558
Tdurum_contig68742_597
wsnp_RFL_Contig3854_4205716
60,6 gwm0297
barc0072
gwm0046
61,5 BobWhite_c29953_89
62,4 Excalibur_c17078_400
BobWhite_c38957_159
BobWhite_c41328_265
BS00024065_51
BS00053287_51
BS00059062_51
Excalibur_c8994_1297
Kukri_c82220_112
RAC875_c30453_292
Tdurum_contig28176_55
63,9 Tdurum_contig56342_259
Tdurum_contig64387_859
Excalibur_rep_c111660_89
wsnp_RFL_Contig3405_3533915
BS00059061_51
IAAV1915
Tdurum_contig56342_134
Ku_c28853_1518
wsnp_Ku_c10572_17445600
BobWhite_c7544_545
65,4 wmc0364
wmc0662
Kukri_c11834_1114
66,2 barc0267
Tdurum_contig11466_892
67,0 gwm0333
67,8 Tdurum_contig78772_317
RAC875_c30453_93
RAC875_c58656_89
BS00022009_51
wsnp_Ex_c420_831685
Tdurum_contig102328_129
BobWhite_c16489_327
68,6 BS00089181_51
Ex_c12057_797
wsnp_Ex_c15458_23737002
wmc0396
barc0176
gwm0808
gwm0983
71,7 Excalibur_c42113_453
73,3 Tdurum_contig32378_439
74,0 IACX1805
74,9 JD_c4813_1482
Kukri_c52496_434
76,4 wsnp_JD_c2701_3626787
barc0278
77,2 RAC875_c4834_694
79,1 BobWhite_c23455_184
BS00089942_51
79,8 wsnp_RFL_Contig4236_4881643
gwm4059
81,4 Kukri_c14891_64
86,1 wsnp_Ku_c16895_25861847
86,8 RAC875_c48766_224
87,6 BS00080621_51
wmc0517
89,2 tplb0021f14_1700
93,8 Tdurum_contig43995_611
95,4 TA001044-0451
114,0 IAAV8521
114,8 BS00101087_51
115,5 GENE-4442_121
117,1 wsnp_Ex_c56425_58548596
118,6 Tdurum_contig15734_221
gwm1144
119,4 wsnp_Ex_c5653_9937062
BS00066479_51
127,3 RAC875_c871_1345
129,6 Kukri_rep_c105704_342
131,9 Kukri_c43981_300
134,2 Tdurum_contig61884_836
135,7 wsnp_BE445506B_Ta_2_1
136,5 Tdurum_contig49656_597
137,3 wsnp_BE445506B_Ta_2_4
138,1 Tdurum_contig45280_451
138,9 wsnp_Ex_c12556_19991969
barc0340
139,6 Excalibur_rep_c110429_536
140,4 Tdurum_contig30909_76
Tdurum_contig42584_1190
141,2 RAC875_c14064_177
Tdurum_contig8448_363
142,0 BS00064933_51
RAC875_rep_c105012_111
Tdurum_contig42584_1134

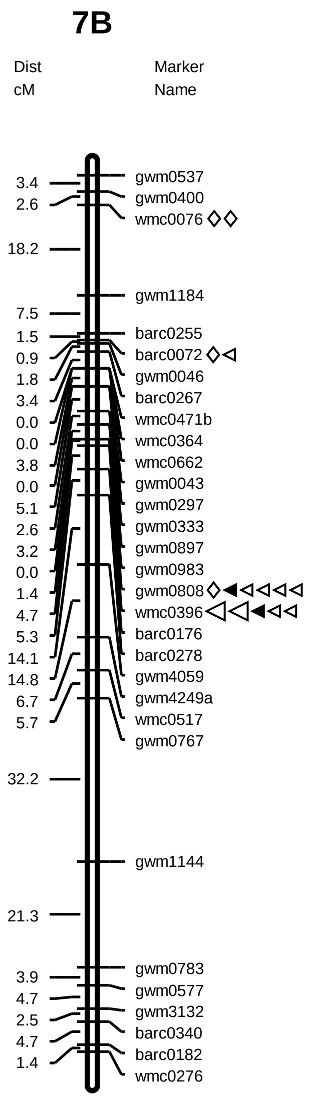
7B
Dist cM
Marker Name
3.4 gwm0537
2.6 gwm0400
18.2 wmc0076
7.5 gwm1184
1.5 barc0255
0.9 barc0072
1.8 gwm0046
3.4 barc0267
0.0 wmc0471b
0.0 wmc0364
3.8 wmc0662
0.0 gwm0043
5.1 gwm0297
2.6 gwm0333
3.2 gwm0897
0.0 gwm0983
1.4 gwm0808
4.7 wmc0396
5.3 barc0176
14.1 barc0278
14.8 gwm4059
6.7 gwm4249a
5.7 wmc0517
32.2 gwm0767
gwm1144
21.3
3.9 gwm0783
4.7 gwm0577
2.5 gwm3132
4.7 barc0340
1.4 barc0182
wmc0276

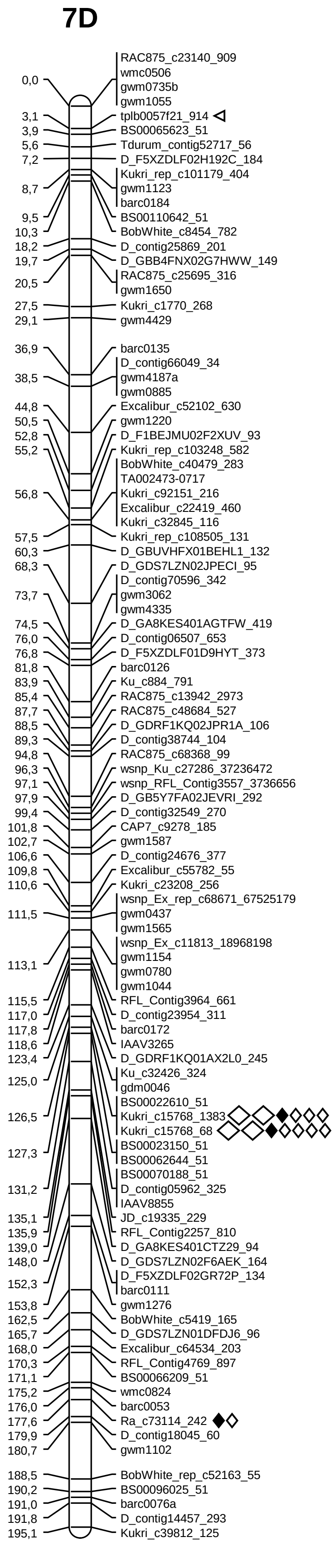

7D
RAC875_c23140_909
wmc0506
gwm0735b
gwm1055
tplb0057f21_914
BS00065623_51
Tdurum_contig52717_56
D_F5XZDLF02H192C_184
Kukri_rep_c101179_404
gwm1123
barc0184
BS00110642_51
BobWhite_c8454_782
D_contig25869_201
D_GBB4FNX02G7HWW_149
RAC875_c25695_316
gwm1650
Kukri_c1770_268
gwm4429
barc0135
D_contig66049_34
gwm4187a
gwm0885
Excalibur_c52102_630
gwm1220
D_F1BEJMU02F2XUV_93
Kukri_rep_c103248_582
BobWhite_c40479_283
TA002473-0717
Kukri_c92151_216
Excalibur_c22419_460
Kukri_c32845_116
Kukri_rep_c108505_131
D_GBUVHFX01BEHL1_132
D_GDS7LZN02JPECI_95
D_contig70596_342
gwm3062
gwm4335
D_GA8KES401AGTFW_419
D_contig06507_653
D_F5XZDLF01D9HYT_373
barc0126
Ku_c884_791
RAC875_c13942_2973
RAC875_c48684_527
D_GDRF1KQ02JPR1A_106
D_contig38744_104
RAC875_c68368_99
wsnp_Ku_c27286_37236472
wsnp_RFL_Contig3557_3736656
D_GB5Y7FA02JEVRI_292
D_contig32549_270
CAP7_c9278_185
gwm1587
D_contig24676_377
Excalibur_c55782_55
Kukri_c23208_256
wsnp_Ex_rep_c68671_67525179
gwm0437
gwm1565
wsnp_Ex_c11813_18968198
gwm1154
gwm0780
gwm1044
RFL_Contig3964_661
D_contig23954_311
barc0172
IAAV3265
D_GDRF1KQ01AX2L0_245
Ku_c32426_324
gdm0046
BS00022610_51
Kukri_c15768_1383
Kukri_c15768_68
BS00023150_51
BS00062644_51
BS00070188_51
D_contig05962_325
IAAV8855
JD_c19335_229
RFL_Contig2257_810
D_GA8KES401CTZ29_94
D_GDS7LZN02F6AEK_164
D_F5XZDLF02GR72P_134
barc0111
gwm1276
BobWhite_c5419_165
D_GDS7LZN01DFDJ6_96
Excalibur_c64534_203
RFL_Contig4769_897
BS00066209_51
wmc0824
barc0053
Ra_c73114_242
D_contig18045_60
gwm1102
BobWhite_rep_c52163_55
BS00096025_51
barc0076a
D_contig14457_293
Kukri_c39812_125
0,0
3,1
3,9
5,6
7,2
8,7
9,5
10,3
18,2
19,7
20,5
27,5
29,1
36,9
38,5
44,8
50,5
52,8
55,2
56,8
57,5
60,3
68,3
73,7
74,5
76,0
76,8
81,8
83,9
85,4
87,7
88,5
89,3
94,8
96,3
97,1
97,9
99,4
101,8
102,7
106,6
109,8
110,6
111,5
113,1
115,5
117,0
117,8
118,6
123,4
125,0
126,5
127,3
131,2
135,1
135,9
139,0
148,0
152,3
153,8
162,5
165,7
168,0
170,3
171,1
175,2
176,0
177,6
179,9
180,7
188,5
190,2
191,0
191,8
195,1

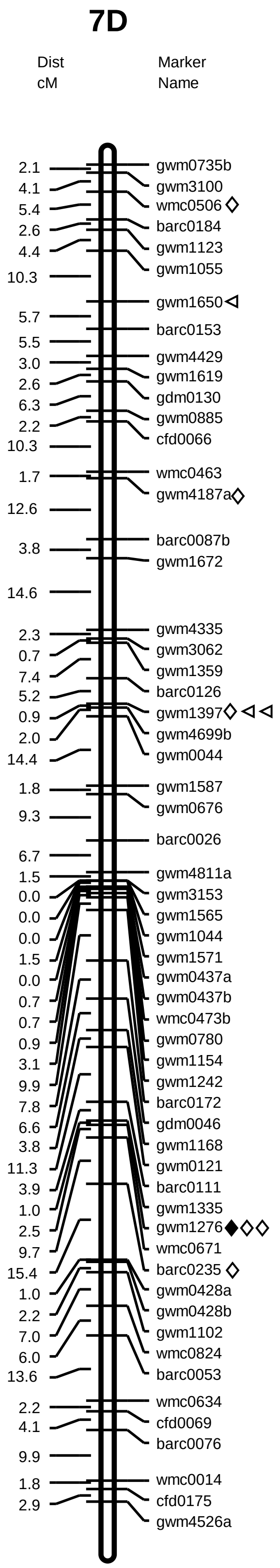

7D
Dist
cM
Marker
Name
2.1
4.1
5.4
2.6
4.4
10.3
5.7
5.5
3.0
2.6
6.3
2.2
10.3
1.7
12.6
3.8
14.6
2.3
0.7
7.4
5.2
0.9
2.0
14.4
1.8
9.3
6.7
1.5
0.0
0.0
0.0
1.5
0.0
0.7
0.7
0.9
3.1
9.9
7.8
6.6
3.8
11.3
3.9
1.0
2.5
9.7
15.4
1.0
2.2
7.0
6.0
13.6
2.2
4.1
9.9
1.8
2.9
gwm0735b
gwm3100
wmc0506
barc0184
gwm1123
gwm1055
gwm1650
barc0153
gwm4429
gwm1619
gdm0130
gwm0885
cfd0066
wmc0463
gwm4187a
barc0087b
gwm1672
gwm4335
gwm3062
gwm1359
barc0126
gwm1397
gwm4699b
gwm0044
gwm1587
gwm0676
barc0026
gwm4811a
gwm3153
gwm1565
gwm1044
gwm1571
gwm0437a
gwm0437b
wmc0473b
gwm0780
gwm1154
gwm1242
barc0172
gdm0046
gwm1168
gwm0121
barc0111
gwm1335
gwm1276
wmc0671
barc0235
gwm0428a
gwm0428b
gwm1102
wmc0824
barc0053
wmc0634
cfd0069
barc0076
wmc0014
cfd0175
gwm4526a
