# Supplementary material for: Analysis of main effect QTL for thousand grain weight in European winter wheat (Triticum aestivum L.) by genome-wide association mapping
Source: Front Plant Sci. 2015 Sep 1;6:644. doi: 10.3389/fpls.2015.00644 (PMC4555037; doi:10.3389/fpls.2015.00644)
Supplement: Supplementary file 1 [file DataSheet1.ZIP › Supplementary/152871_Röder_Image_2.PDF]

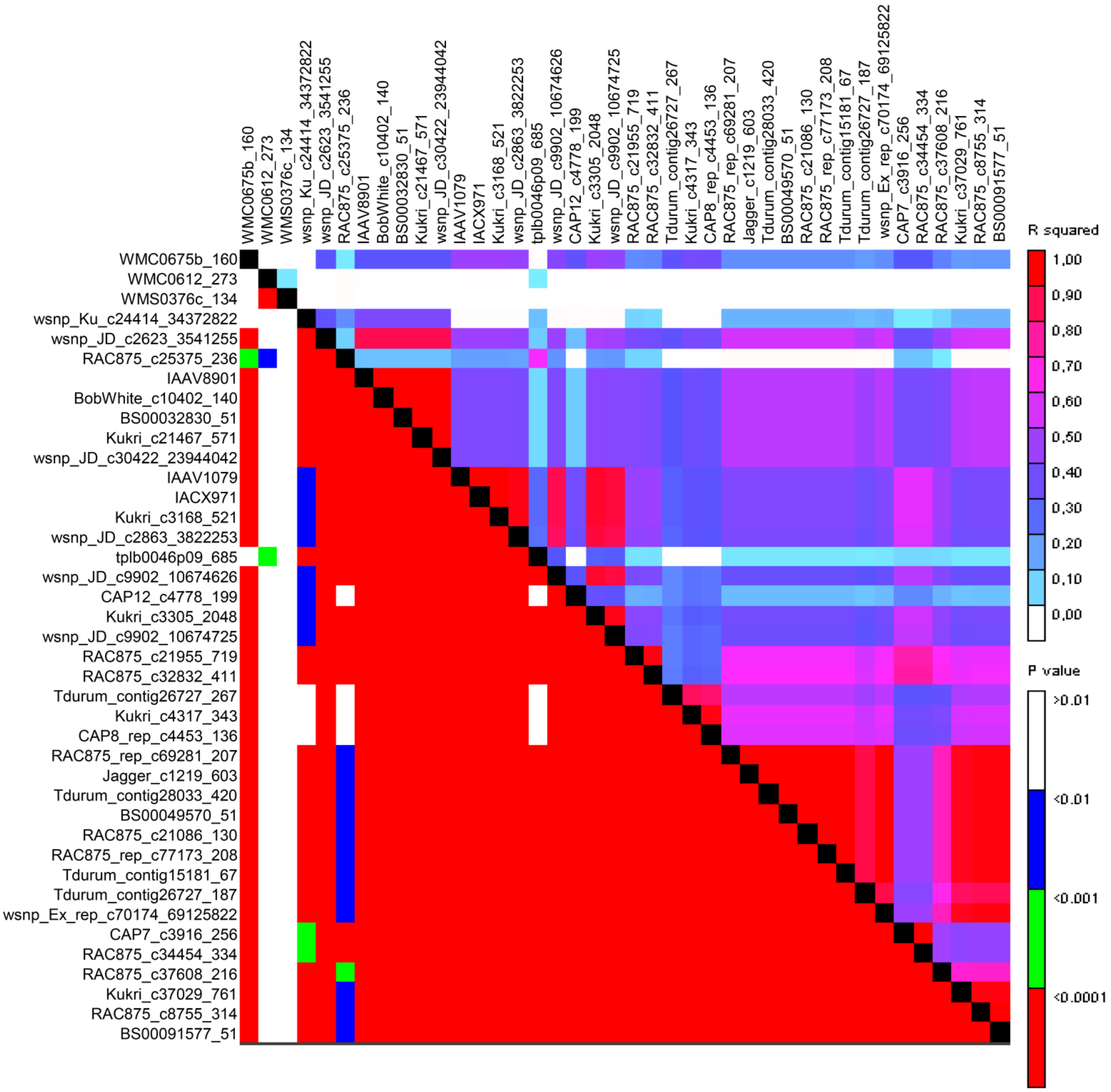

Supplemental file 16: Linkage disequilibrium was detected between the significant allele GWM0675b_160 and a cluster of SNP-marker on chromosome 3B, significant in several environments ranging from wsnp_Ku_c24414_34372822 to BS00091577_51.
